# Crystallographic fragment screening reveals ligand hotspots in TRIM21 PRY-SPRY domain

**DOI:** 10.1038/s42004-025-01574-3

**Published:** 2025-06-13

**Authors:** Yeojin Kim, Aleksandar Lučić, Christopher Lenz, Frederic Farges, Martin P. Schwalm, Krishna Saxena, Thomas Hanke, Peter G. Marples, Jasmin C. Aschenbrenner, Daren Fearon, Frank von Delft, Andreas Krämer, Stefan Knapp

**Affiliations:** 1 Institute of Pharmaceutical Chemistry, Goethe University, Frankfurt am Main, Germany; 2 Structural Genomics Consortium, Buchmann Institute of Molecular Life Sciences (BMLS), Frankfurt am Main, Germany; 3 German Translational Cancer Consortium (DKTK), Frankfurt am Main, Germany; 4 Diamond Light Source Ltd, Harwell Science and Innovation Campus, Didcot, UK; 5 Research Complex at Harwell, Harwell Science and Innovation Campus, Didcot, UK; 6 Centre for Medicines Discovery, NDM Research Building, University of Oxford, Oxford, UK; 7 Frankfurt Cancer Institute, Goethe University, Frankfurt am Main, Germany

**Keywords:** X-ray crystallography, Structure-based drug design, Virtual screening, Drug discovery and development

## Abstract

Tripartite motif-containing protein 21 (TRIM21), and particularly its PRY-SPRY protein interaction domain, plays a critical role in the immune response by recognizing intracellular antibodies targeting them for degradation. In this study, we performed a crystallographic fragment screening (CFS) campaign to identify potential small molecule binders targeting the PRY-SPRY domain of TRIM21. Our screen identified a total of 109 fragments binding to TRIM21 that were distributed across five distinct binding sites. These fragments have been designed to facilitate straightforward follow-up chemistry, making them ideal starting points for further chemical optimization. A subsequent fragment merging approach demonstrated improved activity. To enable functional validation of compounds with full length human TRIM21, we established a NanoBRET assay suitable for measuring target engagement to the main Fc binding site in life cells. The high-resolution structural data and observed binding modes across the different sites highlight the versatility of the PRY-SPRY domain as a target for small-molecule intervention. The presented data provide a solid foundation for structure-guided ligand design, enabling the rational design of more potent and selective compounds, with the goal to develop bivalent molecules such as Proteolysis Targeting Chimeras (PROTACs).

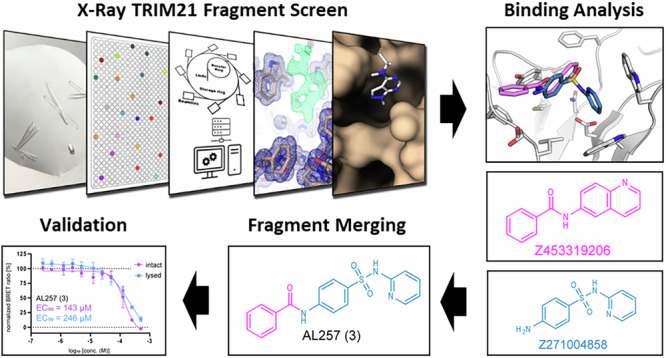

## Abstract (web summary)

The TRIM21 protein’s PRY-SPRY domain is crucial in immune response by recognizing and degrading intracellular antibodies, yet small-molecule targeting remains underexplored. Here, the authors use a crystallographic fragment screening to identify 109 fragment binders distributing across five distinct binding sites of TRIM21, improve the activity by a fragment merging approach, and establish a NanoBRET assay to enable functional validation of compounds.

## Introduction

Tripartite motif-containing protein 21 (TRIM21) is a member of the TRIM family, characterized by the presence of a RING finger domain, a B-box motif, a coiled-coil region, and a PRY-SPRY domain. The protein plays a multifaceted role in the immune response, primarily functioning as an E3 ubiquitin ligase mediating ubiquitination and subsequent proteasomal degradation of TRIM21-interacting proteins^1^. Within the TRIM family, it has a unique role due to its specific binding to the Fc region of intracellular antibodies (AB) and thereby marking antibody-coated proteins or non-enveloped virus particles for proteasomal degradation^2^. This specific function of TRIM21 has been exploited by the development of the TRIM-Away technology, which allows the selective and comparably rapid depletion of endogenous proteins in cells. A limitation of this method is that the specific AB has to be delivered exogenously into the cells by microinjection or electroporation making it not feasible for most therapeutic intervention to date^3–5^. Nevertheless, the method clearly demonstrated the potential of TRIM21 to be hijacked for targeted protein degradation if high affinity ligands can be developed, particularly in the light of recent advances in PROTAC or molecular glue design. Recently a study was published by Lu et al. presenting the first heterobifunctional degraders based on acepromazine as a TRIM21 ligand. However, the role of acepromazine as a sedative and neuroleptic drug and its modest activity for human wild type TRIM21 leave room for significant improvement of this ligand^6^.

Central to the function of TRIM21 is the PRY-SPRY domain, which mediates interactions with immunoglobulin Fc regions. This domain acts as a guiding module, targeting AB-bound complexes for ubiquitination and subsequent degradation^7^. The PRY-SPRY domain contains a deep binding pocket, making it the most druggable and therefore most promising domain of the TRIM21 holoenzyme for therapeutic intervention. The general druggability of the PRY-SPRY domains was further demonstrated recently for other TRIM protein such as TRIM58^8^ and TRIM7^9^.

Crystallographic fragment screening (CFS) has emerged as a powerful tool in drug discovery, particularly effective for identifying initial binding events^10–17^. In this study, we utilized CFS to uncover potential starting points for developing ligands targeting the PRY-SPRY domain of TRIM21. The DSi-poised library used was specifically designed to facilitate easy and fast follow-up chemistry to optimize these fragment hits into potent inhibitors via iterative structure-based drug design^18^.

## Results

In our efforts to identify initial chemical starting points for the development of human TRIM21 inhibitors, we began by exploring suitable crystallization conditions for the PRYS-PRY domain. Unfortunately, these attempts produced only very small crystals that were challenging to optimize or reproduce and diffracted only to low resolution. As a result, human TRIM21 proved to be a suboptimal candidate for crystallographic fragment screening (CFS), where high resolution and reproducibility are crucial^19,20^. Given the homology of the human and mouse orthologues (overall sequence identity = 75%, RMS of cα = 0.364 Å over 145 atoms) especially in their main binding pockets (84% sequence identity) (Fig. S1), we continued our fragment discovery project with the mouse protein as a surrogate, since a high-resolution structure had already been published (PDB code 2VOK^2^).

Although we were able to reproduce the published crystallization conditions and obtained comparable resolution (1.5–2.0 Å on average) in diffraction experiments, we concluded that this crystal form was not an ideal candidate for CFS for two reasons: first, the protein crystallized in the space group P2_1_ with two molecules in the asymmetric unit (ASU) and the packing appeared to be very tight with small solvent channels (crystal solvent content 35%), potentially causing problems in soaking experiments. Moreover, the two molecules were arranged “head-to-head” in the ASU, resulting in a blocked primary antibody binding site. In particular, Tyr455 blocked access to the binding site (Fig. S2). In addition, the binding site was already occupied by a 2-(N-morpholino)ethanesulfonic acid (MES) buffer molecule. Replacing MES resulted in the growth of significantly fewer crystals which had much poorer diffraction quality. We therefore decided to start a coarse screening campaign to identify more suitable crystallization conditions and crystal packing. Fortunately, we identified an ideal crystallization condition yielding crystals in the space group I4, diffracting to excellent average resolution (1.1–1.4 Å). The crystal packing had large solvent channels (crystal solvent content increased from 35 to 50%), and a solvent-exposed primary binding pocket, making it an ideal candidate for CFS (Fig. S2).

**Fig. 1:**
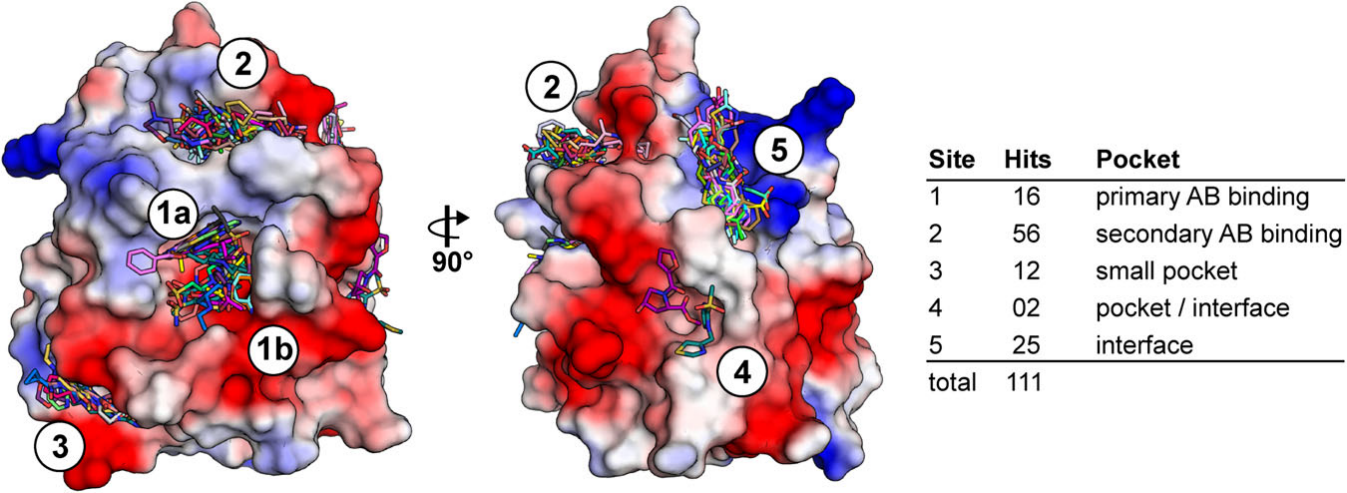
Overview of binding sites for all fragment hits on TRIM21 PRY-SPRY domain revealed by screening the DSi Poised library. The protein shown as electrostatic surface representation. The table indicates the number of fragments located in each of the binding sites.

However, initial fragment soaking trials revealed that high concentrations of DMSO (10–25% v/v) had a negative effect on crystal quality resulting in much poorer diffraction and overall data quality. Therefore, we screened the DSi-Poised library composed of 768 compounds dissolved in ethylene glycol. The final compound concentration in the drop was ~10 mM and the ethylene glycol concentration was around 10% (v/v), which was sufficient for cryo-protection of the crystals. The overall dataset quality was excellent. All measured datasets had highly similar cell parameters and very high resolution ranging from 1.15 to 1.57 Å with an average resolution of 1.29 Å (Table S2). No dataset was excluded due to crystal damage, insufficient resolution, or changes in cell parameters. Out of the initial 768 datasets we observed 130 binding events in PanDDA event maps^21^. 19 events were later rejected during refinement, mostly due to weak ligand electron density or poor fit of the ligand to the observed electron density. In total, we identified 109 distinct fragments with clearly interpretable electron density, corresponding to a hit rate of ~14%. These fragments were distributed across five distinct binding sites, with two fragments binding to more than one site, resulting in 111 total binding events (Fig. 1). Sixteen of the observed fragments bound to the primary antibody binding pocket (site #1), 56 were located in a cavity adjacent to the main binding pocket which also serves as the recruitment site for the Fc fragment of antibodies (site #2). In addition, 12 fragments were located within a small pocket (site #3) which has not been described as a ligand binding site before, and 2 fragments were found in another crevice (site #4). The remaining 25 fragments were bound to the surface (summarized as site #5). On closer inspection, sites #4 and #5 were located at crystal packing interfaces, creating a local environment in the crystal that likely influenced ligand binding and are therefore considered artefacts (Figs. 1 and 5).

### Site #1: primary antibody binding pocket

**Fig. 2:**
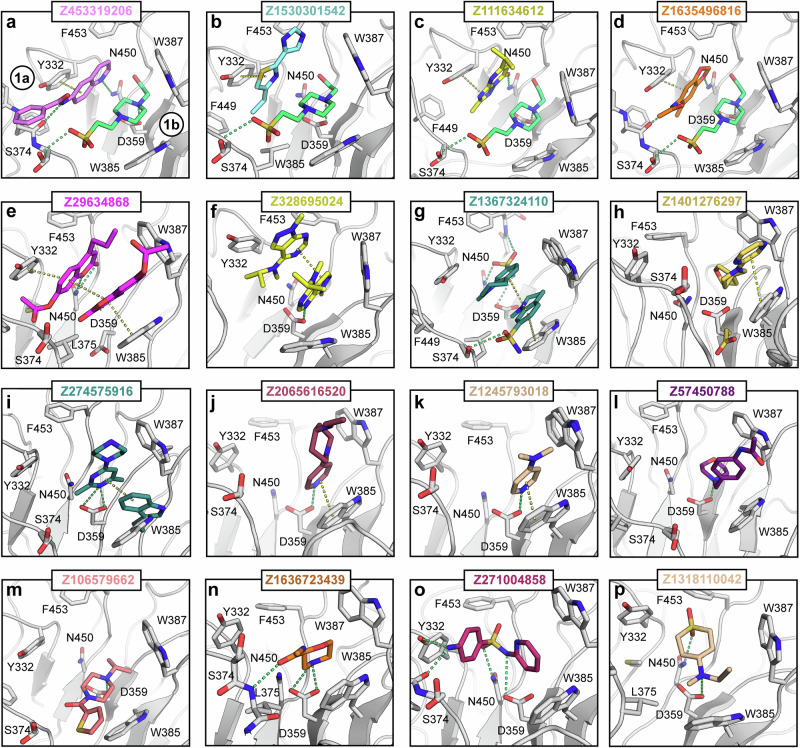
All 16 fragments bound to site #1. All hits targeting site #1 are shown in panels **a**–**p**. Polar interactions are depicted as green, π-π interactions as yellow dashed lines. In **a**, the two sub-binding sites, 1a and 1b, are indicated, but have been omitted in the other panels for clarity. Colored side chains indicate conformational changes between unbound and bound states, whereas gray represents conformation present in the apo-structure.

Site #1 is the primary binding site that mediates the antibody interaction (Fig. S3). The pocket is large in diameter compared to the other found sites, measuring ~ 13 ×15 Å in width and height. This feature is underscored by the observation that the pocket is large enough to accommodate two fragment molecules simultaneously, allowing us to distinguish two sub-binding sites within the main site #1 pocket (Fig. 1, site 1a and site 1b, Fig. 2a). In four structures, a fragment is bound next to a HEPES buffer molecule (Fig. 2a–d), which is also present in the apo structure, whereas in the other three cases the HEPES molecule is replaced by a second fragment molecule (Fig. 2e–g). In the remaining nine structures, a single molecule was bound by either replacing the HEPES molecule at sub-site 1b or occupying the entire pocket (Fig. 2o, p). Comparing the two sub-pocket binding sites with the Ig2a bound complex structure (PDB: 3ZO0^2^) provided interesting insights: Here, two residues (His433 and Asn434) located in the Fc region of the antibody were involved in a critical hotspot interaction with Asp359 and, to a lesser extent, Asn450 in the site #1 binding pocket of TRIM21. An overlay revealed that the two sub-binding sites align precisely with the location of these two amino acids (Fig. S3). It is worth mentioning that Asn450 is replaced by a serine in human TRIM21 (Fig. S1), however, according to the comprehensive study of Keeble et al. that does not influence the binding of the antibody significantly as the binding affinity is even maintained across mammals (mouse TRIM21 binds human IgG as well as mouse IgG and vice versa)^2^.

The binding site is characterized by a negative overall surface charge (Fig. S4), with Asp359 located at the base of the pocket, surrounded predominantly by hydrophobic and aromatic amino acids. A notable structural feature is the presence of two tryptophan residues (Trp385 and Trp387), which are positioned at an almost perfect right angle to each other and contribute to almost half of the flanking structure of the pocket. Mutation studies have highlighted the importance of these residues in particular, with mutations to alanine resulting in complete inhibition of AB binding^2^. On the opposite side of this binding pocket, the aromatic side chain of Tyr332 is positioned. Most fragments interacted either by forming polar contacts with the aforementioned Asp359 and Asn450, or via π-π stacking and non-polar interactions with the aromatic residues, or both. Some fragments showed additional polar interactions with side chains or the backbone located at the edge of the pocket (Fig. 2a, d, g, n, o). However, the vast majority of the bound fragments consisted of at least one aromatic heterocycle, which is anchored to either Asp359 or Asn450 via a polar interaction plus an additional π-π stacking with either Tyr332 at sub-site 1a or Trp385 at sub-site 1b. A compelling illustration of this was the binding of two molecules within the pocket, which were sandwiched between Tyr332 and Trp385 (Fig. 2a–g).

The pocket itself appeared to be comparably rigid and showed only a certain degree of plasticity with minor conformational changes of surrounding amino acids induced upon fragment binding. This was expected based on the comparison of the previously reported apo TRIM21 structure (PDB: 2VOK^2^) with the Ig2a-bound complex (PDB: 3ZO0^2^), for which only minor structural changes have been observed. Notable exceptions were fragments such as Z274575916 (Fig. 2e) or Z1401276297 (Fig. 2h), which would have clashed with Asp359 and therefore pushed the central side chain into an alternative conformation to accommodate these sterically more demanding ligands. The largest conformational changes were observed in the structure with fragment Z29634868 (Fig. 2i), in which the side chain of Trp385 was attracted towards the fragments from its position in the apo structure, forming a more favorable π-π stacking interaction with the pyrimidine core of the ligand.

In contrast, Z106579662 (Fig. 2m) shows no π-stacking and bound similarly to HEPES, forming a polar interaction with Asp359. Notably, this binding mode resembled that of a recently published acepromazine derivative (Fig. S5). However, this human TRIM21 structure was crystallized with an active site mutant (Asp355A), which exhibited much higher affinity compared to the wild type^6^.

### Site #2: secondary antibody binding pocket

**Fig. 3:**
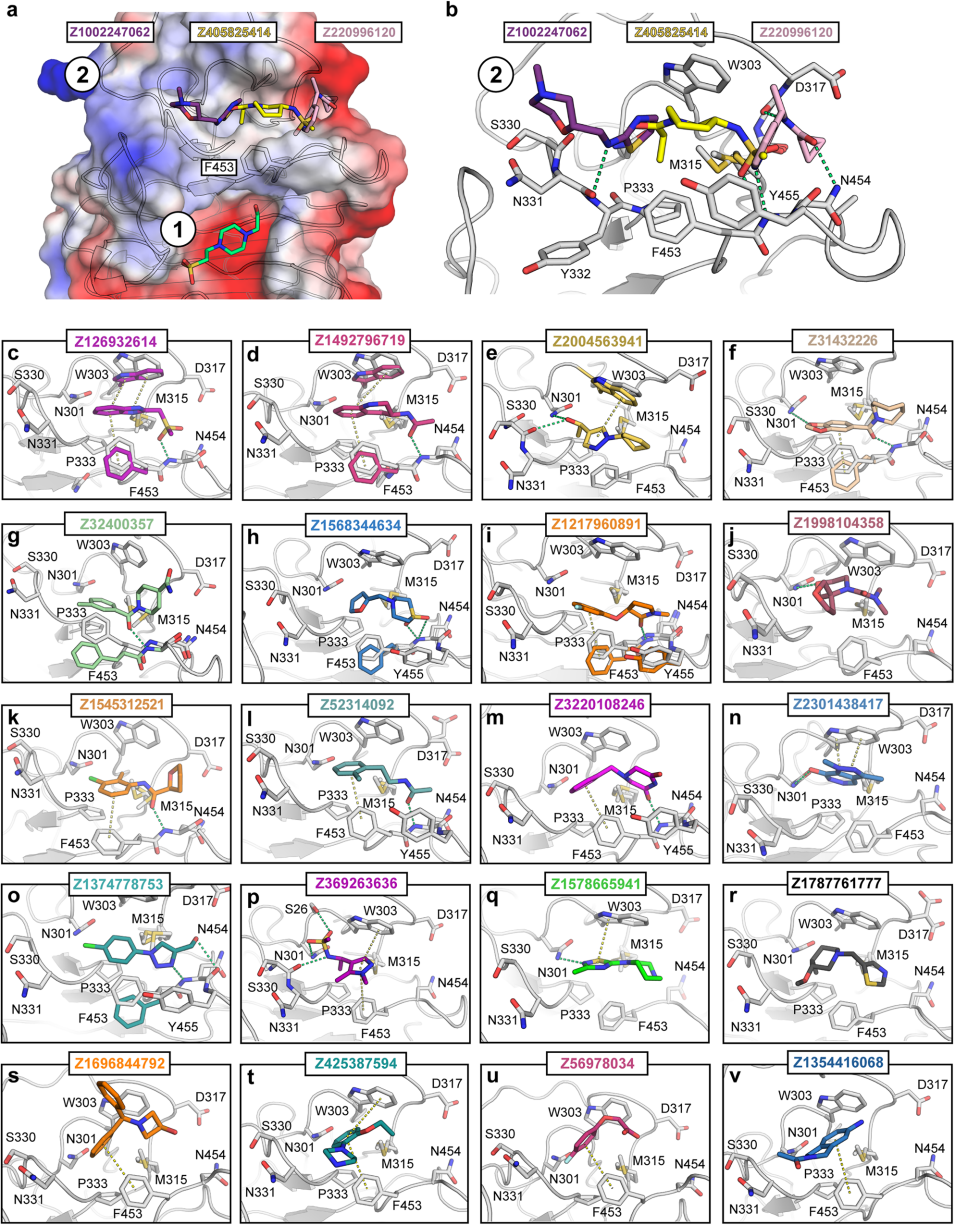
Selected fragment hits targeting site #2. Panel **a**, **b** show three fragments hits positioned next to each other as electrostatic surface representation and as a close-up, respectively. Panels **c**–**v** display selected fragment hits, highlighting the diversity of binding modes observed. Polar interactions are depicted as green, π-π interactions as yellow dashed lines. Colored side chains indicate conformational changes between the unbound and bound states, whereas gray represents the unbound state.

Site #2 is in close proximity to the primary binding site, with only Phe453 separating the two binding pockets. Similar to the site #1, the amino acids forming the pocket are highly conserved between human and murine TRIM21 (Fig. S1), with minor exceptions such as the hydrophobic methionine being replaced by the hydrophobic leucine. As revealed by the Ig2a bound complex, site #2 is also involved in antibody binding, although to a much lesser extent in comparison to site #1 (Fig. S3). The pocket is shaped to form a narrow cleft and half of the identified fragment hits (56 out of 111) bound to this cavity. The pocket consists predominantly of hydrophobic and polar amino acids with an overall neutral to slightly positive surface charge (Fig. S4). The pocket is comparatively long as shown in Fig. 3 (panels a and b), which revealed that the crevice was long enough to accommodate three fragments side to side to each other with minimal overlap. However, the vast majority of the compounds were bound in the center of the pocket between Trp303 and Phe453 (Fig. 3c–v). Many, though not all, of the bound fragments contained an aromatic heterocycle and interacted via either π- π stacking with Trp303 and/or an end on π-π stacking with Phe453 (Fig. 3c, d, e, f, i, k, l, m, n, q, s, t, u, v). Comparing all amino acid side chain conformations within this pocket revealed that Phe453 appeared to be the most flexible residue. Depending on the ligand bound, the Phe453 side chain seemed to adjust its conformation to either facilitate end-on π stacking (Fig. 3c, d, f, i) or by moving outward when sterically bulkier molecules were bound (Fig. 3g, h, o). Trp303 adopted a different conformation as well, but strikingly in comparison with the apo structure, this side chain exclusively moved towards ligands, possibly to form more favorable π stacking interactions (Fig. 3c, d, e). Many ligands formed additional polar interactions with the backbone nitrogen of Asn454 (Fig. 3b–d, f–i, l, m, o) on one side and/or with the sidechain of Asn301 on the other side. However, there were also some exceptions to these binding modes where no direct polar interaction or π stacking was observed and the fragment was bound solely by non-polar interactions.

### Site #3: small pocket

**Fig. 4:**
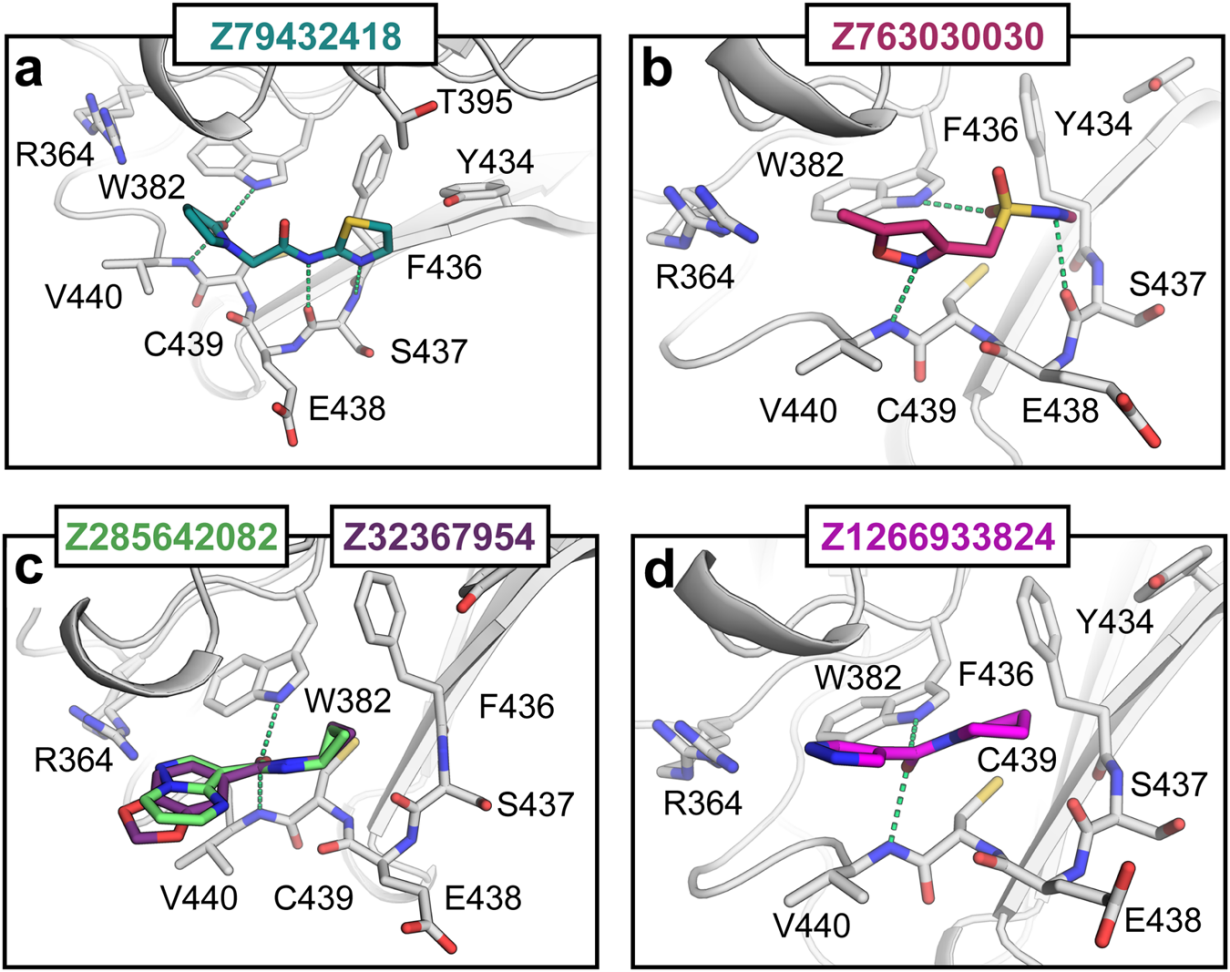
Binding modes of selected fragment hits binding to site 3. Polar interactions are depicted as green dashed lines. All fragments showed highly similar binding modes (panels **a**–**d**).

Site #3 represents a smaller binding pocket located ~18 Å from the main binding site and has not been described so far. This pocket is notably rigid, exhibiting minimal movement of the backbone or side chains upon ligand binding. All eleven fragments identified in site #3 bound in a highly similar fashion, forming hydrogen bonds with the side chain of Trp382 and the backbone nitrogen of Val440. Only two fragments made additional polar interactions with the backbone carbonyl groups of Glu438 and Ser437 (Fig. 4a, b). Interestingly, although this pocket is not involved in antibody binding, the sequence homology between murine and human protein is highly conserved in this area, with the only difference being the substitution of valine in mouse with alanine in human TRIM21 (Fig. S1). It is worth mentioning that the presence of a cysteine residue (Cys439) provides an opportunity to target this pocket covalently.

### Site #4. Pocket/interface binding site

**Fig. 5:**
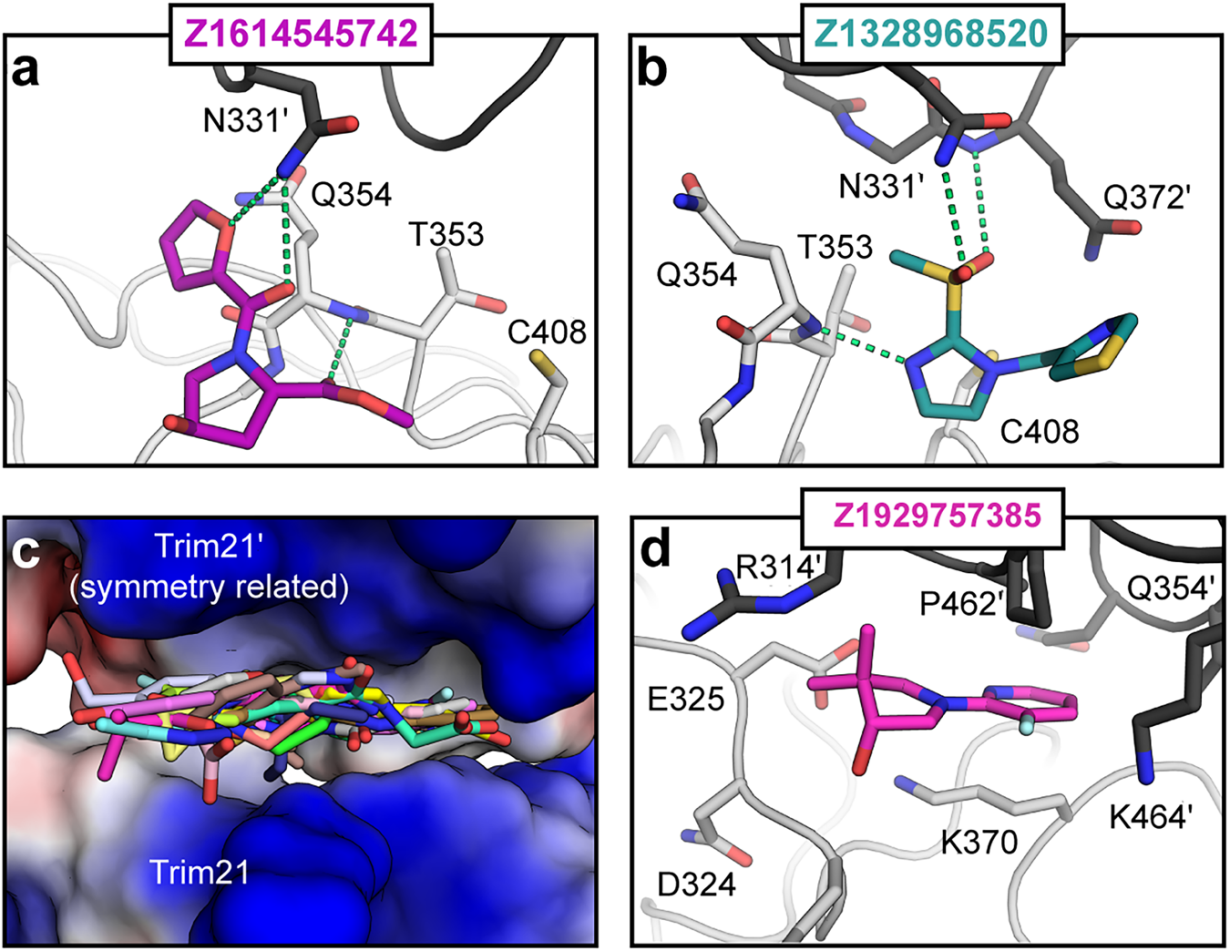
Fragment hits targeting site #4 and site #5. Fragment hits located in site #4 (**a**, **b**) and site #5 (**c**, **d**). Polar interactions are depicted as dashed green lines. TRIM21 is shown in gray, the symmetry related TRIM21 molecule in black, except **c** which shows the surface charge representation for both.

Only two fragments bound to site #4. Although both fragments occupied this uncharacterized pocket, they also interacted notably with a symmetry-related molecule, leading us to consider this site at least partially as a crystal interface binding site. Both ligands shared a similar binding mode, forming hydrogen bonds with the backbone nitrogen of Gln372 and the side chain of Asn331 located in the symmetry-related molecule (Fig. 5a, b). Both the interacting amino acids is conserved between mouse and human TRIM21 and therefore the observed binding might be specific to the mouse orthologue.

### Site #5: crystal interface binding site

The remaining 25 fragments bound to site #5 appeared to be surface-attached at first glance, rather than occupying a cavity. Further inspection revealed that these fragments primarily bound to a neutral-to-positively charged pocket formed by the crystal interface (Fig. S4), suggesting that this site is only present in the context of the crystal (Fig. 5c, d). Similar to site #4, the sequence identity between murine and human TRIM21 is not conserved in this region, and therefore we consider this site to be of limited interest for further elaboration.

### Hit validation

Fragment-based drug discovery involves working with small molecules that typically have low affinities for their target proteins, often in the high micromolar to millimolar range. This presents a significant challenge in confirming binding of initial hits from fragment screens using biophysical methods^22,23^. While high-affinity binding is not expected at this stage, we sought to validate our fragment hits by using two complementary techniques. Initially, our primary methods were surface plasmon resonance (SPR) and differential scanning fluorimetry (DSF).

We purchased and tested all compounds from sites #1, #2, and #3 on both human and mouse TRIM21 PRY-SPRY domains, excluding hits from sites #4 and #5 due to the reasons mentioned above. Both methods confirmed a small subset of hits, but as anticipated, not all hits could be validated, likely due to very weak binding affinities beyond the detection limits of the respective techniques.

Due to the expected weak binding of the identified fragments, SPR experiments required a high compound concentration (500 µM). We initially screened the fragments at a single dose, using suramin (PDB: 9GTE) as a control compound as it has been shown to bind to both species with an affinity of ~10 µM^24^. To ensure protein stability on the chip surface and account for drift effects, suramin was reinjected at 50 µM after every 15th compound injection.

In the following analysis, we focused on compounds exhibiting a response signal greater than the suramin response of 100 relative RU. Compounds with signals below this reference threshold were considered too weak and were excluded from further analysis.

**Fig. 6:**
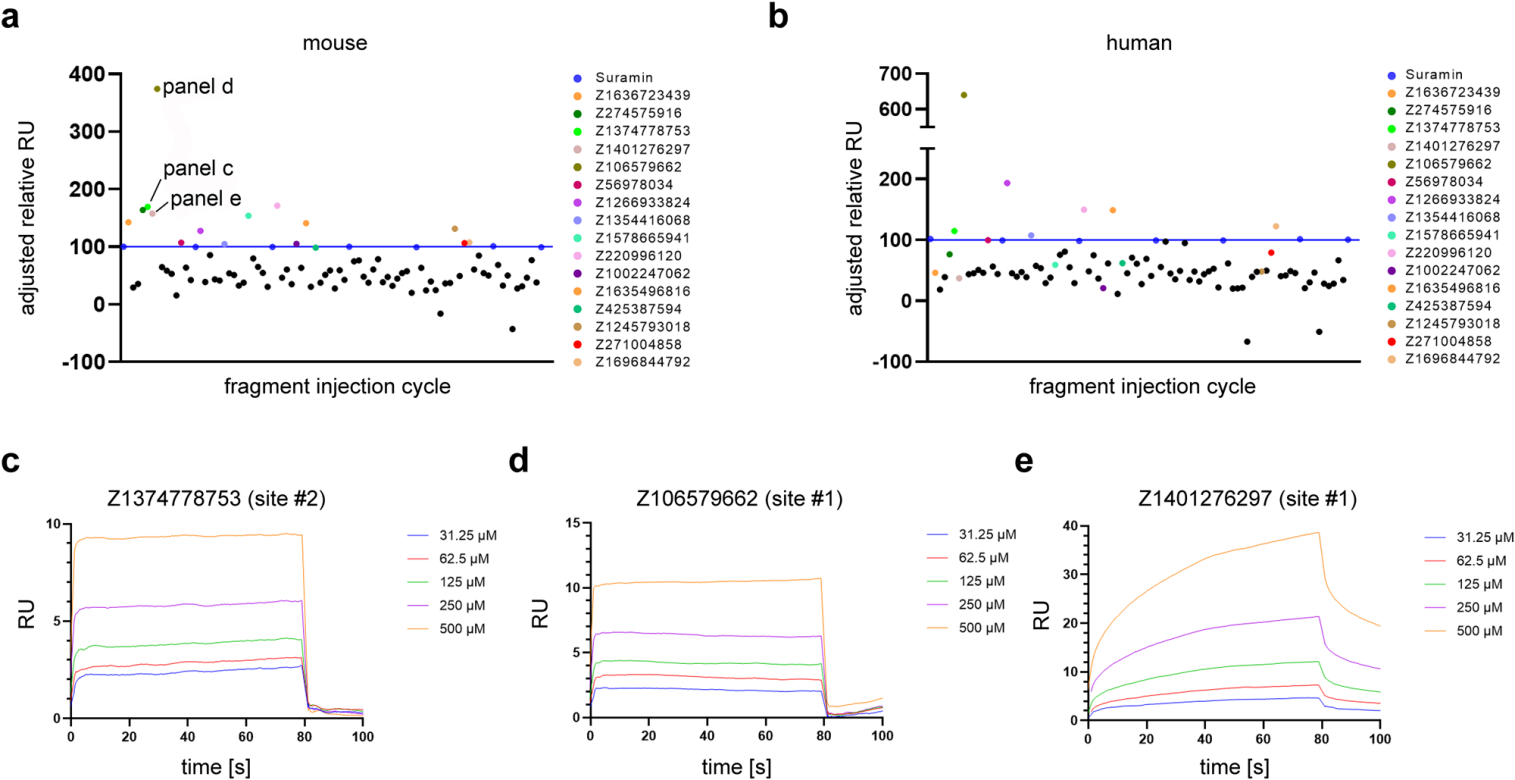
SPR screening results. SPR screening of fragments binding to site #1, #2, and #3 in mouse (**a**) and human TRIM21 PRY-SPRY domain (**b**). Fragments that yielded a higher relative response compared to reference Suramin in mouse protein (blue line and dots) were highlighted by different colors. The colors of the fragments match the colors used in other figures. **c**, **d**, and **e** show dose response data for three most significant relative response signals for mouse TRIM21 PRY-SPRY domain.

Notably, with a few exceptions (Z1636723439, Z274575916, Z1578665941, and Z1245793018), we observed comparable binding profiles for the same fragments for both human and mouse TRIM21 PRY-SPRY. Of the 16 compounds highlighted in Fig. 6, seven bound to site #1, eight bound to site #2, and one bound to site #3.

We then performed a dose-response analysis for the compounds which showed the highest relative response for the mouse protein (Fig. 6c–e). While the sensorgram appeared typical for a weak ligand, curve fitting to a steady-state binding model proved challenging. Saturation of the target surface with fragments was not achieved, likely due to nonspecific interactions with the chip and/or protein surface, particularly at higher concentrations (Fig. 6c, d). For other fragments, the response signal did not reach a stable plateau (Fig. 6e). However, as fragments may have binding affinities in the high micromolar to millimolar range, it may not be practically feasible to saturate the protein surface with the fragments. A further complication that limits high concentration screening is the common observation that many fragments may aggregate and bind non-specifically at high concentrations^25^. Consequently, we have chosen not to report K_D_ values, as such data would not be accurate and probably overfitting the data. Instead, the results should be interpreted as binding conformations of the crystallized fragments with affinities likely in the high micromolar to low millimolar K_D_ range.

The DSF (ΔT_m_) assay presented challenges as well, due to the small temperature shifts expected for weak ligands. We tried to overcome this obstacle by using a higher compound concentration (2 mM). However, the high concentration came with the cost of higher auto fluorescence that we observed for two compounds which we excluded from the analysis. Despite these difficulties, we were able to identify five compounds with temperature shifts larger than 0.2 °C for mouse and eight for human TRIM21. The largest shifts we observed for compound Z2065616520 (0.4 °C mouse and 0.6 °C human), Z1401276297 (0.2 °C mouse and 0.6 °C human), Z106579662 (0.2 °C mouse and 0.6 °C human).

Overall, the DSF assay identified a similar set of compounds for both species, and most compounds that gave rise to positive ΔT_m_ shifts were the ones previously confirmed by SPR as ligands with an above-average relative SPR response, suggesting a good correlation between the two orthogonal methods. However, similar to the SPR data, the results should be treated with caution due to the relatively modest temperature shifts observed. For comparison, suramin binding resulted in a ΔT_m_ shift of 2.3 °C at the screening concentration. However, DSF shifts should be interpreted as relative values, as different proteins and binding sites may show different ΔT_m_ shifts for ligands with similar ligand affinity. Detailed information for each compound, including specific SPR response, DSF results, binding site, compound ID, chemical structure, and PDB codes, are summarized in Table S1.

**Fig. 7:**
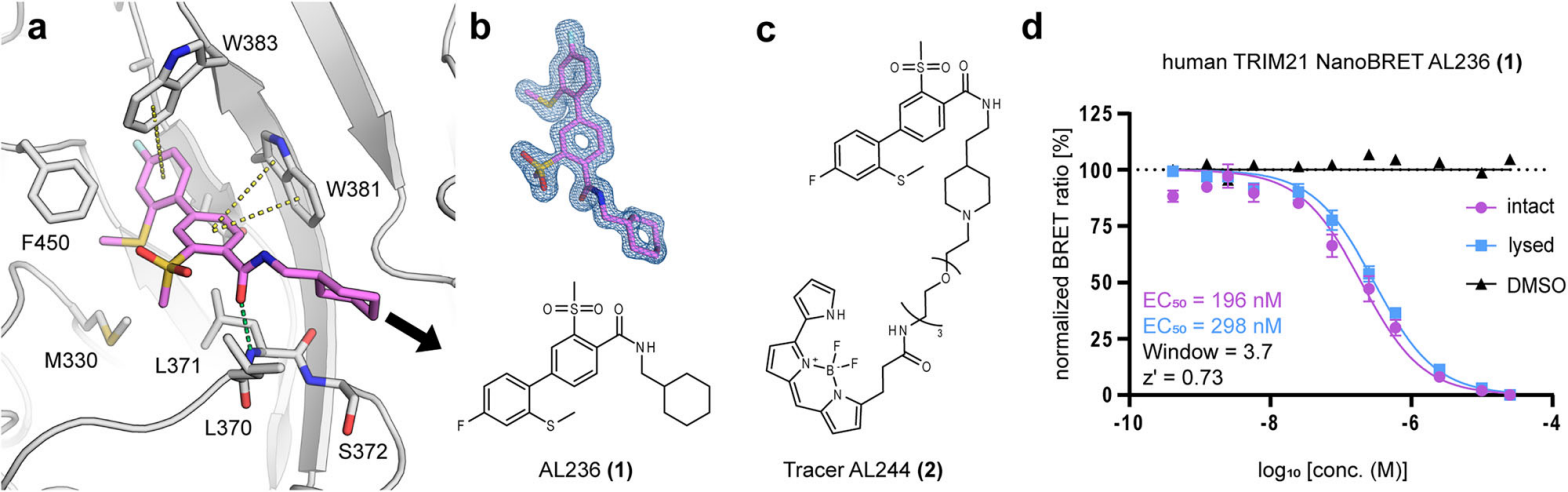
Crystal structure of AL236 **(1)** and NanoBRET results on human TRIM21. **a** AL236 **(1)** bound to human TRIM21 PRY-SPRY domain (PDB 9QBA). Polar interactions are depicted as green, π-π interactions as yellow dashed lines, respectively. The arrow indicates the exit vector for linker attachment. **b** 2F_o_-F_c_ electron density map contoured at 1.5 sigma and chemical structure for AL236 **(1)**. **c** Structural formula of tracer AL244 **(2)**. **d** NanoBRET EC_50_ determination of AL236 **(1)** in intact and lysed cells assay format. Error bars indicate the standard deviation (SD) of triplicate measurements.

**Fig. 8:**
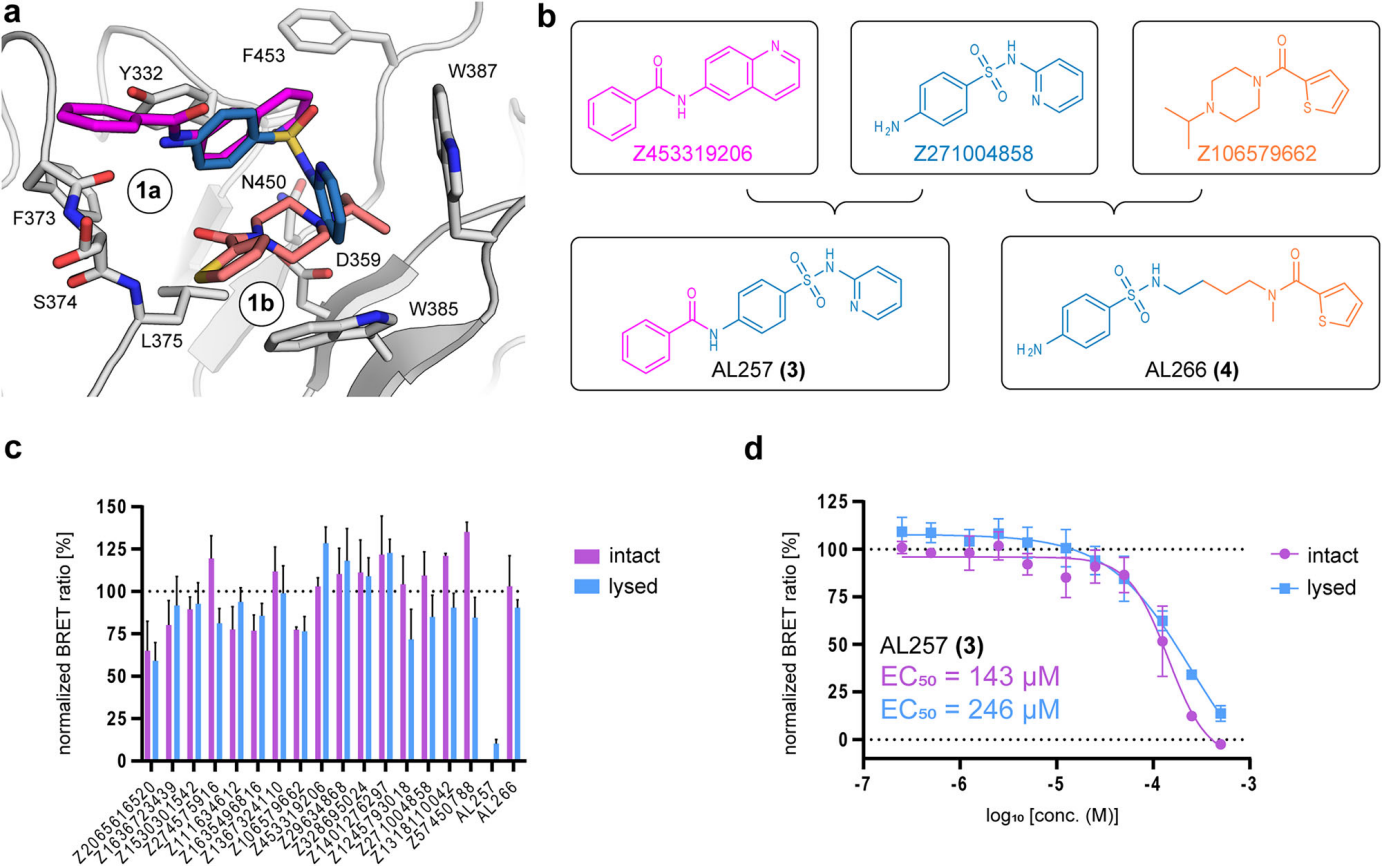
Follow-up compound design and evaluation. **a** Superimposition of fragments used for merging: the sub-sites are indicated. **b** Structural formulas of fragments and synthesized follow up compounds. **c** Tracer displacement at a concentration of 500 µM. Purple bars represent data measured in intact cells, while blue bars represent data measured in lysed cells (digitonin) determined by NanoBRET. **d** Dose response of AL257 **(3)** in intact and lysed cells demonstrate effective cell penetration of these merged fragments. Error bars indicate the standard deviation (SD) of triplicate measurements. Additional information can be found in Supplementary Fig. S6.

While this paper was under revision, Li et al. published a preprint describing a series of high-affinity ligands identified by optimizing a DEL (DNA-encoded library) screening hit, with low nanomolar potency for human TRIM21 PRY-SPRY domain^26^. Based on the discovered scaffold, we synthesized the representative ligand AL236 (1) and crystallized it under similar conditions as previously reported (Fig. 7a). Using the resulting high-resolution structure (PDB: 9QBA, 1.45 Å) in complex with human TRIM21, we rationally designed a BODIPY-based fluorescent tracer for use in a NanoBRET assay. The structure revealed that the ligand is occupying the whole pocket and binds by a combination of polar and nonpolar interactions. Similar to many fragments, it forms π-π interactions with the two tryptophans Trp381 and Trp383, as well as a polar interaction via its amide to the backbone of Leu371. Further structural analysis indicated that a promising strategy for an exit vector is to replace the terminal cyclohexyl group with a piperidine moiety (Fig. 7a) and conjugate the BODIPY dye via a PEG linker to the piperidine nitrogen (Fig. 7c). This resulted in the tracer AL244 **(2)**, which demonstrated desired properties. Tracer validation experiments showed a robust BRET signal and assay window in combination with a C-terminally fused NanoLuc to full-length TRIM21. In contrast, an N-terminally fused NanoLuc resulted in a significantly weaker BRET signal (Fig. S6). Initial measurements in life cells (intact mode) showed that AL236 **(1)** effectively displaced the tracer, with an EC_50_ of 197 nM. Under permeabilized conditions (lysed mode), we obtained a similar result (EC_50_: 297 nM), indicating a high cell permeability of this compound. These confirmed the findings of Li et al.^26^. Next we assessed the binding of fragments targeting site 1. As expected, none of the fragments displace the tracer completely at a concentration of 500 µM. (Fig. 8c). Nevertheless, some of the fragments showed significant partial displacement of the tracer albeit to low displacement levels (~20%). The most potent fragment was Z2065616520, which showed ~40% tracer displacement, followed by Z106579662 with ~25% displacement. Both fragments also showed an above-average response in the SPR and ΔT_m_ assay.

### Fragment merging

With the established assay format, our next objective was to perform a proof-of-concept fragment-merging follow-up targeting site #1. To guide this approach, we performed a structural superposition of all identified fragments targeting site #1, leading to the selection of Z271004858 as the core scaffold, due to its central positioning within the binding pocket (Fig. 8a). This fragment served as an optimal anchor for merging additional fragments that bound specifically to sub-sites #1a and #1b. Z453319206 in particular showed a vast structural overlap with Z271004858 and was selected to elongate the core scaffold on sub-site #1a to create compound AL257 **(3)**. For sub site #1b we choose fragment Z106579662 for merging, due its above average response in SPR, ΔT_m,_ and NanoBRET yielding AL266 **(4)** (Fig. 8b). Subsequently both merge fragments were tested using our developed NanoBRET assay. While AL266 **(4)** showed no activity at 500 µM, AL257 **(3)** displaced the tracer almost completely. The follow up dose response experiment determined an EC_50_ of 143 µM in intact and 246 µM in lysed cells (Fig. 8d), which demonstrates the potential of fragment merging strategies to enhance activity using our large fragment data set.

## Discussion

In this study, we performed a CFS on the PRY-SPRY domain of mouse TRIM21, which led to the identification of 109 fragments. Given the high sequence identity between human and mouse protein, we assume that these results are directly transferable to human TRIM21, which was further underlined by our DSF and SPR results that confirmed interaction for similar fragments for both proteins. In total, we identified five distinct binding sites within the PRY-SPRY domain, including two sites which are directly involved in the antibody binding (site #1 & site #2) and one additional site which has not been previously described (site #3). The remaining two binding sites (site #4 & site #5) appeared to be rather crystallization artefacts than actual binding sites. Furthermore, in comparison with sites #1–3, the sequence identity with its human counterpart is not conserved in this region and thus of limited interest for further investigation.

The primary binding pocket (site #1) has shown an intriguing capacity to accommodate two molecules simultaneously. This observation suggests the potential for cooperative binding between fragments from two molecules or the design of larger, more potent ligands by linking fragments that fully occupy the pocket and make optimal interactions with surrounding residues. Most fragments were anchored by polar interactions, along with additional π-stacking or hydrophobic interactions, similar to those observed in antibody binding. Exploiting these interactions, we synthesized AL257 **(3)** by merging two fragments, which exhibited enhanced activity against human TRIM21 compared to the parent fragment molecules. This result highlights the potential of fragment-based ligand design for developing TRIM21 inhibitors using the provided structural data.

In the secondary binding pocket (site #2), the X-ray fragment screening revealed a total number of 56 fragments, indicating significant potential for fragment merging. The pocket itself is notably elongated, with fragments occupying diverse positions along the crevice. Many of the fragments showed considerable overlap in atom positioning, suggesting opportunities for structure aided fragment merging to optimize binding and enhance compound potency. Giving recent publication and preprints that identified diverse scaffold acting as molecular glue to degrade nuclear pore proteins, site #2 might emerge as an alternative to targeting site #1, if the degradation activity induced by these ligands on identified neo-substrates cannot be eliminated during PROTAC design^26–28^.

Site #3 has not been described previously and presents a different targeting opportunity for TRIM21. Compared to sites #1 and #2, the site#3 cavity is smaller, more rigid, and accommodated fewer chemically diverse fragments. Nevertheless, it offers an attractive alternative binding site for targeting TRIM21 without interfering with its natural substrate recruitment or cellular E3 ligase function. The presence of a central cysteine further offers the possibility of covalent targeting using reactive warheads, such as acrylamides, commonly used in covalent inhibitor development. Additionally, PROTACs or other bivalent molecules targeting this site could synergize with compounds engaging the primary or secondary pockets.

Furthermore, we determined a high-resolution crystal structure of the potent ligand AL236 **(1)** bound to human TRIM21, which guided the design of AL244 **(2)**, a tracer for a NanoBRET assay. This assay enabled the rapid evaluation of compound potency using full-length TRIM21 and live-cell target engagement, providing a valuable tool for future drug discovery efforts.

Taken together, our study presents high-resolution structures, identifies key binding pockets, and elucidates diverse fragment binding modes within TRIM21. These findings provide a solid foundation for structure-guided drug design. Follow-up work will focus on optimizing these initial fragments through merging and optimizing hydrogen bonding and π-stacking interactions, ultimately leading to the development of structurally diverse and potent TRIM21 inhibitors.

## Material and methods

### Chemical synthesis

A detailed description of the chemical synthesis, along with analytical data, can be found in the Supplementary Information under section 8.

### Plasmids

Information on the vectors and protein sequences used for experiments can be found in the Supplementary Information under section 9.

### Expression and purification

The T7 expression plasmid (pET-3d) of the mouse TRIM21 PRY-SPRY domain (residues V291-M470, UniProt-ID: Q62191) with an N-terminal His_6_-tag was transformed in BL21(DE3) cells. Bacteria were grown in Terrific Broth medium containing 100 mg mL^−1^ ampicillin (6 L). Protein expression was induced at an OD_600_ of 2 by using 0.5 mM isopropyl-β-D-thiogalaktopyranosid (IPTG) at 18 °C for 12 h. Cells were lysed by sonication in lysis buffer containing 50 mM Tris(hydroxymethyl)aminomethan (Tris) pH 7.8, 500 mM NaCl, 5% (v/v) glycerol, and 1 mM tris-(2-carboxyethyl)-phosphin (TCEP). After centrifugation, the supernatant was loaded onto a Nickel-Sepharose column equilibrated with 50 mL lysis buffer. The column was washed with 60 mL lysis buffer. Proteins were eluted by an imidazole step gradient (50, 100, 200, 300 mM). Fractions containing protein were pooled together and loaded onto a Superdex 75 16/600 Hi-Load gel filtration column equilibrated with size-exclusion chromatography (SEC) buffer (25 mM Tris pH 7.8 and 150 mM NaCl). The purity of the protein was assessed by sodium dodecyl sulfate polyacrylamide gel (SDS-PAGE) and pure protein fractions were concentrated to approximately 10 mg mL^−1^.

Human TRIM21 PRY-SPRY domain (residues V287-I465, UniProt-ID: P19474) with an N-terminal His_6_-tag followed by a SUMO tag (pSUMO-LIC vector) site was transformed in BL21(DE3) cells. Bacteria were grown in Terrific Broth medium containing 50 mg mL^−1^ kanamycin (6 L). Protein expression was induced at an OD_600_ of 2 by using 0.5 mM IPTG at 18 °C for 12 h. Cells were lysed by sonication in lysis buffer containing 50 mM 3-(N-morpholino)propansulfonsäure (MOPS pH 6.9, 800 mM NaCl, 10 mM imidazole, 1 mM TCEP. After centrifugation, the supernatant was loaded onto a nickel-sepharose column equilibrated with 30 mL of lysis buffer. The column was washed with 60 mL of lysis buffer. Proteins were eluted using an imidazole step gradient (50, 100, 200, 300 mM). Fractions containing the protein were pooled, and the tag was cleaved by adding SUMO protease and dialyzing overnight against SEC buffer (25 mM MOPS, pH 6.9; 250 mM NaCl, 0.5 mM TCEP). The next day, a second nickel-sepharose column was used to remove uncleaved protein and the tag. The flow-through was collected, concentrated to approximately 5 mL, and loaded onto a Superdex 75 16/600 Hi-Load gel filtration column equilibrated with SEC buffer. The purity of the protein was assessed by SDS-PAGE, and pure protein fractions were concentrated to approximately 10 mg mL^−1^.

### Differential scanning fluorimetry (DSF)

Briefly, recombinant murine and human TRIM21 PRY-SPRY domain at a concentration of 8 μM were mixed with 2 mM compound in their respective SEC buffer. SYPRO Orange (5000×, Invitrogen) was added as a fluorescence probe (1 µl per mL). Subsequently, temperature-dependent protein unfolding profiles were measured using the QuantStudio™ 5 realtime PCR machine (Thermo Fisher). Excitation and emission filters were set to 465 nm and 590 nm, respectively. The temperature was raised with a step rate of 3 °C per minute. Data points were analysed with the internal software (Thermal Shift SoftwareTM Version 1.4, Thermo Fisher) using the Boltzmann equation to determine the inflection point of the transition curve.

### Surface plasmon resonance experiments

Surface plasmon resonance (SPR) experiments were performed using a Biacore T200 instrument at 25 °C. His-mTRIM21 PRYSPRY and His-SUMO-hTRIM21 PRY-SPRY were immobilized onto flow channels (FC) FC2, FC3, and FC4 of a Series S sensor chip NTA (Cytiva). Briefly, 500 µM NiCl_2_ was injected to load the surface with nickel, followed by the injection of a 1:1 (v/v) mixture of 483 mM EDC and 10 mM NHS to activate the surface for covalent coupling. Proteins (10–20 µg mL^−1^ in HBS-P+) were injected at 10 µL min^−1^ to achieve response levels of 3500–4300 RU for mTRIM21 and 3600–4000 RU for hTRIM21. Flow channel 1 (FC1) was used as a reference surface. Surface deactivation and nickel stripping were achieved using 1 M ethanolamine and 350 mM ethylenediaminetetraacetic acid (EDTA), respectively.

Fragment screening was conducted by injecting 500 µM fragments diluted in running buffer (HBS-P+ with 1 mM TCEP and 2% (v/v) dimethyl sulfoxide (DMSO)) over all flow-channels for 30 s at 30 µL/min^−1^. To account for surface drift, 50 µM of a control compound (Suramin) was injected every 15 fragment injections, and blank injections were performed every 5 fragments. Responses were analyzed by blank subtraction, reference subtraction (FC1), solvent correction, molecular weight adjustment, and drift correction using Biacore T200 Evaluation Software v3.2.1. The binding threshold for fragment hits was set based on the Suramin-binding response level.

For dose response analysis, 5 fragment concentrations of 31.25–500 µM were injected with an association time of 80 s and dissociation time of 150 s at 30 µL min^−1^. The data was solvent-corrected, reference-subtracted (FC1), and blank-subtracted using the T200 Evaluation Software v3.2.1. Sensorgrams were subsequently replotted using Graphpad v.9 software.

### NanoBRET® assay

Full-length hTRIM21 with N- or C-terminal NanoLuc fusion was transfected into HEK293T cells (ATCC) using Fugene 4K (Promega, Cat. No. E5911) transfection reagent according to the manufacturer’s instructions. Following protein expression for 24 h, cells were split again in OptiMEM medium (phenol red free, Life Technologies) to a final amount of 2 × 10^5^ cells per mL and 10 µL per well were distributed in a 384 well plate. Next, tracer AL244 **(2)** was added in concentrations from 3 µM to 6 nM. After 2 h of equilibration at 37 °C and 5% CO_2_, NanoBRET® NanoGlo substrate (Promega) was added as described in the manufacturer’s protocol and after 5 min, luminescence was measured on a PHERAstar plate reader (BMG Labtech) equipped with a luminescence filter pair [450 nm band-pass filter (donor) and 610 nm long-pass filter (acceptor)]. Then, cells were permeabilized through the addition of 25 nL Digitonin (0.05 µg µL^−1^). Following another 5-min incubation, filtered luminescence was measured again. The data acquired in the tracer titration was converted into mBRET units (Fluorescence intensity at 610 nm / Luminescence intensity at 450 nm) × 1000 and graphed using Graph-Pad Prism v.9 software.

### Competition binding experiments

A 384-well plate with cells expressing hTRIM21-NLuc was prepared as described above. Three micrometers of Tracer AL244 **(2)** was added and either fragments or derivatives targeting site 1 and follow up compounds AL257 **(3)** and AL266 **(1)** were added to the cells at a concentration of 500 µM using an Echo 550 acoustic dispenser (Labcyte). Additionally, AL236 **(1)** was used as a positive control and added in concentrations ranging from 25 µM to 0.4 nM. After 2 h of equilibration at 37 C and 5% CO_2_, NanoBRET® NanoGlo substrate (Promega) was added as described in the manufacturer’s protocol and filtered luminescence was measured after a 5-min incubation time. Then, cells were permeabilized with Digitonin and, following 5 min of incubation, filtered luminescence was measured. The data acquired in the competition experiments were converted to mBRET units, then normalized to the positive and negative control and graphed using Graph-Pad Prism v.9 software using a normalized three-parameter curve fit using the following equation: y = 100/[1 + 10(x − logIC_50_)].

### X-ray crystallography

Purified mouse TRIM21 PRY-SPRY domain at 10 mg mL^−1^ in SEC buffer was crystallized using the sitting-drop vapor diffusion method, with a reservoir solution containing 4% (v/v) PEG 400, 2 M ammonium sulfate, and 0.1 M 4-(2-hydroxyethyl)-1-piperazineethanesulfonic acid (HEPES) pH 8.0. Crystals of TRIM21 in the space group I4 with a single monomer in the asymmetric unit, were grown with drop ratios of 0.2 μL protein, 0.2 μL reservoir solution at 20 °C.

Fragments were soaked into crystals by adding compound stock solutions directly to the crystallization drops using an ECHO liquid handler^20,29^. In brief, fragments of ethylene glycol solutions were transferred directly to crystallization drops, giving a final compound concentration of 10 mM and an ethylene glycol concentration of 10% (v/v). Drops were incubated at 4°C for 2 h before mounting and flash cooling in liquid nitrogen without the addition of further cryoprotectant^30^.

Human TRIM21 PRY-SPRY domain at 10 mg mL^−1^ was co-crystallized with 500 µM AL236 **(1)** using the sitting-drop vapor diffusion method, with a reservoir solution containing 2.3 M sodium chloride and 0.1 M sodium citrate pH 3.5. Crystals in the space group P6_2_ with a single monomer in the asymmetric unit, were grown with drop ratios of 0.1 μL protein, 0.1 μL reservoir solution at 20 °C. Before flash freezing in liquid nitrogen, the crystals were cryoprotected with 25% ethylene glycol dissolved in reservoir solution.

Data were collected at diamond light source (DLS) beamline I04 (mouse TRIM21) or I03 (human TRIM21) at 100 K and processed with the fully automated pipelines at Diamond, which include XDS^31^, xia2^32^, autoPROC^33^, and DIALS^34^. Further analysis was performed using XChemExplorer^35^, with electron density maps generated using DIMPLE. Ligand binding events were identified using PanDDA2 (https://github.com/ConorFWild/pandda_2_gemmi). After initial placement of the ligand, identified hits were inspected manually in Coot^36^ to assess the presence or absence of the expected ligand in the calculated event and/or electron density maps. Subsequent model building and refinement was carried out manually in Coot with iterative refinement cycles until convergence was reached in Buster [Buster v. 2.10.13 (Cambridge, UK, 2017)] or Refmac5^37^. Ligand restraints were calculated with AceDRG^38^ or GRADE [grade v. 1.2.19 (Global Phasing Ltd, Cambridge, UK, 2010)], and quality annotations were reviewed using XChemReview, Buster-Report [Buster v. 2.10.13 (Cambridge, UK, 2017)], and Mogul^39,40^. The coordinates and structure factors have been deposited in the Protein Data Bank (PDB) under the group deposition ID G_1002320. The human TRIM21 structure in complex with AL236 **(1)** was built and refined individually and deposited under PDB ID: 9QBA. Detailed data collection and refinement table can be found in Supplementary Information Table S2.

## Supplementary information

Supplement Information

nr-reporting-summary.

## Data Availability

Tracer information will be uploaded to TracerDB (https://www.tracerdb.org/)^41^ under ID T000075. A detailed data collection and refinement table can be found in Supplementary Information Table S2. All structures have been deposited and released under the PDB IDs below. Additionally, the structures and maps have been uploaded to Fragalysis (https://fragalysis.diamond.ac.uk). Apart from these publicly available sources, data are available upon reasonable request to the authors. PDB-IDs: 7HN1, 7HMH, 7HMP, 7HMG, 7HLA, 7HM7, 7HMF, 7HNP, 7HLH, 7HLP, 7HLT, 7HLD, 7HNZ, 7HMK, 7HNO, 7HN8, 7HMQ, 7HO9, 7HMV, 7HO8, 7HMU, 7HMR, 7HM4, 7HMT, 7HML, 7HN5, 7HLU, 7HNB, 7HMJ, 7HND, 7HMS, 7HMW, 7HO4, 7HM1, 7HMB, 7HNC, 7HLB, 7HNX, 7HO1, 7HN2, 7HME, 7HMD, 7HMA, 7HM9, 7HO6, 7HO3, 7HLW, 7HN0, 7HNJ, 7HNN, 7HMZ, 7HLM, 7HM2, 7HNM, 7HO0, 7HN6, 7HNF, 7HN3, 7HLI, 7HMM, 7HLE, 7HNG, 7HMI, 7HLN, 7HLC, 7HNK, 7HM0, 7HN9, 7HNW, 7HNR, 7HNE, 7HO7, 7HMX, 7HO5, 7HM3, 7HLX, 7HLQ, 7HN7, 7HLK, 7HNI, 7HLV, 7HNS, 7HLJ, 7HLL, 7HNU, 7HLF, 7HLG, 7HLO, 7HLR, 7HLS, 7HLY, 7HLZ, 7HM5, 7HM6, 7HM8, 7HMC, 7HMN, 7HMO, 7HMY, 7HN4, 7HNA, 7HNH, 7HNL, 7HNQ, 7HNT, 7HNV, 7HNY, 7HO2, 7HOA, 7HOB, 9QBA.
