# Supplementary material for: Crystallographic fragment screening reveals ligand hotspots in TRIM21 PRY-SPRY domain
Source: Commun Chem. 2025 Jun 13;8:185. doi: 10.1038/s42004-025-01574-3 (PMC12166061; doi:10.1038/s42004-025-01574-3)

Supplementary Information

Crystallographic Fragment Screening Reveals Ligand Hotspots in TRIM21 PRY-SPRY Domain

Yeojin Kim[1, 2#], Aleksandar Lučić[1, 2#], Christopher Lenz[1, 2], Frederic Farges[1, 2], Martin P. Schwalm [1, 2, 7], Krishna Saxena[1,2], Thomas Hanke[1,2], Peter G. Marples[4, 5], Jasmin C. Aschenbrenner[4 ,5], Daren Fearon[4 ,5], Frank von Delft[4, 5, 6], Andreas Krämer[1, 2, 3*], Stefan Knapp[1, 2, 3, 7*]

[1] Institute of Pharmaceutical Chemistry, Goethe University, Frankfurt am Main, Germany

[2] Structural Genomics Consortium, Buchmann Institute of Molecular Life Sciences (BMLS), Frankfurt am Main, Germany

[3] Frankfurt Cancer Institute, Goethe University, Frankfurt am Main, Germany

[4] Diamond Light Source Ltd, Harwell Science and Innovation Campus, Didcot, UK

[5] Research Complex at Harwell, Harwell Science and Innovation Campus, Didcot, UK

[6] Centre for Medicines Discovery, University of Oxford, NDM Research Building, Oxford, UK

[7] German Translational Cancer Consortium (DKTK, site Frankfurt-Mainz)

[#] These authors contributed equally

Table of Content:

1. Binding sites comparison of human and murine TRIM21 PRY-SPRY domain 2

2. Crystal package comparison of murine TRIM21 PRY-SPRY 2

3. Comparison of antibody Fc-region binding to human and murine TRIM21 3

4. Electrostatic surface potential of binding sites 3

5. Recently published human TRIM21 mutant structure (PDB: 8Y58) 4

6. NanoBRET assay validation and results 5

7. Fragments hits overview with SMILES, DSF, SPR data and PDB codes 6

8. Chemical Synthesis & Analytical data 12

9. Used plasmids and protein sequences 37

10. Data Collection and Refinement Table 38

1. Binding sites comparison of human and murine TRIM21 PRY-SPRY domain

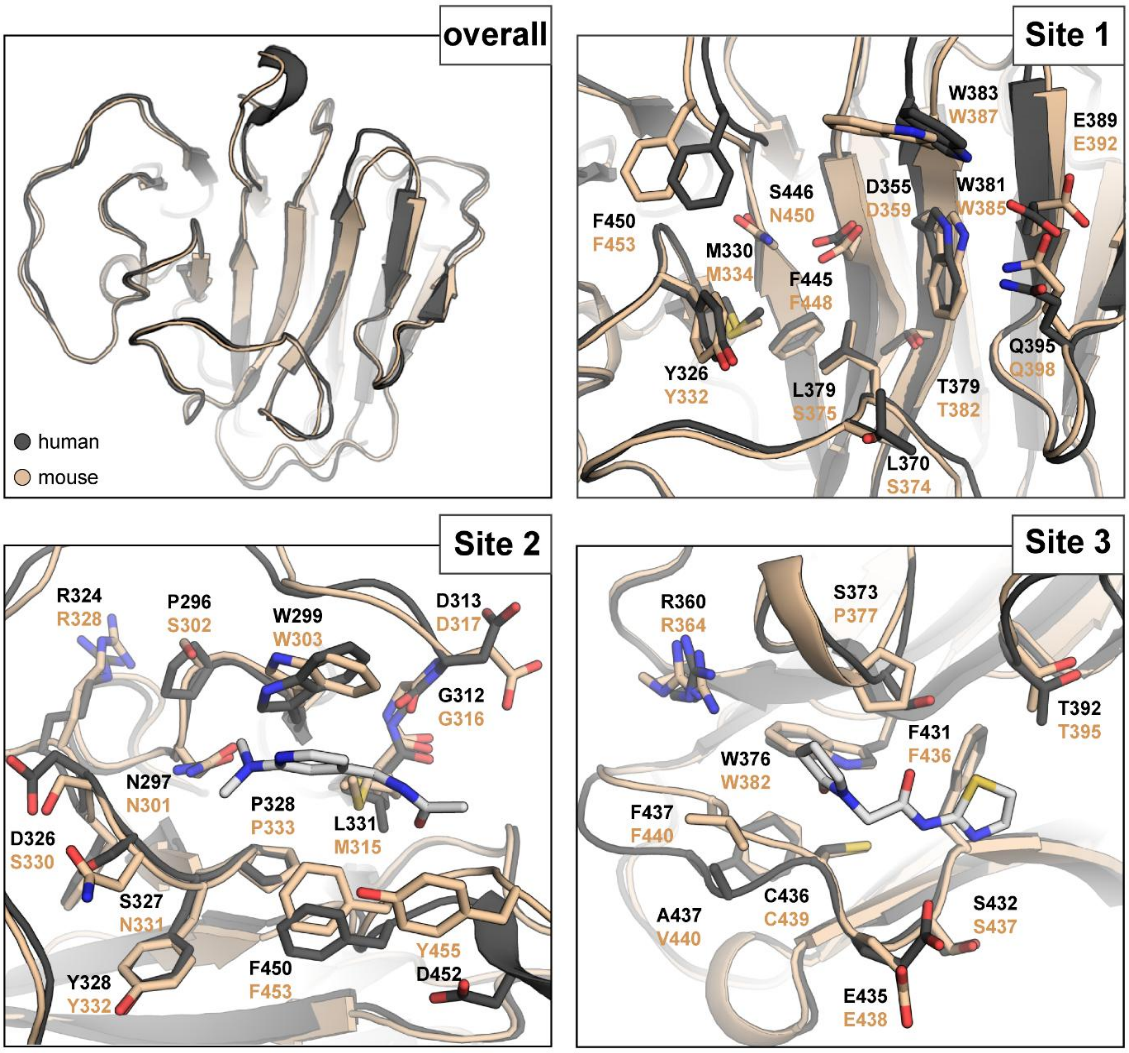

Figure S1: Overall backbone and amino acids comparison of site 1-3 between human (black) and murine TRIM21 (wheat).

2. Crystal package comparison of murine TRIM21 PRY-SPRY

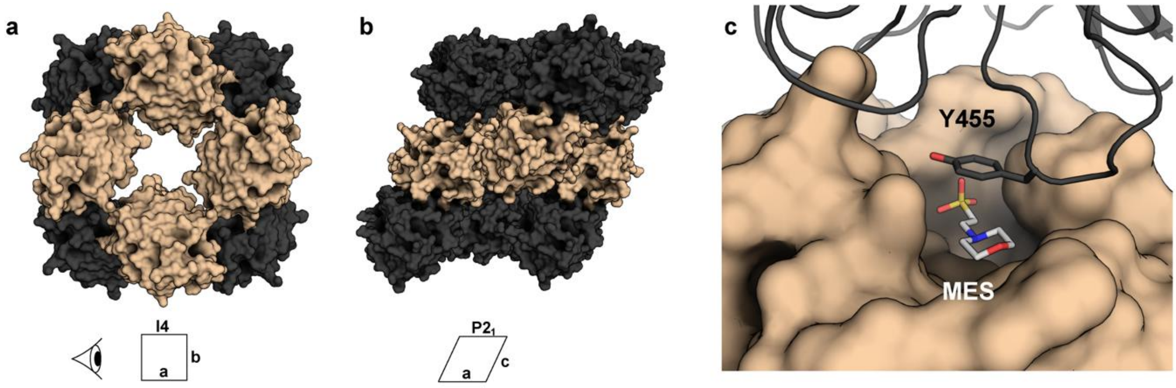

Figure S2: Comparison of different crystal packaging of murine TRIM21 **a)** Space group I4 **b)** space group P2$_1$ The colours represent different layers in each crystal form **c)** illustrates the blocking of the primary binding pocket in the space group P2$_1$ by a symmetry related molecule.

3. Comparison of antibody Fc-region binding to human and murine TRIM21

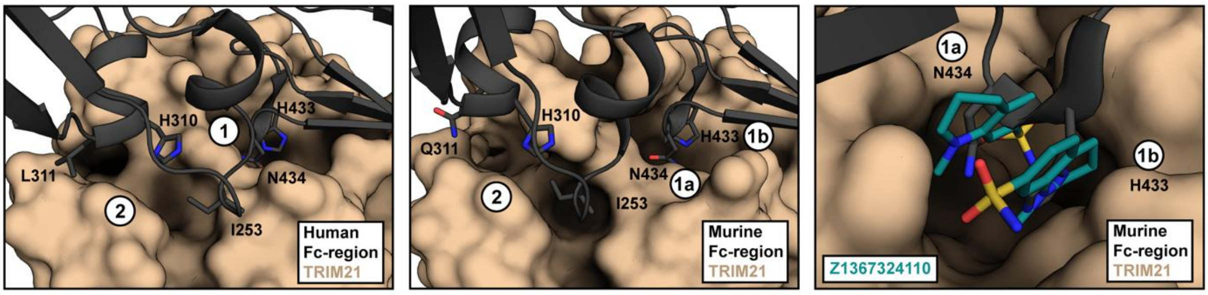

Figure S3: The left panel shows the interaction between the antibody Fc-region and TRIM21 from human, while the middle panel displays the same interaction in mouse. The right panel shows a close up of the mouse AB binding in comparison with the two sub binding sites found in the fragment screen. The primary and secondary (sub) binding sites are indicated in each panel by 1a and 1b.

4. Electrostatic surface potential of binding sites

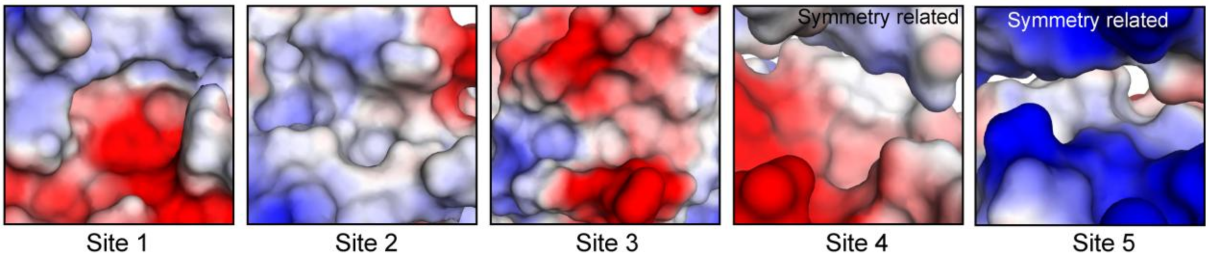

Figure S4: Electrostatic surface potential of binding sites. For site 4 & 5 the symmetry related molecules are shown.

5. Recently published human TRIM21 mutant structure (PDB: 8Y58)

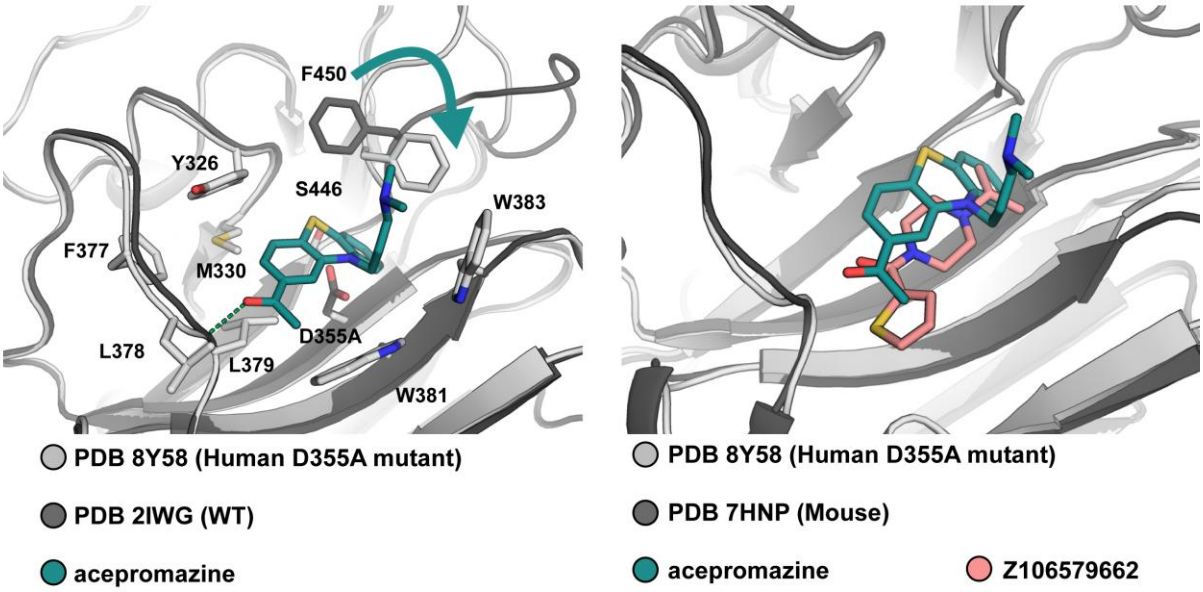

Figure S5: The left panel shows the recently published PDB 8Y58, with an acepromazine bound to human TRIM21. The compound exhibits greater activity on the D355A mutant compared to the wild type, likely due to steric hindrance from the larger side chain or less favourable charge interactions. The binding mode is notable, as it differs from what has been observed in most fragments in our screen as the aromatic rings systems do not bind in a manner that facilitates π-stacking. Additionally, the conformational change of F450 by approximately 180° is intriguing, although this may also result from the mutation. The right panel shows an overlay of PDB 8Y58 with PDB 7HNP, a fragment that binds similarly.

6. NanoBRET assay validation and results

a

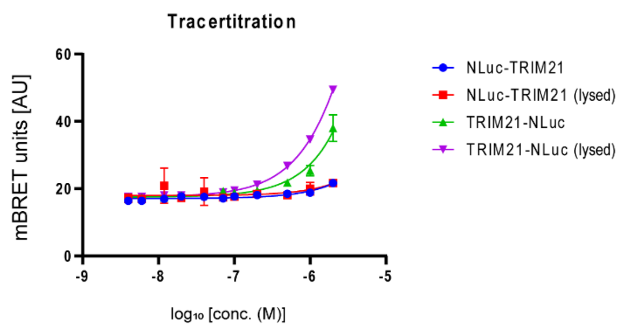

b

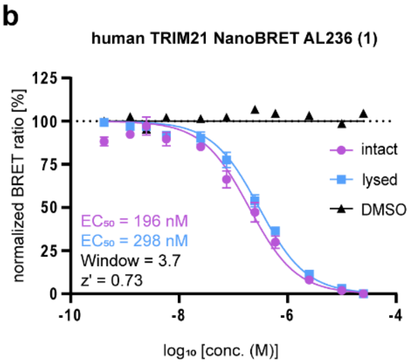

c

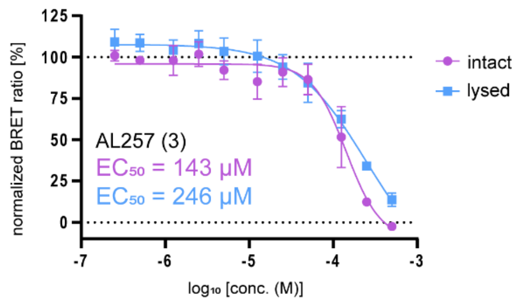

d

	Log EC50		
	value	+ error	- error
AL236 (1) lysed	-6,53	0,04	0,04
AL236 (1) intact	-6,71	0,08	0,08
AL257 (3) intact	-3,61	1,09	0,26
AL257 (3) lysed	-3,85	0,23	0,10
	EC50		
	value	+ error	- error
AL236 (1) lysed	2,98E-07	3,15E-08	2,85E-08
AL236 (1) intact	1,96E-07	4,05E-08	3,36E-08
AL257 (3) lysed	2,46E-04	2,76E-03	1,12E-04
AL257 (3) intact	1,43E-04	9,82E-05	2,90E-05

f

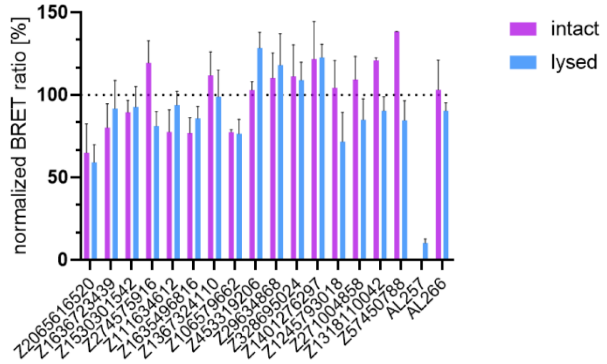

	Normalized NanoBRET response at 500 µM			
	intact		lysed	
compound	value	error	value	error
Z2065616520	65,0	12,1	59,1	8,2
Z1636723439	80,2	10,5	91,7	13,2
Z1530301542	89,4	5,0	92,7	9,4
Z274575916	119,4	10,3	81,2	6,4
Z111634612	77,6	8,9	93,8	6,5
Z1635496816	76,9	6,2	85,6	5,3
Z1367324110	111,9	10,8	98,9	11,3
Z106579662	77,5	1,1	76,5	6,2
Z453319206	102,9	3,7	128,5	6,4
Z29634868	110,3	11,6	118,1	14,5
Z328695024	111,2	14,4	109,0	7,4
Z1401276297	121,7	17,0	122,7	5,9
Z1245793018	104,2	12,7	71,7	13,5
Z271004858	109,3	10,7	84,9	9,0
Z1318110042	120,9	1,2	90,5	5,7
Z57450788	138,4	0,0	84,6	9,1
AL257 (3)	-19,6	1,1	10,1	2,0
AL266 (4)	103,0	13,8	90,5	3,4

Figure S6: (a) NanoBRET Tracer titration against cells expressing human full length TRIM21 with N-terminally or C-terminally fused Nano-Luciferase in intact and lysed cell mode. As the C-terminal NanoLuc fusion provided a better assay window in both intact and lysed mode, all competition assays were performed with the human TRIM21-Nano Luciferase construct **(b)** EC_{50} determination of AL236 **(1)** in intact/lysed cell mode **(c)** EC_{50} determination of AL257 **(3)** in intact/lysed cell mode. **(d)** The table contains the exact values of the $LogEC_{50}$/EC_{50} plus errors. **(f)** Normalized NanoBRET response of fragments targeting site #1 at a concentration of 500 µM. Data points and error bars represent the mean ± standard deviation (SEM) based on three independent measurements. The values were calculated with the program Graph Pad Prism.

7. Fragments hits overview with SMILES, DSF, SPR data and PDB codes

Table S1: Fragment information including SPR response, DSF results, binding site, compound ID, chemical structure, and PDB code. Only fragments targeting site 1, 2 and 3 were validated. Some fragments were not able to be measured in DSF because of high auto fluorescence interference, and some were not able to be measured in SPR because of possible precipitation and unspecific binding to the chip at higher concentrations. They are marked on the table as N.D..

Site	Compound SMILES	Compound ID	Chemical structure	Tm-shift mTRIM21 (°C)	Tm-Shift hTRIM21 (°C)	SPR mTRIM21 (Response)	SPR hTRIM21 (Response)	PDB CODE
Site 1	CCNC1CCN(CC1)C=2C=CN=CC2	Z2065616520	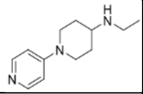	0.4	0.6	N.D.	N.D.	7HN1
Site 1	CNC(=O)C1CNCCO1	Z1636723439	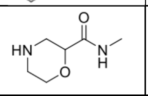	0.4	0.1	142.5	46.15	7HMH
Site 1 & 5	CCC1=CSC(=N1)C2=CNC=N2	Z1530301542	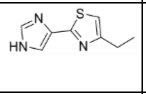	-0.1	0.1	39.06	45.06	7HMP
Site 1	CC=1C=C(N=C(C)N1)N2CCNCC2	Z274575916	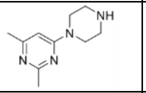	0	0.2	164.1	76.44	7HMG
Site 1	CC(C)C1=CC(=O)N2N=C(C)N=C2N1	Z111634612	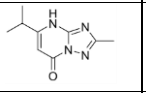	-0.4	-0.4	63.45	61.58	7HLA
Site 1	CC(=O)NC=1C=C2OCOC2=CC1C	Z1635496816	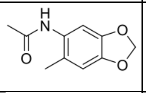	0	0.1	140.9	148.8	7HM7
Site 1	CN1CCCC=2C=CC(=CC12)S(=O)(=O)N	Z1367324110	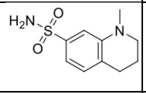	-0.6	-0.7	76.48	97.24	7HMF
Site 1	CC(C)N1CCN(CC1)C(=O)C2=CC=CS2	Z106579662	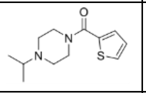	0.2	0.6	157.7	37.03	7HNP
Site 1	O=C(NC=1C=CC=2N=CC=CC2C1)C=3C=CC=CC3	Z453319206	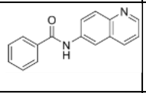	N.D.	N.D.	N.D.	N.D.	7HLH
Site 1	CCCC1=CC(=O)OC=2C=C(OC(=O)C)C=CC12	Z29634868	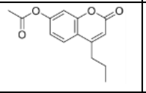	N.D.	N.D.	78.57	95.03	7HLP
Site 1	CC(C)N(C)C=1N=CN=C2N(C)N=CC12	Z328695024	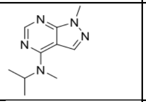	-0.6	-0.6	47.77	48.11	7HLT
Site 1	C1CC2(CCN(C2)C=3C=NC=CN3)CO1	Z1401276297	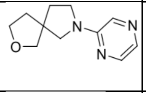	0.2	0.6	374.2	640.3	7HLD
Site 1	CN(C)C=1C=CN=CC1	Z1245793018	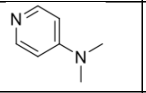	0.1	0	131.3	47.83	7HNZ
Site 1	NC=1C=CC(=CC1)S(=O)(=O)NC=2C=CC=CN2	Z271004858	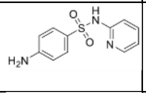	-0.1	-0.1	106.3	79.01	7HMK
Site 1	CC(=O)NC=1C=CC(CN2CCOCC2)=CC1	Z57450788	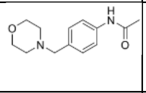	0	0.4	64.68	43.78	7HNO
Site 1 & 2	CC#CCN(C)C1CCS(=O)(=O)CC1	Z1318110042	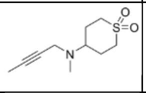	0.1	0	68.46	20.83	7HN8
Site 2	CCC1=NC=C(CNC)S1	Z1267773633	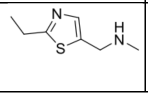	-0.4	-0.4	58.5	45.3	7HMQ

Site	Compound SMILES	Compound ID	Chemical structure	Tm-shift mTRIM21 (°C)	Tm-Shift hTRIM21 (°C)	SPR mTRIM21 (Response)	SPR hTRIM21 (Response)	PDB CODE
Site 2	CCC(NC)C=1C=CN=CC1	Z1250132788	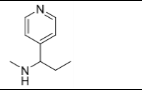	0	-0.1	53.11	50.74	7HO9
Site 2	OC(=O)COC=1C=CC(F)=CC1	Z56978034	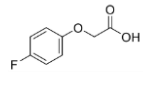	-0.2	0	107.1	99.74	7HMV
Site 2	OC(=O)COCC=1C=CC=CC1	Z993967070	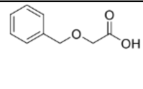	-0.3	0	63.84	56.18	7HO8
Site 2	CS(=O)(=O)NCC1CCNCC1	Z1741966151	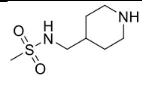	0.2	0.1	29.2	18.48	7HMU
Site 2	C(NC=1C=CN=CC1)C2CCCO2	Z1267882044	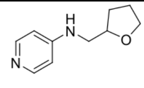	0	-0.1	85.46	39.64	7HMR
Site 2	CC(CS(=O)(=O)N)C=1C=CC=CC1	Z1407673036	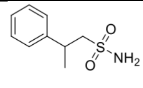	-0.3	-0.4	43.51	47.04	7HM4
Site 2	CC(O)C=1C=NN(C1)C2CCCC2	Z2004563941	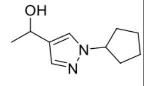	-0.2	0	41.62	39.09	7HMT
Site 2	CC(=O)N1CC=2C=CC(N)=CC2C1	Z1354416068	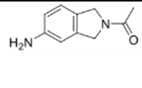	0	0.1	104.9	107.4	7HML
Site 2	CS(=O)(=O)NCCC=1C=CC=CC1	Z45617795	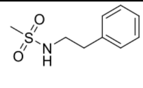	-0.3	-0.2	50.84	53.67	7HN5
Site 2	O=C(N1CCOCC1)C2=CSN=N2	Z741055844	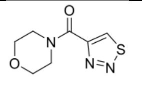	-0.2	-0.1	32.54	28.98	7HLU
Site 2	COCC(=O)NCC=1C=CC=CC1	Z31478538	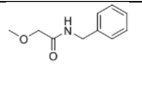	-0.3	-0.3	37.9	38.23	7HNB
Site 2	CC1=NSC(=N1)N2CCCNCC2	Z1578665941	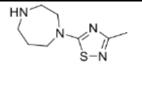	-0.1	0.1	154	59.22	7HMJ
Site 2	CC(=O)NCCC=1C=CC=CC1C	Z52314092	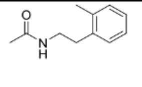	-0.3	-0.4	79.62	75.76	7HND
Site 2	CS(=O)(=O)CC1=NC=2C=CC=CC2N 1	Z126932614	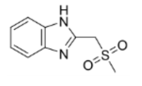	-0.1	-0.2	65.26	80.74	7HMS
Site 2	CC(=O)NC1CNC=2C=CC=CC2C1	Z1492796719	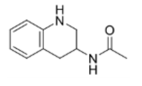	-0.1	-0.1	54.86	55.11	7HMW
Site 2	NS(=O)(=O)C=1C=CC=CC1OC(F)F	Z1003146540	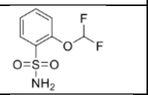	-0.1	0.5	35.49	38.84	7HO4
Site 2	CCOC(=O)CN1CCS(=O)(=O)CC1	Z793778804	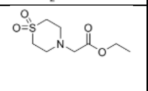	0	0.1	30.52	29.17	7HM1
Site 2	OCC1=CN(N=N1)C=2C=CC(Cl)=CC 2	Z1374778753	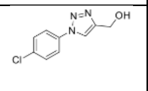	N.D.	N.D.	169.5	114.8	7HMB
Site 2	CC(NC(=O)C1CC1)C=2C=CC=NC2	Z220996120	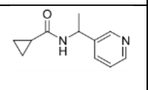	-0.1	0	171.5	149.8	7HNC
Site 2	CS(=O)(=O)NCCC=1C=CC(F)=CC1	Z45705015	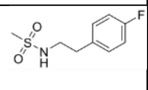	-0.5	-0.2	46.34	48.3	7HLB

Site	Compound SMILES	Compound ID	Chemical structure	Tm-shift mTRIM21 (°C)	Tm-Shift hTRIM21 (°C)	SPR mTRIM21 (Response)	SPR hTRIM21 (Response)	PDB CODE
Site 2	CC(NS(=O)(=O)C)C=1C=CC=CC1Cl	Z133729708	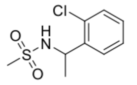	-0.8	-0.7	60.52	74.68	7HNX
Site 2	CC(NS(=O)(=O)C)C=1C=NN(C)C1C	Z369263636	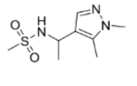	-0.1	0.1	35.15	36.54	7HO1
Site 2	CN1CCOC(CNC2=NC=CS2)C1	Z1002247062	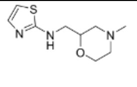	-0.1	-0.2	105	20.86	7HN2
Site 2	CC1=NC=CN1CC=2C=CC=C(C#N)C2	Z319545618	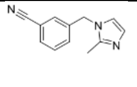	-0.3	0	30.53	11.39	7HME
Site 2	Cl.CCOC=1C=CC=CC1N2CCNCC2	Z425387594	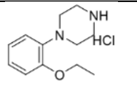	-0.1	0	98.47	61.98	7HMD
Site 2	NC(=S)NC=1C=CC=CC1OC(F)(F)F	Z291279160	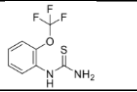	-0.4	-0.2	37.66	45.42	7HMA
Site 2	CN(C)C(=O)C=1C=CC=2NCCCC2C1	Z1222331430	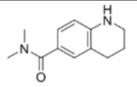	-0.2	-0.2	51.36	70.74	7HM9
Site 2	O=C1CN(CC=2C=CC=CC2)CC(=O)N1	Z3220108246	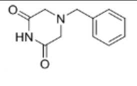	-0.5	-0.1	58.83	60.94	7HO6
Site 2	CCC(=O)N1CCCC(C1)NS(=O)(=O)C	Z405825414	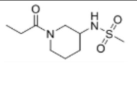	-0.2	0	27.91	27.61	7HO3
Site 2	CC(=O)N1CCCC(CNS(=O)(=O)C)C1	Z438096750	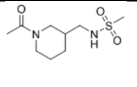	0	-0.2	42.61	40.93	7HLW
Site 2	CN1CCC(OC=2C=CC=C(F)C2)C1=O	Z1217960891	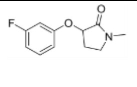	-0.4	-0.4	74.94	55.59	7HN0
Site 2	CCC1=NC(CN2C=CC=CC2=O)=NO1	Z1162778919	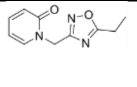	-0.2	-0.1	48.28	44.97	7HNJ
Site 2	O=C(N1CCCCCC1)C=2C=CC=3OCOC3C2	Z31432226	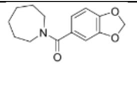	-0.6	-0.8	N.D.	N.D.	7HNN
Site 2	OC(=O)C1CCCN(C1)C(=O)CC=2C=CC(F)=CC2	Z85893853	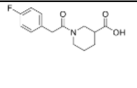	-0.4	-0.1	60.46	49.09	7HMZ
Site 2	CCCN1C(NC(=O)CC)=NC=2C=CC=CC12	Z29077827	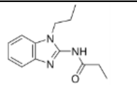	-0.6	-0.7	54.53	48.46	7HLM
Site 2	CC=1C=CC=C(C1)C(=O)N2CCC(CC2)C(=O)N	Z32400357	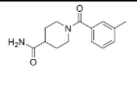	-0.4	-0.1	57.58	52.75	7HM2
Site 2	CC1CN(C(C)CO1)C(=O)C=2C=NSN2	Z768399682	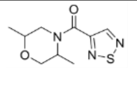	-0.3	0	24.2	20.05	7HNM
Site 2	CN(CC(=O)N1CCCCC1)S(=O)(=O)C	Z363071686	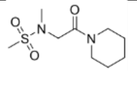	-0.1	-0.2	39.85	20.46	7HO0
Site 2	CN(C)C(=O)N1CCOCC21CCOC2	Z1998104358	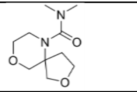	0	0.1	24.94	21.66	7HN6
Site 2	FC1=CCCN(C1)C(=O)N2CCOCC2	Z1689442171	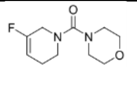	-0.2	-0.2	-16.11	-67.09	7HNF

Site	Compound SMILES	Compound ID	Chemical structure	Tm-shift mTRIM21 (°C)	Tm-Shift hTRIM21 (°C)	SPR mTRIM21 (Response)	SPR hTRIM21 (Response)	PDB CODE
Site 2	CC1=NN(C)C=2C(O)=NN=CC12	Z2301438417	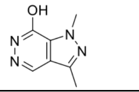	-0.3	-0.2	36.56	39.38	7HN3
Site 2	CC(=O)N1C[C@H]2CC[C@H]1C=3C=CC=CC23	Z2017861827	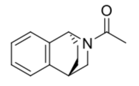	-0.7	-0.2	37.47	47.45	7HLI
Site 2	CNC(=O)C=1C=CC(=CC1)S(=O)(=O)N	Z165170770	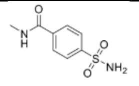	-0.4	0	49.37	49.74	7HMM
Site 2	OC1CN(C1)C(C=2C=CC=CC2)C=3C=CC=CC3	Z1696844792	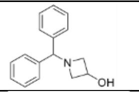	-0.5	-0.8	107.8	122.6	7HLE
Site 2	O=S1(=O)CCN(CC2=CC=CO2)CC1	Z1568344634	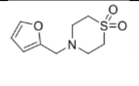	-0.2	0	60.76	40.64	7HNG
Site 2	CN(C)C1=NOC(=N1)C2CCNCC2	Z1742054999	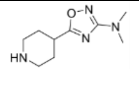	0	-0.2	84.81	41.66	7HMI
Site 2	CCC1=NN=C(NC(=O)C2CCCO2)S1	Z26823525	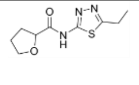	-0.2	-0.4	54.46	48.81	7HLN
Site 2	CC=1C=C(CN2N=C(C)C=CC2=O)ON1	Z1079512010	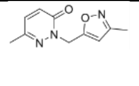	-0.3	-0.1	50.4	45.75	7HLC
Site 2	CC(=O)N1CCCC2(C1)CCCCCC2	Z1275599911	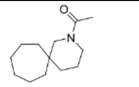	-1.3	-0.9	32.73	30.21	7HNK
Site 2	CN(C)C=1C=C(CNC(=O)C)C=CN1	Z374427992	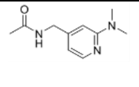	-0.1	-0.1	50.3	46.45	7HM0
Site 2	COC1CN(CC2=CN=CS2)CCC1C	Z1787761777	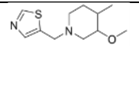	-1.6	-0.3	-42.9	-50.75	7HN9
Site 2	CS(=O)(=O)NC1CCOC2(CCCC2)C1	Z1446981563	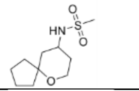	-0.4	-0.1	27.71	28.35	7HNW
Site 2	CC=1C(Cl)=CC=CC1NC(=O)[C@@H]2CCCO2	Z1545312521	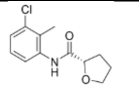	-0.9	-0.7	31.58	24.5	7HNR
Site 2	COC1CN(C1)S(=O)(=O)N2CCCC2	Z1343518214	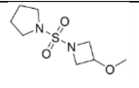	-0.1	0.2	38.32	34.32	7HNE
Site 3	CCC(=O)NC1=NC(C)=CS1	Z30820160	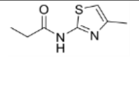	-0.5	-0.2	15.6	46.05	7HO7
Site 3	CNS(=O)(=O)CC=1C=C(C)ON1	Z763030030	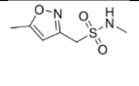	-0.2	-0.1	42.23	44.17	7HMX
Site 3	Cl.O=C(N1CCCC1)C=2C=NNC2	Z1266933824	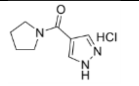	-0.3	-0.1	127.7	193.6	7HO5
Site 3	COC=1C=CC=CC1C2=NN=CO2	Z1359419878	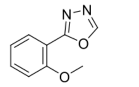	-0.6	-0.4	53.75	57.54	7HM3
Site 3	O=C(NC1CC1)C=2C=CC=3OCOC3C2	Z32367954	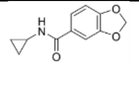	-0.3	-0.6	59.37	68.73	7HLX
Site 3	O=C(C1CC1)N2CCN(CC2)C(=O)C3=CC=CO3	Z32327641	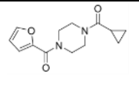	-0.2	0	37.82	35.48	7HLQ

Site	Compound SMILES	Compound ID	Chemical structure	Tm-shift mTRIM21 (°C)	Tm-Shift hTRIM21 (°C)	SPR mTRIM21 (Response)	SPR hTRIM21 (Response)	PDB CODE
Site 3	NC(=O)N1CCN(CC1)C(=O)C2=CC=CO2	Z198194396	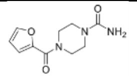	-0.2	-0.2	38.54	34.19	7HN7
Site 3	O=C(NC1CC1)C2=C2C=NN3C=CC=NC23	Z285642082	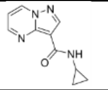	-0.3	-0.2	44.74	43.71	7HLK
Site 3	CCN1C=C(NC(=O)C2CCC2)C=N1	Z373768900	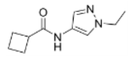	-0.4	-0.3	20.02	21.88	7HNI
Site 3	O=C(CN1CCCC1=O)NC2=NC=CS2	Z79432418	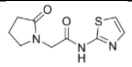	-0.4	-0.3	63.39	61.55	7HLV
Site 3	FC(F)OCC(=O)N1CC2CCCC2C1	Z2072621991	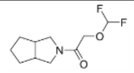	0	-0.2	46.61	28.49	7HNS
Site 3	CC=1C=C(N=C(CO)N1)N2CCCCC2	Z1980894300	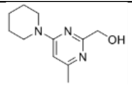	-0.2	0	77.01	66.59	7HLJ
Site 4	CS(=O)(=O)C1=NC=CN1CC2=CSC=N2	Z1328968520	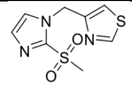					7HLL
Site 4	COC(=O)[C@@H]1C[C@@H](O)CN1C(=O)C2=CC=CO2	Z1614545742	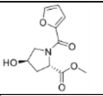					7HNU
Site 5	CCC1(O)CCN(C1)C=2C=CC(F)=CN2	Z2092555279	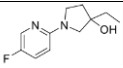					7HLF
Site 5	C[C@]1(O)C[C@H](C1)NC=2N=CC=CC2F	Z2365130785	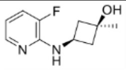					7HLG
Site 5	CC(=O)N1CCN(CC1)C(=O)N	Z90122368	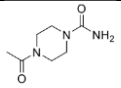					7HLO
Site 5	CC=1C=CC(NC(=O)C=2C=CC=C(F)C2)=CC1O	Z1430613393	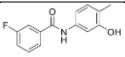					7HLR
Site 5	CC(C)C(=O)NC=1C=CC=C(C1)C(=O)N	Z29177127	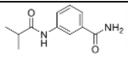					7HLS
Site 5	OCC=1C=CC(=CC1)N2C=NC=3C=CC=CC23	Z235449082	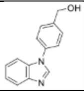					7HLY
Site 5	OC(=O)CC=1C=CC(=CC1)C=2C=CC=CC2	Z256709358	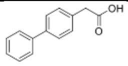					7HLZ
Site 5	COC(=O)N1CCN(CC1)C=2C=CC(F)=CC2	Z192955056	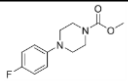					7HM5
Site 5	FC=1C=CC(NC2CCOCC2)=NC1	Z1172243962	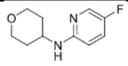					7HM6
Site 5	CNC=1C=CC=CC1S(=O)(=O)C	Z285782452	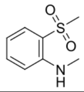					7HM8
Site 5	COC=1C=CC=2SC(N)=NC2C1	Z1954800564	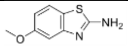					7HMC
Site 5	COCC1=NN=C(N)S1	Z57478994	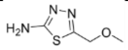					7HMN

Site	Compound SMILES	Compound ID	Chemical structure	Tm-shift mTRIM21 (°C)	Tm-Shift hTRIM21 (°C)	SPR mTRIM21 (Response)	SPR hTRIM21 (Response)	PDB CODE
Site 5	NC(=O)N1CCN(CC1)C=2C=CC(F)=CC2	Z198194394	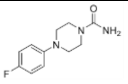					7HMO
Site 5	CC(OC=1C=CC(=CC1)C=2C=CC=CC2)C(=O)O	Z111782404	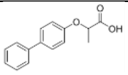					7HMY
Site 5	CC=1C=CC=C(C1)C(=O)NCC(=O)O	Z56827661	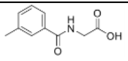					7HN4
Site 5	CC1(C)CN(CC1O)C=2N=CC=CC2F	Z1929757385	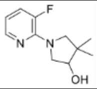					7HNA
Site 5	CN1C=CC(=N1)C(=O)NC[C@@H]2CCCO2	Z2643472210	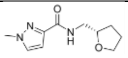					7HNH
Site 5	CC1=NN=C(S1)N2CCCCC2	Z1251207602	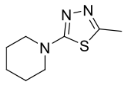					7HNL
Site 5	FC=1C=CC(=CC1)N2CCN(CC2)C(=O)C3CC3	Z30620520	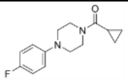					7HNQ
Site 5	CC(=O)NC=1C=CC2=CNN=C2C1	Z383202616	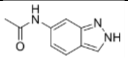					7HNT
Site 5	CC1=CC(=NO1)C(=O)NC=2C=NN(C)C2	Z404993336	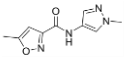					7HNV
Site 5	O=C(NC=1C=CC=CC1)NC=2C=CC=NC2	Z44592329	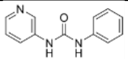					7HNY
Site 5	OC=1C=CC(CCNC(=O)C=2C=CC=CN2)=CC1	Z730649594	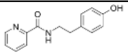					7HO2
Site 5	CC(=O)N1CCN(CC1)C=2C=CC(Cl)=CC2	Z275165822	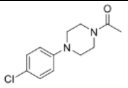					7HOA
Ground state								7HOB

8. Chemical Synthesis & Analytical data

Unless otherwise stated, all commercial reagents were purchased from BLD Pharm and Enamine with a purity ≥ 95% and were used without further purification. All solvents were analytical grade and purchased from Thermo Scientific and experimental procedures were carried out according to common techniques in an inert gas atmosphere (argon 5.0) under magnetic stirring. Silica gel-coated plates with fluorescent indicator (DC silica gel 60 F_{254}, neutral, from the company *Merck*) were used for reaction control. The visualization of the substances to be investigated were visualized with the aid of UV light. Flash column chromatography was performed with a puriFlash® XS 420 system from *Interchim* using puriFlash® prepacked C18-HP or SI-HP cartridges (30 µm spherical silica) with technical and HPLC grade solvents. UV absorption was detected between 200 and 400 nm. Preparative purification by HPLC was carried out on an Agilent 1260 Infinity II device using an Eclipse XDB-C18 (Agilent, 21.2 x 250mm, 7µm) reversed phase column. A suitable gradient (flow rate 21 mL/min) was used, with 0.1% (v/v) TFA in water (A) and 0.1% (v/v) TFA in acetonitrile (B), as a mobile phase. Determination of the compound purity by HPLC was carried out on an Agilent 1260 Infinity II device with a 1260 DAD HS detector (G7117C; 254 nm, 280 nm, 310 nm) and a LC/MSD device (G6125B, ESI pos. 100-1000). Compounds **2** - **7** and **9** were analyzed on a Poroshell 120 EC-C18 (Agilent, 3 x 150 mm, 2.7 µm) reversed phase column using 0.1% formic acid in water (A) and 0.1% (v/v) formic acid in acetonitrile (B) as a mobile phase. The following gradient was used: 0 min. 5% B - 2 min. 5% B - 8 min. 98% B (flow rate of0.5 mL/min.). UV-detection was performed at 254, 280 and 310 nm and all compounds used for further biological characterizations showed > 95% purity if not stated otherwise. For compounds **3**, **4**, **14** and **16** an analytical HPLC from Shimadzu Prominence with an SPD20A UV/Vis detector was used. Stationary phases were Luna 10 µm 100 Å, C18(2) (250×4.6 mm), and Luna 10 µm 100 Å, C18(2) (250×21.20 mm), from Phenomenex. The eluent was a mixture of ACN (A) and a 0.1% (v/v) aqueous formic acid solution (B). The following gradient was used: 0 min. 10% A - 8 min. 95% A (flow rate of 1 mL/min). UV-detection was performed at 254 and 280 nm and all compounds used for further biological characterizations showed > 95% purity if not stated otherwise. NMR spectroscopic measurements were performed at ambient temperatures using DMSO-d_6 as solvent. The chemical shifts δ are given in parts per million (ppm), the scalar coupling constants J in Hz. The residual signal of the incompletely deuterated solvent served as a reference for the ^{1}H- and ^{13}C-NMR-spectra (δ (^{1}H) = 2.50 ppm, δ (^{13}C {^{1}H}) = 39.52 ppm). The following abbreviations are used to characterize the observed signal multiplicities: s (singlet), d (doublet), dd (doublet of doublet), ddd (doublet of doublet of doublet), t (triplet), td (triplet of doublet), q (quartet), qd (quartet of dublet), p (pentet) and m (multiplet).

8.1 Chemistry

8.1.1 *General procedure I for amide coupling reaction*

The corresponding acid (1.0 eq), O-(7-Azabenzotriazol-1-yl)-*N,N,N',N'*-tetramethyl uronium hexafluoro-phosphate (HATU) (1.2 eq) and *N,N′* -diisopropylethylamine (DIPEA) (3 eq) were dissolved in dry DMF (3-5 mL). The solution was stirred at ambient temperatures for 30 min. Then, the amine (1.1 eq) was added and the reaction solution was stirred for 16 h at 40 °C. Subsequently, the solvent was removed under reduced pressure and the crude product was purified *via* flash chromatography on silica using acetonitrile/water or dichloromethane/methanol as eluent.

8.1.2 *General procedure II for deprotection of Boc-protected amines*

The corresponding Boc-protected amine was dissolved in 4 mL dry DCM, and TFA (20% (v/v)) was added. The reaction solution was stirred for 1 h at ambient temperature. Subsequently, the solvent was removed under reduced pressure. If necessary, the crude product was purified *via* flash chromatography on silica using acetonitrile/water as eluent.

8.1.3 *Synthesis of positive control **1** (**AL236**)*

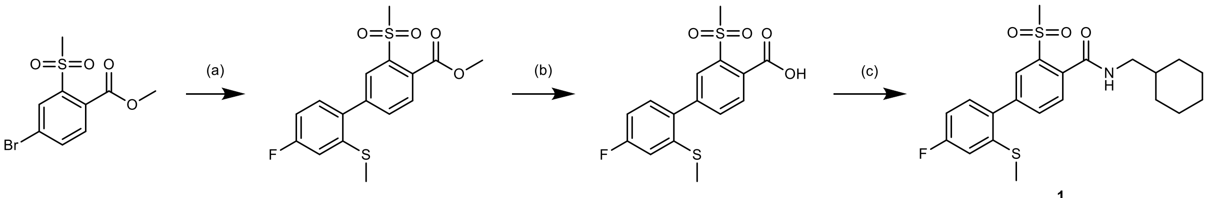

Reagents and conditions: (a) (4-fluoro-2-(methylthio)phenyl)boronic acid, [1,1′-Bis(diphenylphosphino)ferrocen]dichlorpalladium(II) (Pd(dppf)Cl_2), K_2CO_3, and 1,4-dioxane/water (4:1), 90 min, 110 °C; (b) lithium hydroxide monohydrate, and methanol/water (4:1), 16 h, 75 °C; (c) cyclohexylmethanamine, HATU, DIPEA, and dry DMF, 16 h, 40 °C.

Methyl 4'-fluoro-3-(methylsulfonyl)-2'-(methylthio)-[1,1'-biphenyl]-4-carboxylate (**6**)

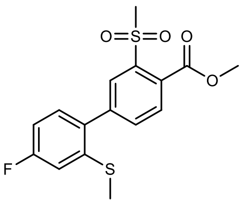

Methyl 4-bromo-2-(methylsulfonyl)benzoate (**5**) (500 mg, 1.71 mmol) was dissolved in 1,4-dioxane/water (12.5 mL, 4:1). The solution was degassed. Then, (4-fluoro-2-(methylthio)phenyl)boronic acid (349 mg, 1.88 mmol), K_2CO_3 (472 mg, 3.41 mmol) and Pd(dppf)Cl_2 (62.4 mg, 85.3 µmol) were added in succession. The reaction mixture was stirred for 90 min at 110 °C. Afterwards, the solvent was removed under reduced pressure. The crude product was dissolved in ethyl acetate and filtered over Celite®. The filtrate was washed with brine and dried over $MgSO_4$. Subsequently, the solvent was removed under reduced pressure and the crude product was purified

via flash chromatography on silica using dichloromethane/methanol as eluent. The product was isolated as an off-white solid in a yield of 83%. **^{1}H NMR** (400 MHz, DMSO-*d$_6$*): *δ* = 7.98 (t, *J* = 1.1 Hz, 1H), 7.85 – 7.83 (m, 2H), 7.36 (dd, *J* = 8.5, 6.0 Hz, 1H), 7.25 (dd, *J* = 10.2, 2.5 Hz, 1H), 7.12 (td, *J* = 8.5, 2.6 Hz, 1H), 3.90 (s, 3H), 3.40 (s, 3H), 2.46 (s, 3H) ppm. **MS (ESI+)**: *m/z* = 377.00 $[M+Na]^+$; calc.: 377.03.

4'-fluoro-3-(methylsulfonyl)-2'-(methylthio)-[1,1'-biphenyl]-4-carboxylic acid (**7**)

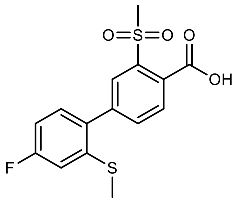

Methyl 4'-fluoro-3-(methylsulfonyl)-2'-(methylthio)-[1,1'-biphenyl]-4-carboxylate (**6**) (499 mg, 1.41 mmol) and lithium hydroxide monohydrate (237 mg, 5.63 mmol) were dissolved methanol/water (10 mL , 4:1). The reaction mixture was stirred for 16 h at 75 °C. Subsequently, the solvent was removed under reduced pressure and the crude product was purified *via* flash chromatography on silica using acetonitrile/water as eluent. The product was isolated as a colorless solid in a yield of 90%. **^{1}H NMR** (400 MHz, DMSO-*d$_6$*): *δ* = 7.74 (d, *J* = 1.4 Hz, 1H), 7.55 – 7.44 (m, 2H), 7.25 (dd, *J* = 8.4, 6.1 Hz, 1H), 7.17 (dd, *J* = 10.3, 2.6 Hz, 1H), 7.06 (td, *J* = 8.5, 2.6 Hz, 1H), 3.49 (s, 3H), 2.43 (s, 3H) ppm. **MS (ESI+)** *m/z* = 323.05 $[C_{15}H_{12}FO_3S_2\cdot]^+$; calc.: 323.03.

N-(cyclohexylmethyl)-4'-fluoro-3-(methylsulfonyl)-2'-(methylthio)-[1,1'-biphenyl]-4-carboxamide (**1**) (**AL236**)

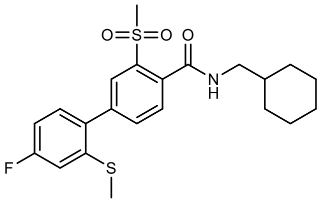

Compound **1** was synthesized according to general procedure I, using **7** (50.0 mg, 147 µmol) as starting material. The product was isolated as a colorless solid in a yield of 83%. **^{1}H NMR** (500 MHz, DMSO-d$_6$): *δ* = 8.69 (t, *J* = 5.8 Hz, 1H), 7.91 (d, *J* = 1.8 Hz, 1H), 7.76 (dd, *J* = 7.8, 1.8 Hz, 1H), 7.58 (d, *J* = 7.7 Hz, 1H), 7.31 (dd, *J* = 8.4, 6.0 Hz, 1H), 7.23 (dd, *J* = 10.3, 2.6 Hz, 1H), 7.11 (td, *J* = 8.4, 2.6 Hz, 1H), 3.40 (s, 3H), 3.13 – 3.05 (m, 2H), 2.46 (s, 3H), 1.82 – 1.74 (m, 2H), 1.73 – 1.66 (m, 2H), 1.65 – 1.50 (m, 2H), 1.27 – 1.10 (m, 3H), 0.99 – 0.88 (m, 2H) ppm.**^{19}F NMR** (471 MHz, DMSO-*d$_6$*): *δ* = -112.49 – -112.55 (m) ppm. **^{13}C{^{1}H} NMR** (126 MHz, DMSO-*d$_6$*): *δ* = 167.3, 162.6 (d, *J* = 246.6 Hz), 140.3, 139.9 (d, *J* = 8.2 Hz), 138.0, 137.0, 134.2, 133.6 (d, *J* = 3.1 Hz), 131.7 (d, *J* = 9.0 Hz), 129.8, 129.2, 112.1, 111.9, 111.7, 111.5, 45.5, 45.0, 37.2, 30.5, 26.1, 25.5, 15.0 ppm. **MS (ESI+)**: *m/z* = 436.05 $[M+H]^+$; calc.: 436.15.

*8.1.4 Synthesis of tracer **2** (**AL244**)*

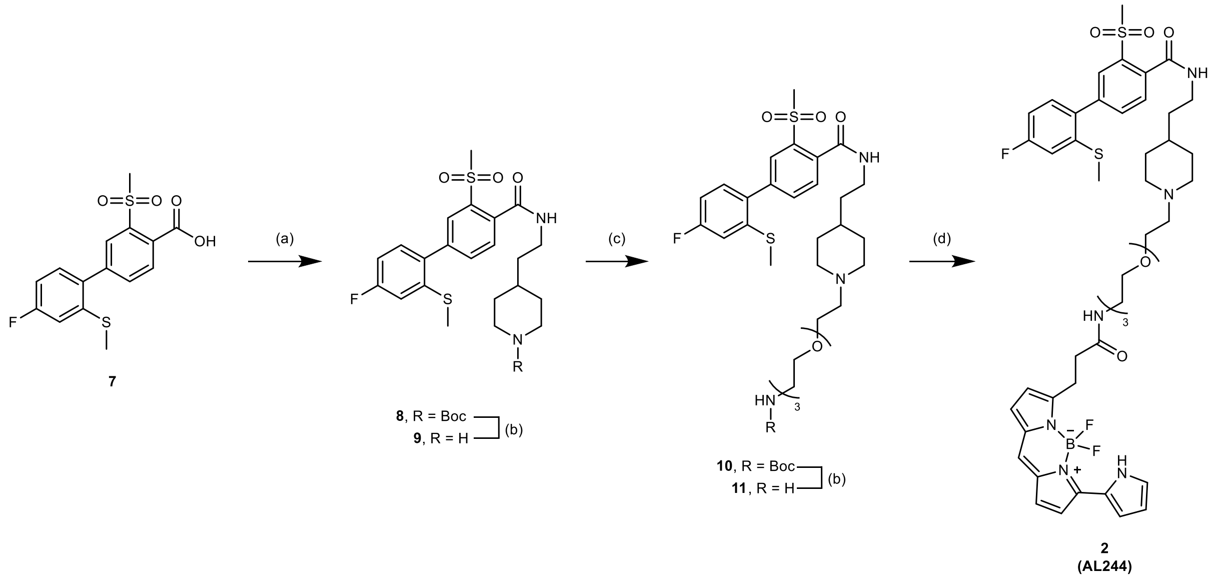

Reagents and conditions: (a) *tert*-butyl 4-(2-aminoethyl)piperidine-1-carboxylate, HATU, DIPEA, and dry DMF, 16 h, 40 °C; (b) TFA (20 vol%), and dry DCM, 1 h, rt; (c) *tert*-butyl (2-(2-(2-(2-bromoethoxy)ethoxy)ethoxy)ethyl)carbamate, DIPEA, and dry DMF, 16 h, 90 °C; (d) 2,5-dioxopyrrolidin-1-yl 3-(5,5-difluoro-7-(1*H*-pyrrol-2-yl)-5*H*-5λ^4,6λ^4-dipyrrolo[1,2-*c*:2',1'-*f*][1,3,2]diazaborinin-3-yl)propanoate, DIPEA, and dry DMF, 16 h, rt.

Tert-butyl 4-(2-(4'-fluoro-3-(methylsulfonyl)-2'-(methylthio)-[1,1'-biphenyl]-4-carboxamido)ethyl)piperidine-1-carboxylate (**8**)

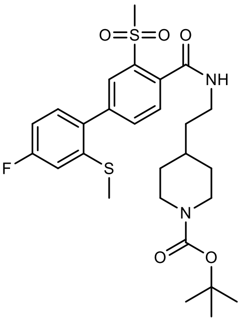

Compound **8** was synthesized according to general procedure I, using **7** (100 mg, 294 µmol) as starting material. The product was isolated as a off-white solid in a yield of 90%. **^{1}H NMR** (500 MHz, DMSO-*d$_6$*): *δ* = 8.67 (t, *J* = 5.6 Hz, 1H), 7.91 (d, *J* = 1.8 Hz, 1H), 7.76 (dd, *J* = 7.8, 1.8 Hz, 1H), 7.58 (d, *J* = 7.7 Hz, 1H), 7.31 (dd, *J* = 8.4, 6.0 Hz, 1H), 7.23 (dd, *J* = 10.2, 2.5 Hz, 1H), 7.11 (td, *J* = 8.4, 2.6 Hz, 1H), 3.93 (d, *J* = 12.9 Hz, 2H), 3.41 (s, 3H), 3.31 – 3.26 (m, 2H), 2.76 – 2.62 (m, 2H), 2.46 (s, 3H), 1.72 – 1.66 (m, 2H), 1.64 – 1.54 (m, 1H), 1.46 (q, *J* = 6.9 Hz, 2H), 1.39 (s, 9H), 0.98 (qd, *J* = 12.5, 4.3 Hz, 2H) ppm. **^{19}F NMR** (471 MHz, DMSO-*d$_6$*): *δ* = -112.48 – -112.55 (m) ppm. **MS (ESI+)**: *m/z* = 451.10 [M-Boc+H]$^+$; calc.: 451.16.

4'-fluoro-3-(methylsulfonyl)-2'-(methylthio)-N-(2-(piperidin-4-yl)ethyl)-[1,1'-biphenyl]-4-carboxamide (**9**)

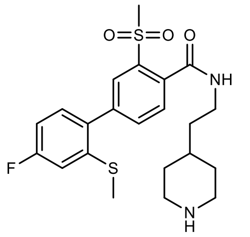

Compound **9** was synthesized according to general procedure II. The product was isolated as a colorless solid (TFA-salt) in a yield of 85%. **^{1}H NMR** (500 MHz, DMSO-d_6): δ = 8.69 (t, J = 5.7 Hz, 1H), 8.46 (s, 2H), 7.92 (d, J = 1.8 Hz, 1H), 7.77 (dd, J = 7.8, 1.8 Hz, 1H), 7.61 (d, J = 7.7 Hz, 1H), 7.31 (dd, J = 8.4, 6.0 Hz, 1H), 7.24 (dd, J = 10.2, 2.6 Hz, 1H), 7.11 (td, J = 8.4, 2.5 Hz, 1H), 3.41 (s, 3H), 3.33 – 3.25 (m, 3H), 2.82 (td, J = 12.8, 2.9 Hz, 2H), 2.46 (s, 3H), 1.90 – 1.83 (m, 2H), 1.77 – 1.65 (m, 1H), 1.48 (q, J = 6.9 Hz, 2H), 1.36 – 1.22 (m, 2H) ppm. **^{19}F NMR** (471 MHz, DMSO-d_6): δ = -73.53, -112.43 – -112.50 (m) ppm. **^{13}C{^{1}H} NMR** (126 MHz, DMSO-d_6): δ = 167.3, 162.6 (d, J = 246.8 Hz), 158.1 (q, J = 30.8 Hz), 140.4, 139.9 (d, J = 8.2 Hz), 138.0, 136.8, 134.3, 133.6 (d, J = 3.1 Hz), 131.7 (d, J = 8.9 Hz), 129.9, 129.1, 117.3 (q, J = 300.3 Hz), 112.2, 112.0, 111.7, 111.6, 45.1, 43.4, 36.2, 34.8, 30.3, 28.3, 15.0 ppm. **MS (ESI+)**: m/z = 451.15 $[M+H]^+$; calc.: 451.16.

Tert-butyl (2-(2-(2-(2-(4-(2-(4'-fluoro-3-(methylsulfonyl)-2'-(methylthio)-[1,1'-biphenyl]-4-carboxami-do)ethyl)piperidin-1-yl)ethoxy)ethoxy)ethoxy)ethyl)carbamate (**10**)

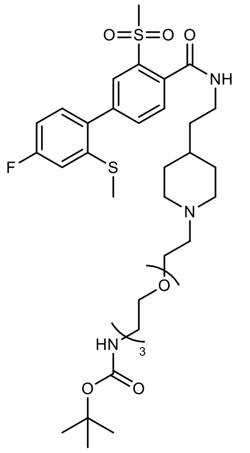

4'-fluoro-3-(methylsulfonyl)-2'-(methylthio)-N-(2-(piperidin-4-yl)ethyl)-[1,1'-biphenyl]-4-carboxamide (**9**) (60 mg, 134 µmol) and *tert*-butyl (2-(2-(2-(2-bromoethoxy)ethoxy)ethoxy)ethyl)carbamate (72 mg, 200 µmol) were dissolved in dry DMF (4 mL). Then, DIPEA (93 µL, 533 µmol) was added and the reaction solution was stirred for 16 h at 90 °C. The solvent was removed under reduced pressure and the crude product was purified *via* flash chromatography on silica using acetonitrile/water as eluent. The product was isolated as a yellow solid (TFA-salt) in a yield of 45%. **^{1}H NMR** (400 MHz, DMSO-d_6): δ = 9.35 (s, 1H), 8.71 (t, J = 5.6 Hz, 1H), 7.92 (d, J = 1.8 Hz, 1H), 7.77 (dd, J = 7.8, 1.8 Hz, 1H), 7.60 (d, J = 7.7 Hz, 1H), 7.31 (dd, J = 8.4, 6.0 Hz, 1H), 7.24 (dd, J = 10.2, 2.5 Hz, 1H), 7.12 (td, J = 8.4, 2.5 Hz, 1H), 6.76 (t, J = 5.8 Hz, 1H), 3.74 (s, 2H), 3.63 – 3.46 (m, 10H), 3.42 (s, 3H), 3.37 (t, J = 6.2 Hz, 2H), 3.30 (d, J = 6.2 Hz, 3H), 3.06 (q, J = 6.1 Hz, 2H), 2.88 (s, 1H), 2.46 (s, 3H), 1.90 (d, J = 13.4 Hz, 2H), 1.67 (s, 1H), 1.55 – 1.44 (m,

2H), 1.37 (s, 12H) ppm. ^{19}**F NMR** (377 MHz, DMSO-d_6): δ = -73.47, -112.42 – -112.51 (m) ppm. **MS (ESI+)**: *m/z* = 726.25 [M+H]$^+$; calc.: 726.33.

N-(2-(1-(2-(2-(2-(2-aminoethoxy)ethoxy)ethoxy)ethyl)piperidin-4-yl)ethyl)-4'-fluoro-3-(methylsulfonyl)-2'-(methylthio)-[1,1'-biphenyl]-4-carboxamide (**11**)

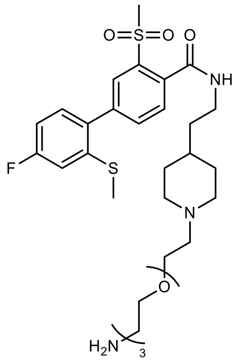

Compound **11** was synthesized according to general procedure II. The reaction was monitored *via* TLC and the crude product was used *in situ* without further purification.

N-(2-(1-(15-(5,5-difluoro-7-(1H-pyrrol-2-yl)-5H-5λ^4,6λ^4-dipyrrolo[1,2-c:2',1'-f][1,3,2]diazaborinin-3-yl)-13-oxo-3,6,9-trioxa-12-azapentadecyl)piperidin-4-yl)ethyl)-4'-fluoro-3-(methylsulfonyl)-2'-(methylthio)-[1,1'-biphenyl]-4-carboxamide (**2**) (**AL244**)

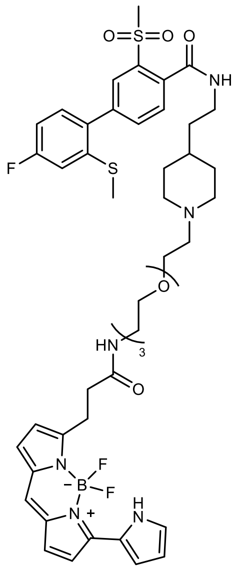

N-(2-(1-(2-(2-(2-(2-aminoethoxy)ethoxy)ethoxy)ethyl)piperidin-4-yl)ethyl)-4'-fluoro-3-(methylsulfonyl)-2'-(methylthio)-[1,1'-biphenyl]-4-carboxamide (**11**) (15.5 mg, 24.7 µmol) was dissolved in dry DMF (300 µL), then DIPEA (32.7 µL, 188 µmol) and 2,5-dioxopyrrolidin-1-yl 3-(5,5-difluoro-7-(1*H*-pyrrol-2-yl)-5*H*-5λ^4,6λ^4-dipyrrolo[1,2-*c*:2',1'-*f*][1,3,2]diaza-borinin-3-yl)propanoate (10.0 mg, 23.5 µmol) were added in succession. The reaction solution was stirred for 16 h at ambient temperature. Subsequently, the crude product was purified *via* preparative HPLC chromatography on silica gel using acetonitrile/water with added 0.1% (v/v) TFA as eluent. The product was isolated as a purple solid (TFA-salt) in a yield of 44%. ^{1}**H NMR** (500 MHz, DMSO-d_6): δ = 11.76 – 11.40 (m, 1H), 9.35 – 9.10 (m,

1H), 8.69 (t, J = 6.1 Hz, 1H), 8.01 (t, J = 6.0 Hz, 1H), 7.92 (s, 1H), 7.78 – 7.72 (m, 1H), 7.63 – 7.52 (m, 1H), 7.49 – 7.40 (m, 1H), 7.38 – 7.32 (m, 2H), 7.32 – 7.26 (m, 2H), 7.24 (dd, J = 10.2, 2.6 Hz, 1H), 7.17 (d, J = 4.7 Hz, 1H), 7.11 (td, J = 8.4, 2.6 Hz, 1H), 7.05 – 7.00 (m, 1H), 6.36 – 6.31 (m, 2H), 3.74 (t, J = 5.3 Hz, 2H), 3.57 – 3.43 (m, 10H), 3.41 (s, 3H), 3.33 – 3.21 (m, 6H), 3.14 (t, J = 8.3 Hz, 2H), 2.89 (q, J = 13.0 Hz, 1H), 2.46 (s, 3H), 1.91 (d, J = 16.4 Hz, 2H), 1.70 – 1.55 (m, 1H), 1.48 (q, J = 7.3 Hz, 2H), 1.44 – 1.32 (m, 2H), 1.29 – 1.16 (m, 2H) ppm. **^{19}F NMR** (471 MHz, DMSO-d_6): δ = -74.15,-109.90 – -114.77 (m), -138.43 – -143.60 (m) ppm. **^{13}C{^{1}H} NMR** (126 MHz, DMSO-d_6): δ = 171.2, 167.3, 162.6 (d, J = 247.2 Hz), 155.9, 150.3, 140.4, 139.9 (d, J = 8.3 Hz), 138.0, 137.0, 136.8, 134.3, 133.5 (d, J = 2.9 Hz), 133.1, 132.5, 131.7 (d, J = 8.9 Hz), 129.9, 129.0, 126.8, 126.2, 124.5, 122.9, 122.8, 119.4, 117.4, 116.2, 112.2, 112.0, 111.7, 111.6, 111.5, 69.8, 69.59, 69.51, 69.1, 64.3, 55.4, 52.5, 45.0, 38.6, 36.3, 34.7, 33.9, 30.2, 28.8, 24.1, 15.0 ppm. **HRMS (QTOF)**: m/z = 937.3803 $[M+H]^+$; calc.: 937.3770.

*8.2.5 Synthesis of merged compounds **3** (**AL257**) and **4** (**AL266**)*

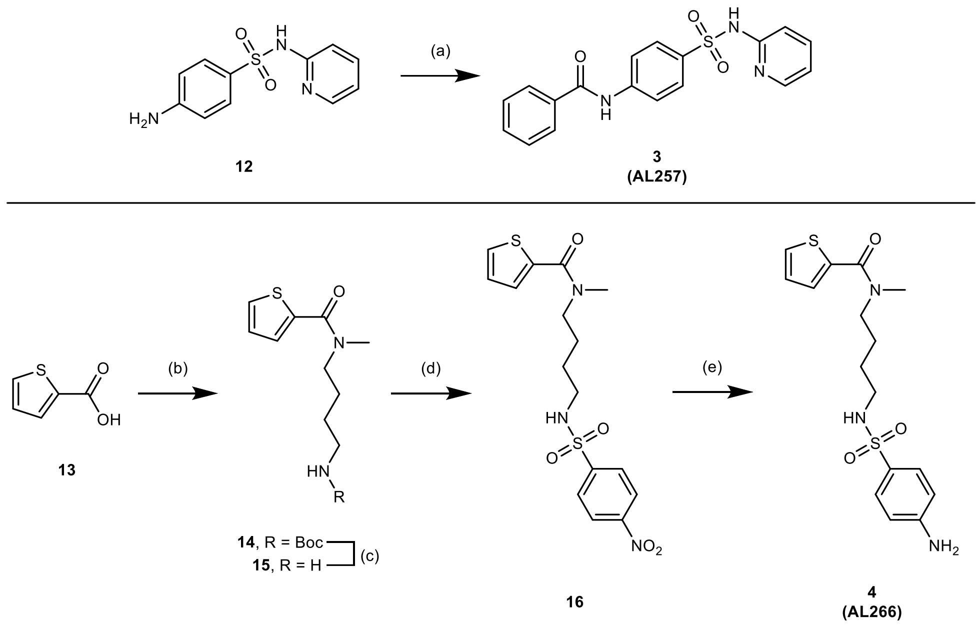

Reagents and conditions: (a benzoic acid, HATU, DIPEA, and dry DMF, 16 h, 40 °C; (b) tert-butyl (4-(methylamino)butyl)carbamate, HATU, DIPEA, and dry DMF, 16 h, 40 °C; (c) TFA (20 vol%), and dry DCM, 1 h, rt; (d) 4-nitrobenzenesulfonyl chloride, DIPEA, and dry DMF, 16 h, rt; (e) NH_4Cl, Fe powder, and methanol/water (9:1), 3 h, 75 °C.

N-(4-(N-(pyridin-2-yl)sulfamoyl)phenyl)benzamide (**3**) (**AL257**)

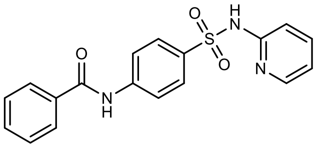

Compound **3** was synthesized according to general procedure I, using benzoic acid (50.0 mg, 410 µmol) as starting material. The product was isolated as a colorless solid in a yield of 25%. **^{1}H NMR** (500 MHz, DMSO-d_6): δ = 11.74 (s, 1H), 10.54 (s, 1H), 8.05 – 8.00 (m, 1H), 7.97 – 7.92 (m, 4H), 7.89 – 7.85 (m, 2H), 7.74 – 7.68 (m, 1H), 7.63 – 7.59 (m, 1H), 7.56 – 7.51 (m, 2H), 7.17 – 7.14 (m, 1H), 6.90 – 6.82 (m, 1H) ppm. **^{13}C{^{1}H} NMR** (126 MHz, DMSO): δ = 166.1, 153.0, 142.6, 140.2, 136.1, 134.6, 132.0, 128.9, 128.5, 127.8, 127.7, 119.8, 113.6, 112.5 ppm. MS (ESI+) *m/z* = 354.05 [M+H]$^+$; calc.: 354.10.

tert-butyl (4-(N-methylthiophene-2-carboxamido)butyl)carbamate (**14**)

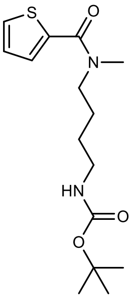

Compound **14** was synthesized according to general procedure I, using **13** (100 mg, 781 µmol) as starting material. The product was isolated as an orange solid in a yield of 90%. **^{1}H NMR** (600 MHz, DMSO-d_6): δ = 7.73 (dd, *J* = 5.0, 1.1 Hz, 1H), 7.44 (s, 1H), 7.11 (dd, *J* = 5.0, 3.7 Hz, 1H), 6.81 (t, *J* = 5.8 Hz, 1H), 3.44 (t, *J* = 7.4 Hz, 2H), 3.17 (s, 3H), 2.92 (q, *J* = 6.6 Hz, 2H), 1.55 (p, *J* = 8.0, 7.5 Hz, 2H), 1.37 (s, 9H), 1.35 – 1.32 (m, 2H) ppm. **MS (ESI+)** *m/z* = 313.10 [M+H]$^+$; calc.: 313.16.

N-(4-aminobutyl)-N-methylthiophene-2-carboxamide (**15**)

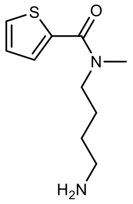

Compound **15** was synthesized according to general procedure II. The reaction was monitored over *via* TLC and the crude product was used *in situ* without further purification.

N-methyl-N-(4-((4-nitrophenyl)sulfonamido)butyl)thiophene-2-carboxamide (**16**)

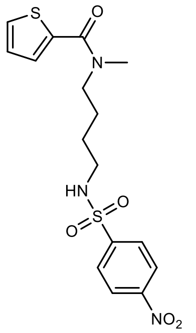

4-nitrobenzenesulfonyl chloride (135 mg, 610 µmol) was dissolved in dry DMF (5 mL). The solution was cooled to 0 °C. Then, DIPEA (320 µL, 1,83 mmol) and *N*-(4-aminobutyl)-*N*-methylthiophene-2-carboxamide (**15**) (156 mg, 732 µmol) were added in succession. The reaction solution was stirred for 16 h at ambient temperature. Afterwards, the crude product was purified *via* flash chromatography on silica using acetonitrile/water as eluent. The product was isolated as a yellow solid in a yield of 21%. **^{1}H NMR** (400 MHz, DMSO-d_6): δ = 8.46 – 8.35 (m, 2H), 8.06 – 8.01 (m, 2H), 8.00 (s, 1H), 7.73 (dd, J = 5.1, 1.1 Hz, 1H), 7.41 (s, 1H), 7.10 (dd, J = 5.0, 3.7 Hz, 1H), 3.39 (t, J = 7.2 Hz, 2H), 3.06 (s, 3H), 2.82 (t, J = 6.5 Hz, 2H), 1.53 (p, J = 7.7 Hz, 2H), 1.34 (s, 2H) ppm. **MS (ESI+)** *m/z* = 398.00 $[M+H]^+$; calc.: 398.10.

N-(4-((4-aminophenyl)sulfonamido)butyl)-N-methylthiophene-2-carboxamide (**4**) (**AL266**)

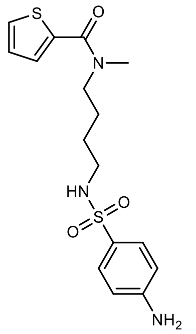

N-methyl-N-(4-((4-nitrophenyl)sulfonamido)butyl)thiophene-2-carboxamide (**16**) (45 mg, 114 µmol), ammonium chloride (43 mg, 793 µmol) and iron powder (45 mg, 793 µmol) were dissolved in methanol/water (10 mL, 9:1). The reaction solution was stirred for 3 h at 75 °C. Then, the crude solution was filtered over Celite®. The filtrate was concentrated under reduced pressure and the crude product was purified *via* flash chromatography on silica using acetonitrile/water as eluent. The product was isolated as a yellow solid in a yield of 29%. **^{1}H NMR** (500 MHz, DMSO-d_6): δ = 7.73 (d, J = 5.0 Hz, 1H), 7.56 – 7.34 (m, 3H), 7.15 – 7.06 (m, 2H), 6.60 (d, J = 8.3 Hz, 2H), 5.89 (s, 2H), 3.38 (t, J = 7.3 Hz, 2H), 3.06 (s, 3H), 2.66 (s, 2H), 1.53 (p, J = 7.3 Hz, 2H), 1.32 (s, 2H) ppm. **^{13}C{^{1}H} NMR** (126 MHz, DMSO-d_6): δ = 152.4, 128.4, 127.2, 125.6, 112.7, 42.2, 26.2 ppm. **MS (ESI+)** *m/z* = 368.20 $[M+H]^+$; calc.: 368.11.

8.2 Analytical data

^{1}H NMR and MS (ESI+) spectra of compound **6**

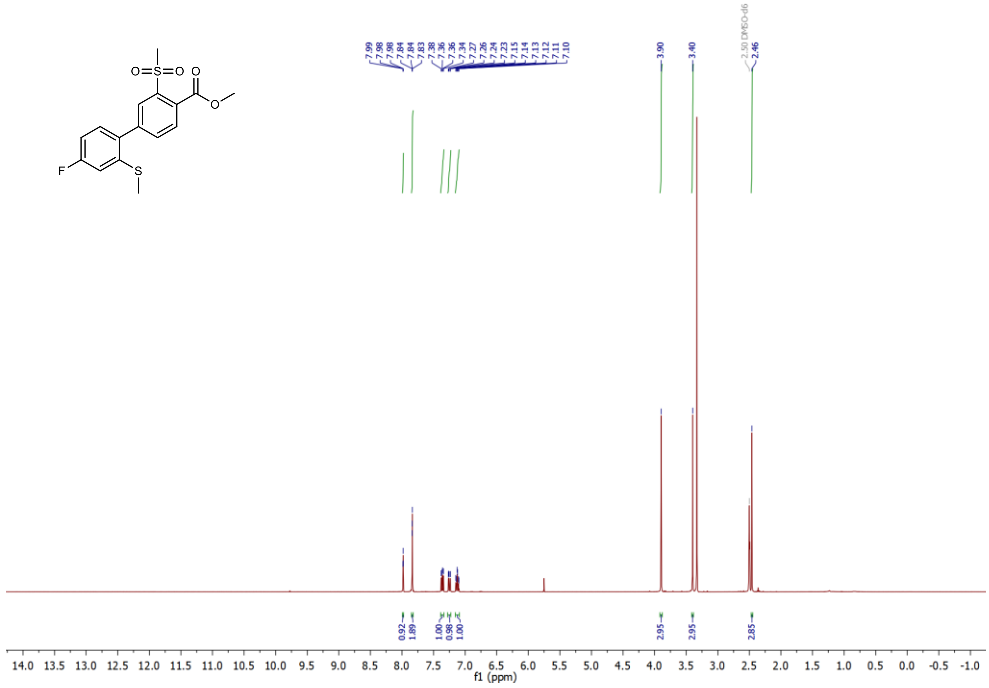

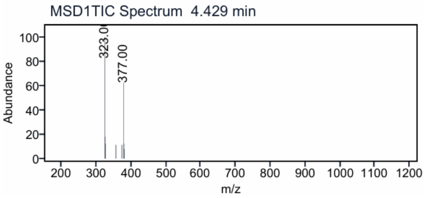

^{1}H NMR and MS (ESI+) spectra of compound **7**

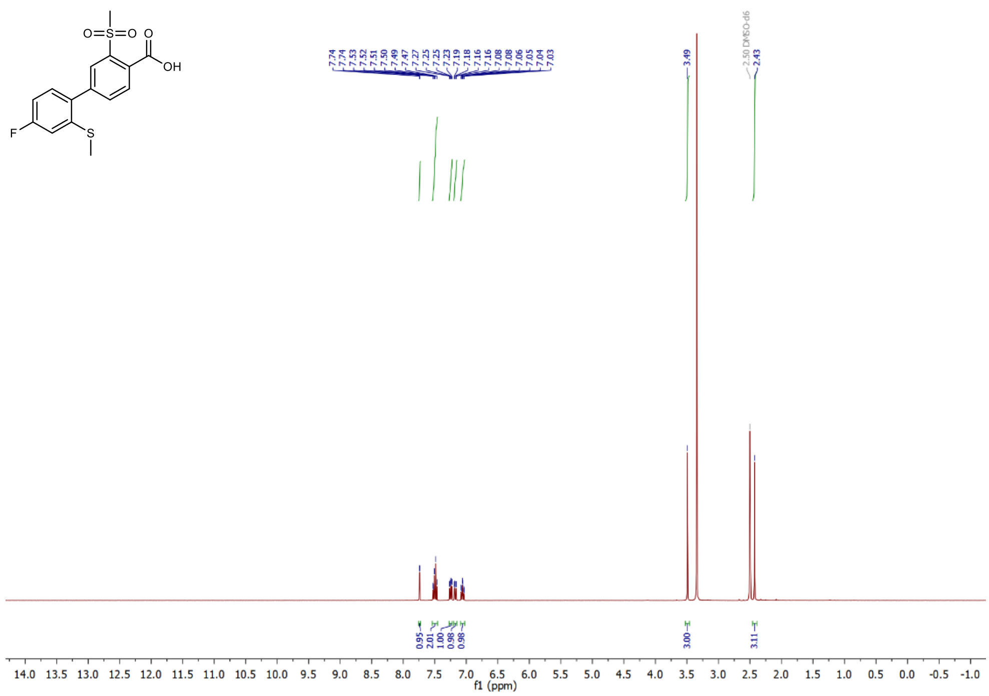

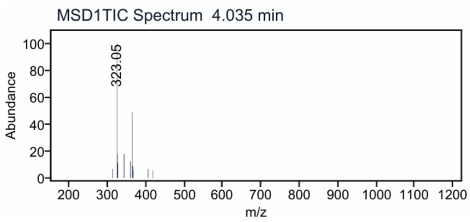

^{1}H, ^{19}F, $^{13}C\{^{1}H\}$ NMR and MS (ESI+) data of compound **1**

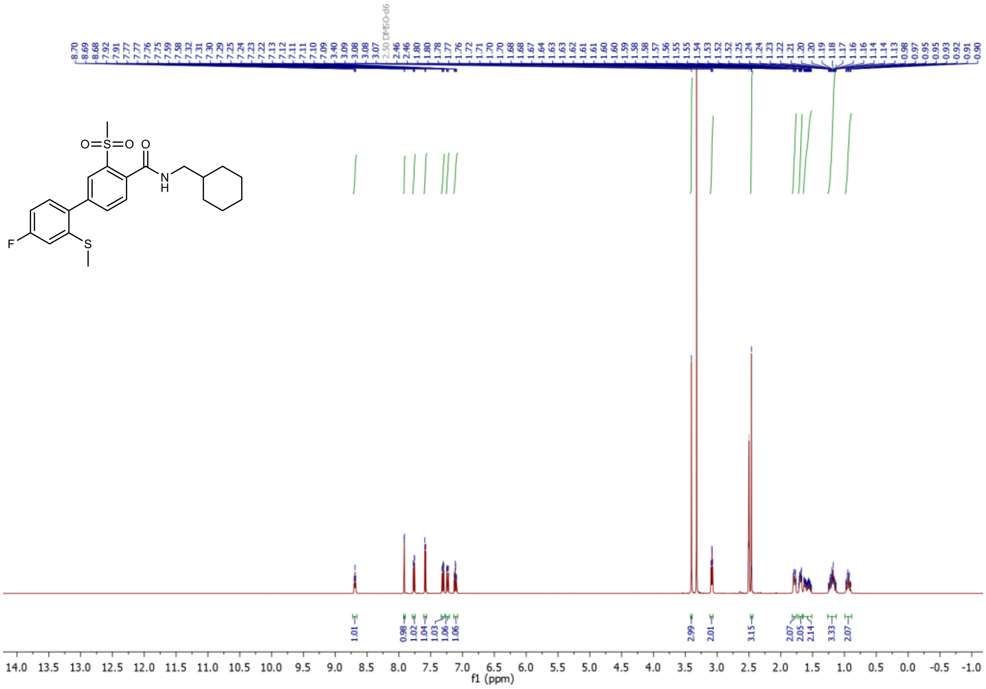

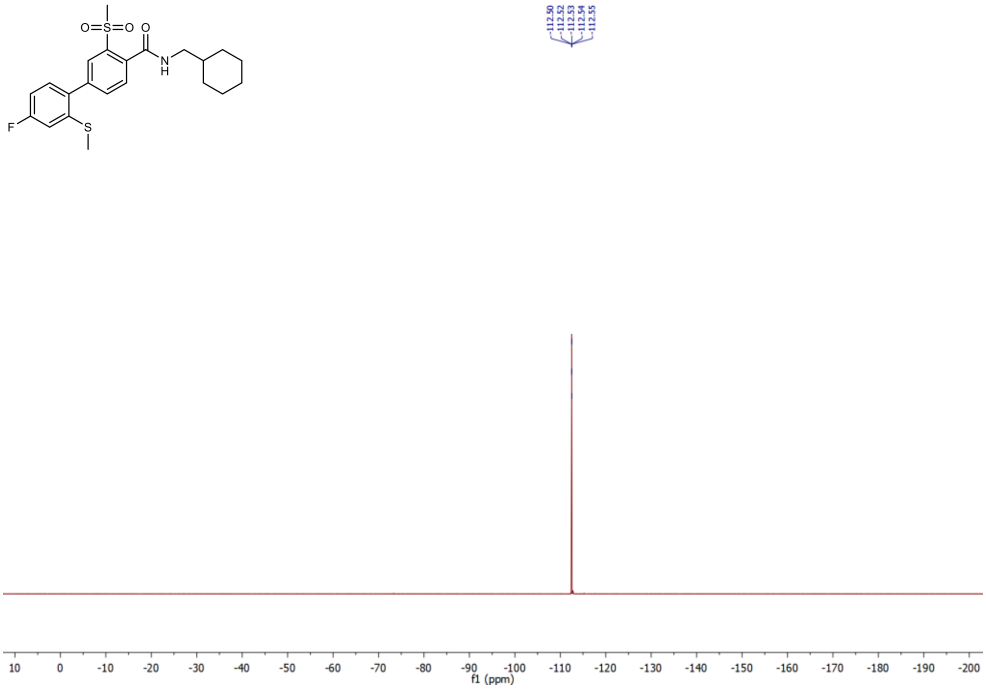

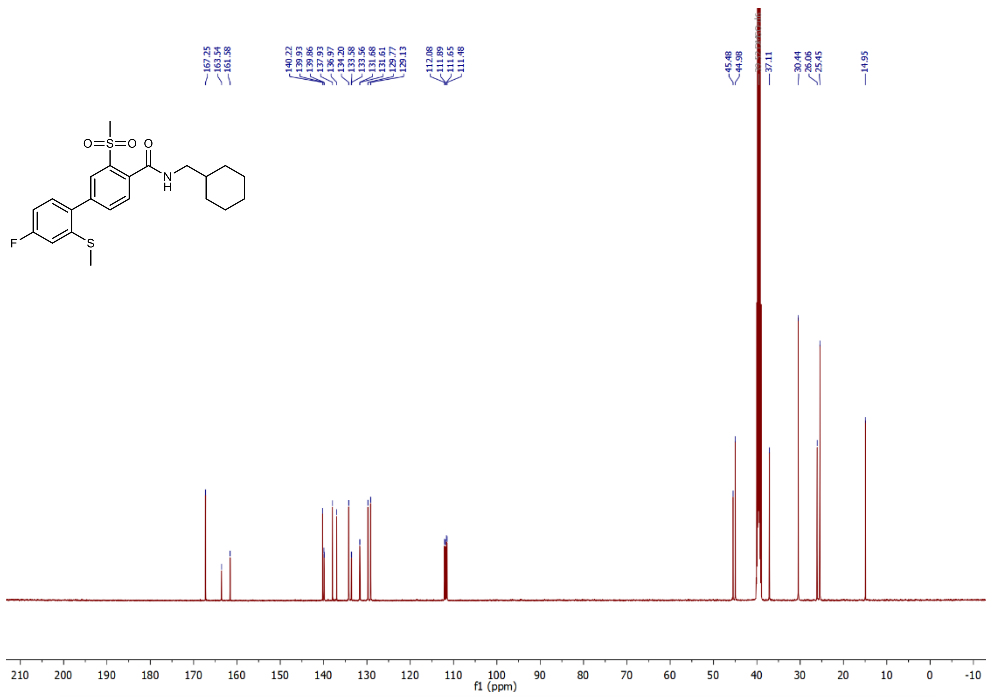

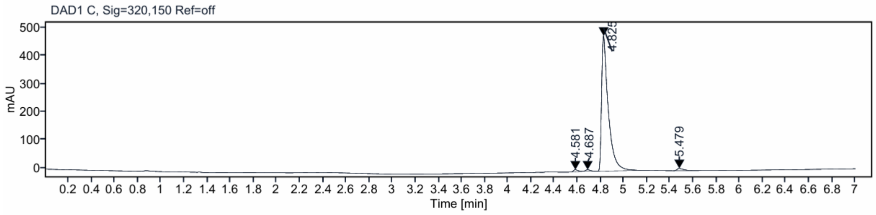

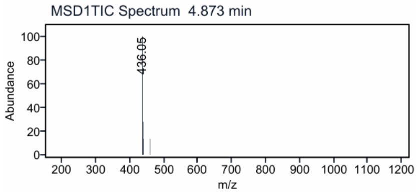

Max Area%	96.537
UV Signal Purity>95%	Pass

[1]H, ^{19}F and MS (ESI+) data of compound **8**

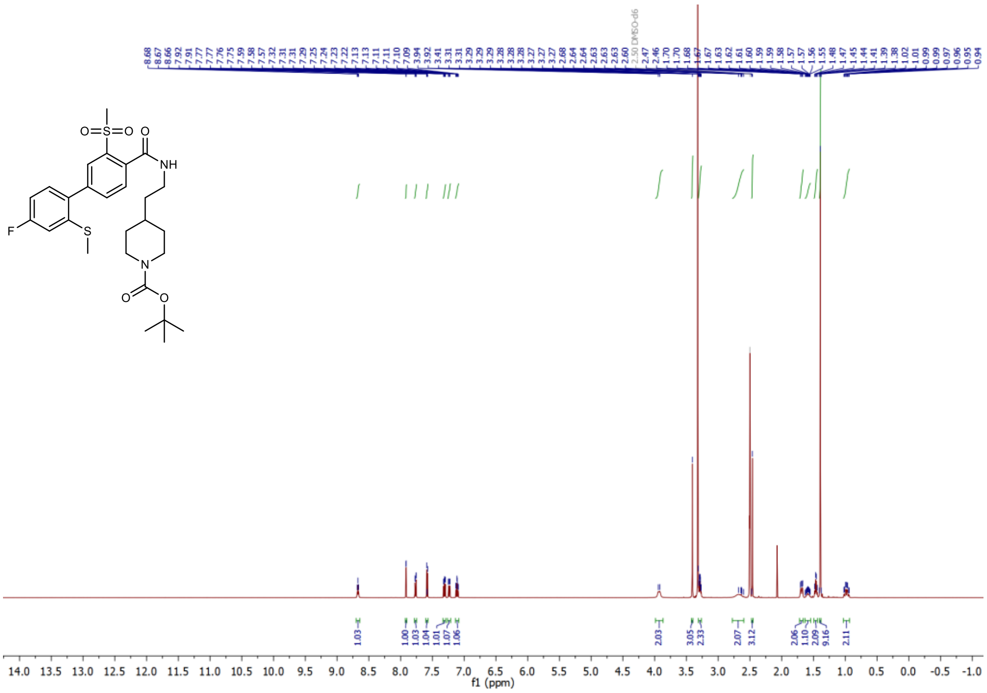

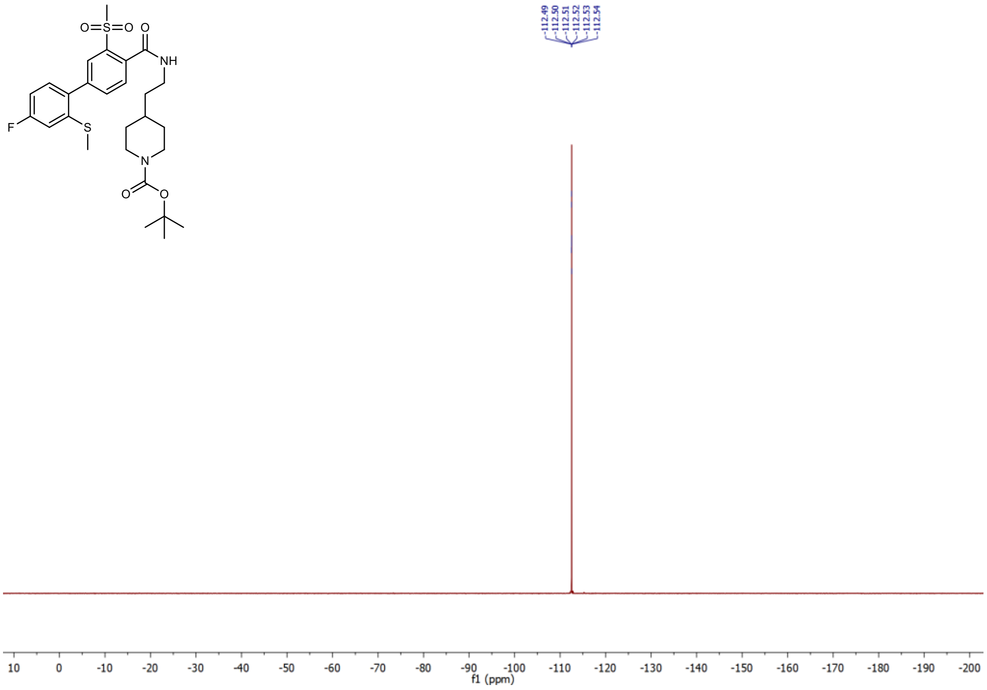

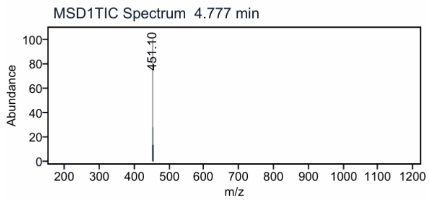

^{1}H, ^{19}F, $^{13}C\{^{1}H\}$ NMR and MS (ESI+) data of compound **9**

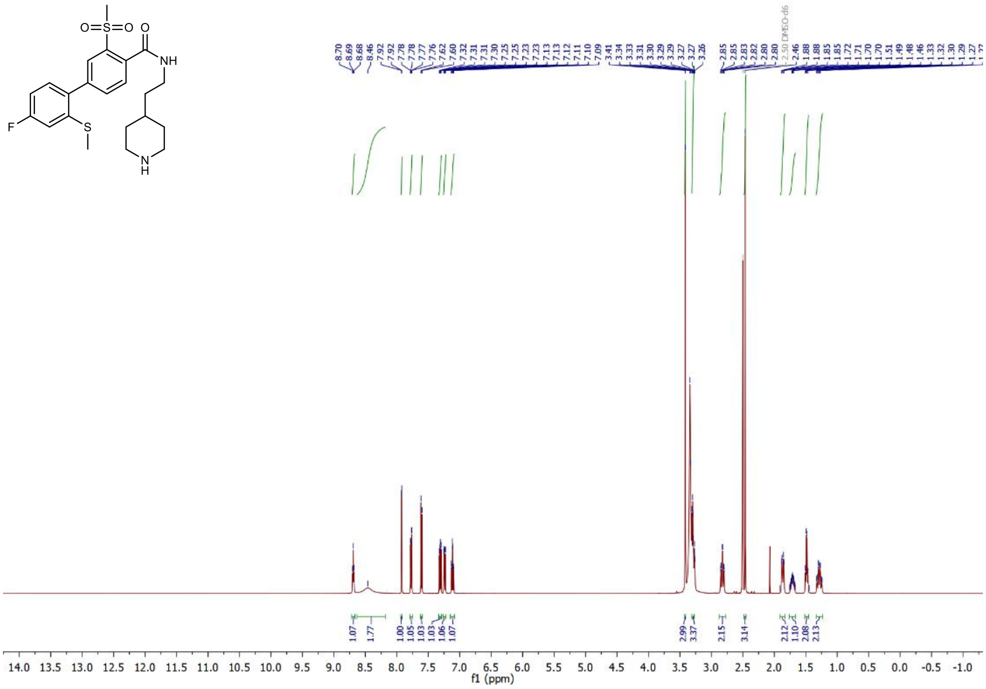

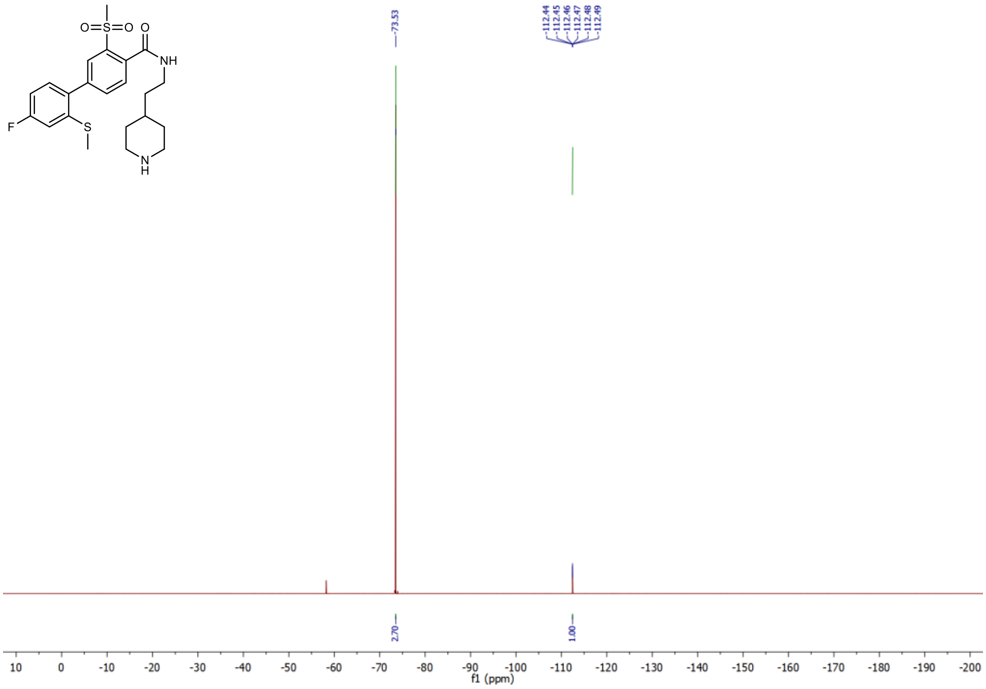

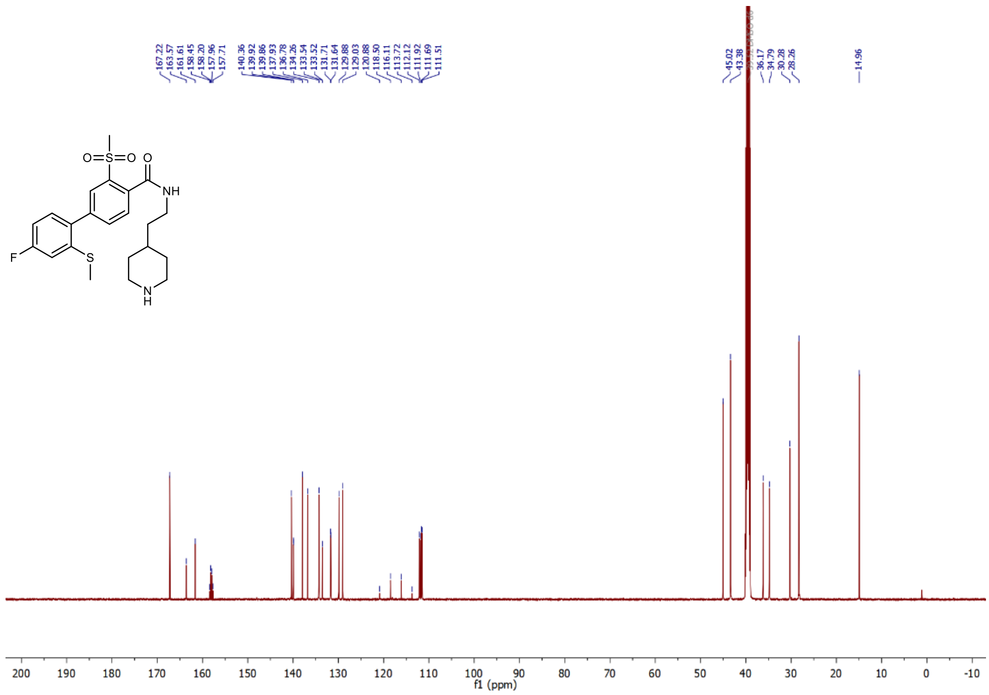

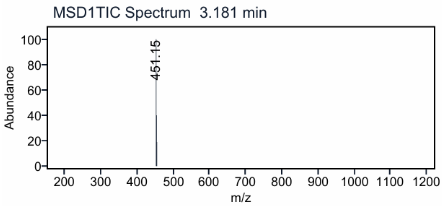
MSD1TIC Spectrum 3.181 min
451.15
Abundance
m/z

^{1}H, ^{19}F and MS (ESI+) data of compound **10**

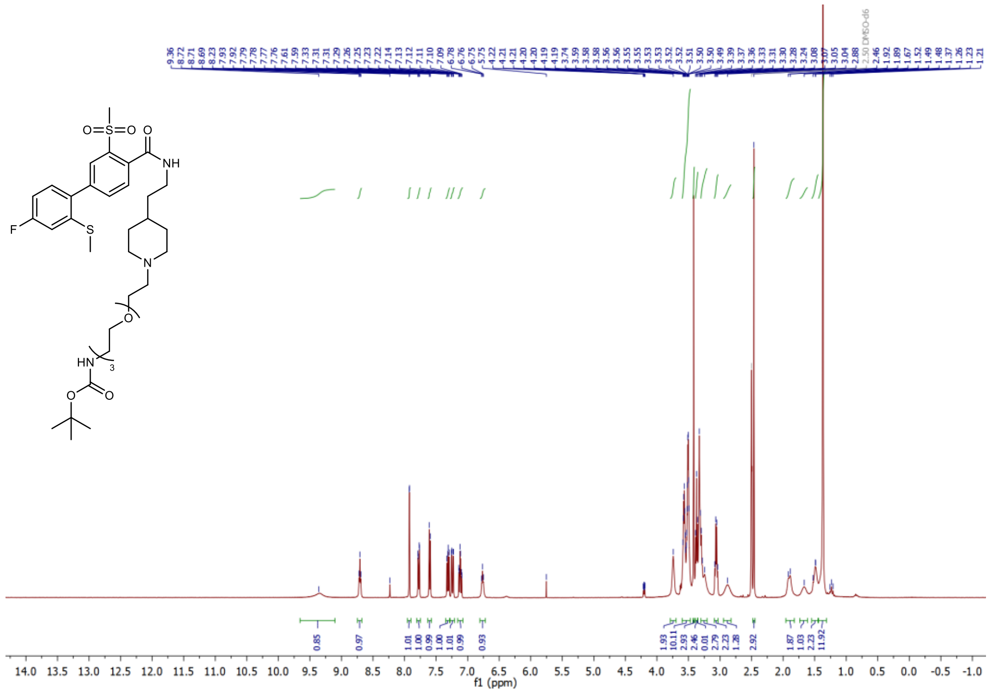

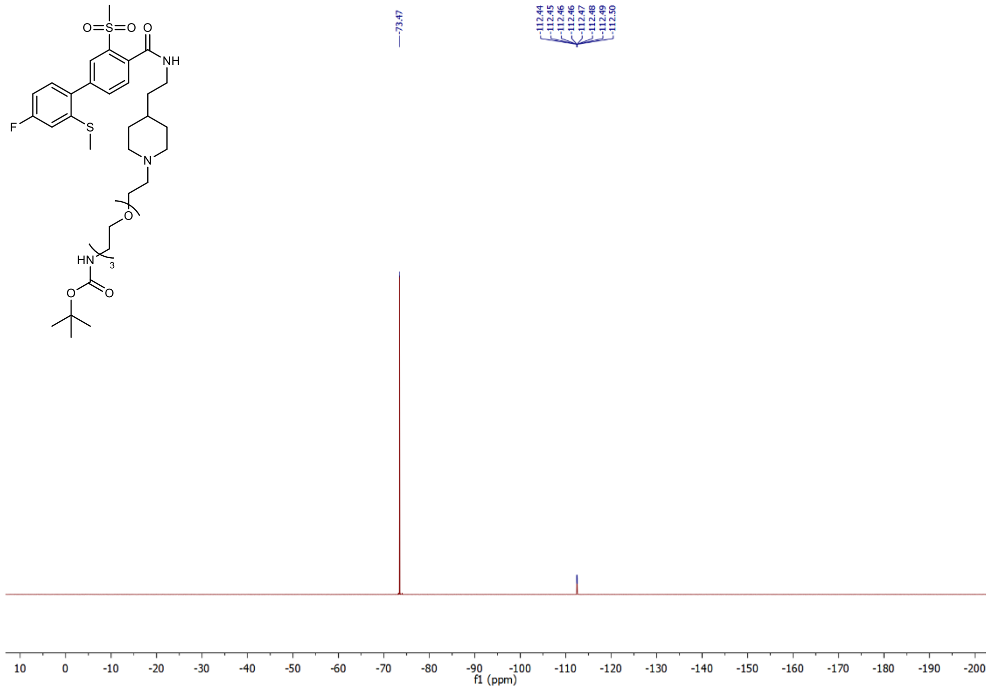

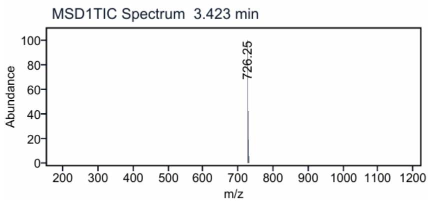

^{1}H, ^{19}F, MS (ESI+), $^{13}C\{^{1}H\}$ NMR, and HRMS (QTOF) data of compound **2**

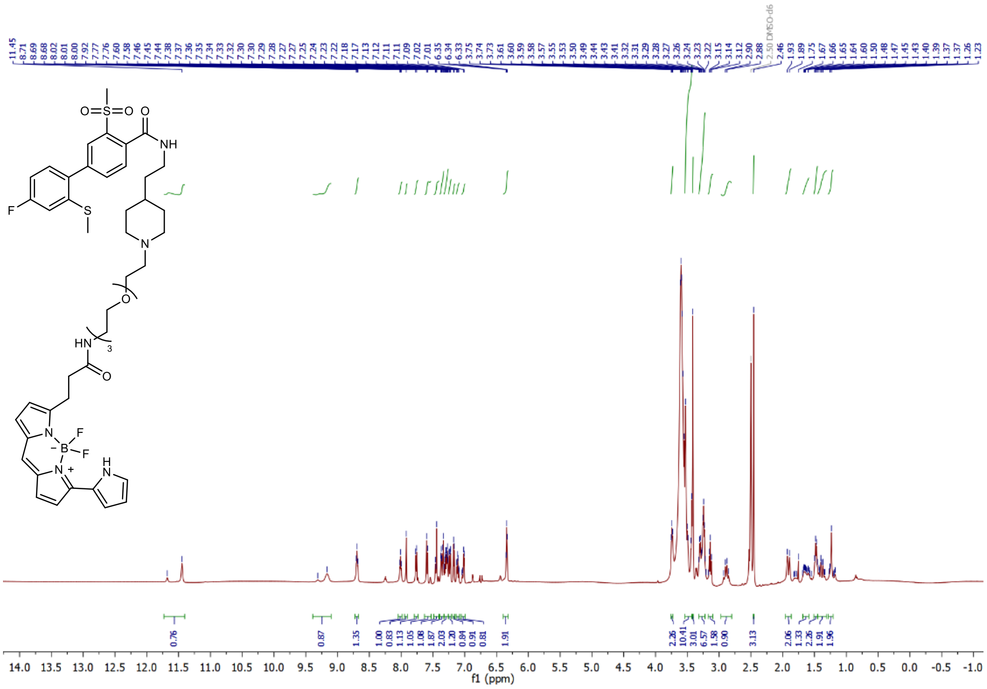

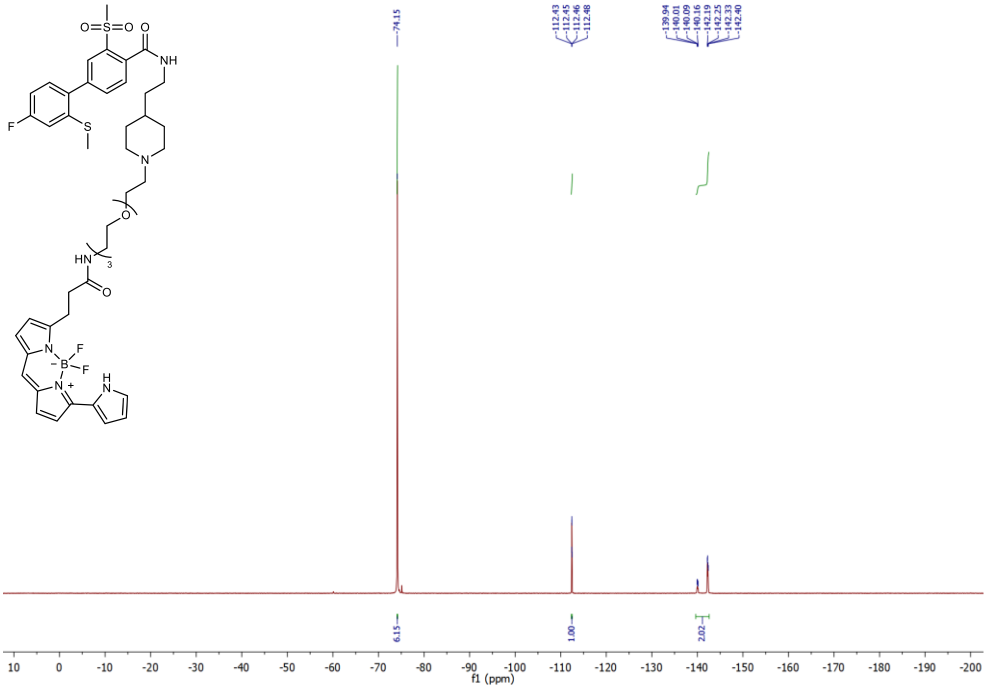

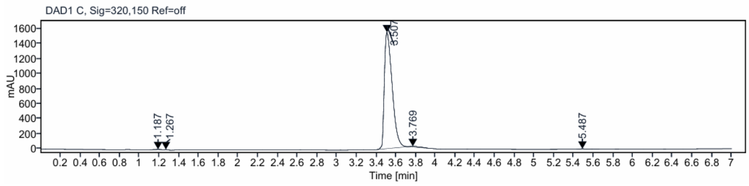

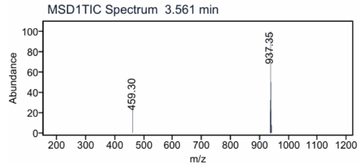

Max Area%	98.258
UV Signal Purity>95%	Pass

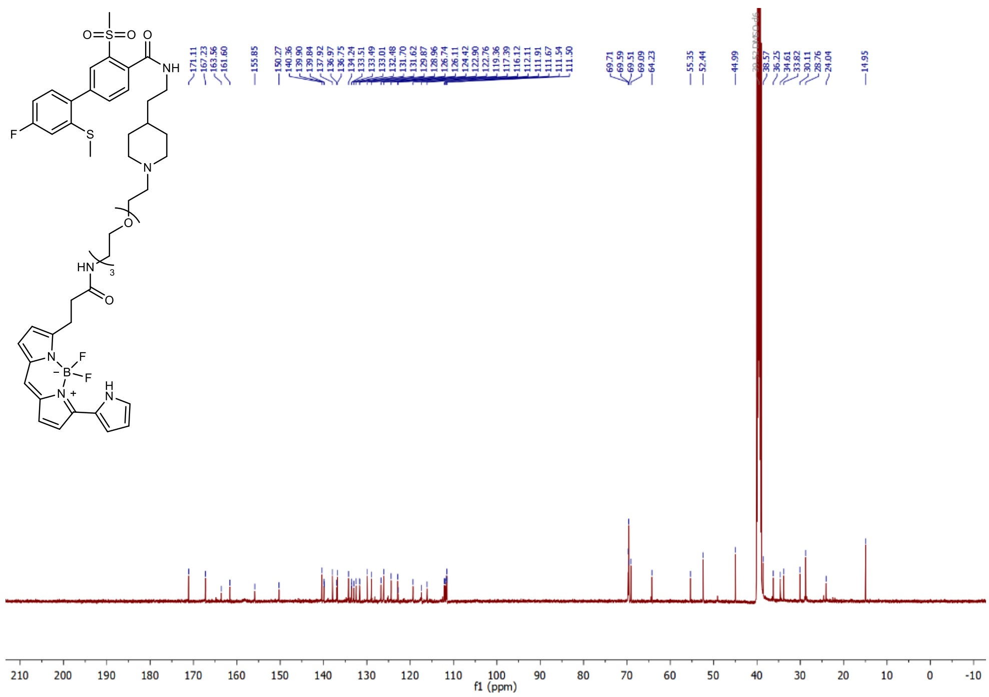
171.11
167.23
163.56
161.60
155.85
150.27
140.36
139.90
139.84
137.92
136.97
136.75
134.24
133.51
133.49
133.01
132.48
131.70
131.62
129.87
128.96
126.74
126.11
124.42
122.90
122.76
119.36
117.39
116.12
112.11
111.91
111.67
111.54
111.50
69.71
69.59
69.51
69.09
64.23
55.35
52.44
44.99
39.52 DMSO-d6
38.57
36.25
34.61
33.82
30.11
28.76
24.04
14.95
f1 (ppm)

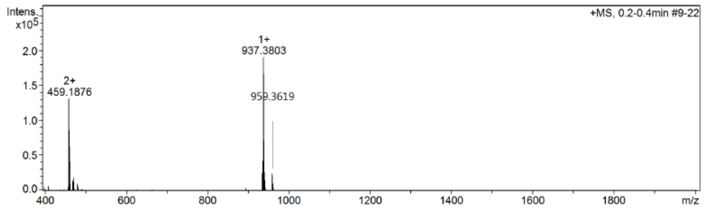
Intens. x10^5
+MS, 0.2-0.4min #9-22
2+
459.1876
1+
937.3803
959.3619
m/z

^{1}H, ^{13}C{^{1}H} NMR and MS (ESI+) data of compound **3**

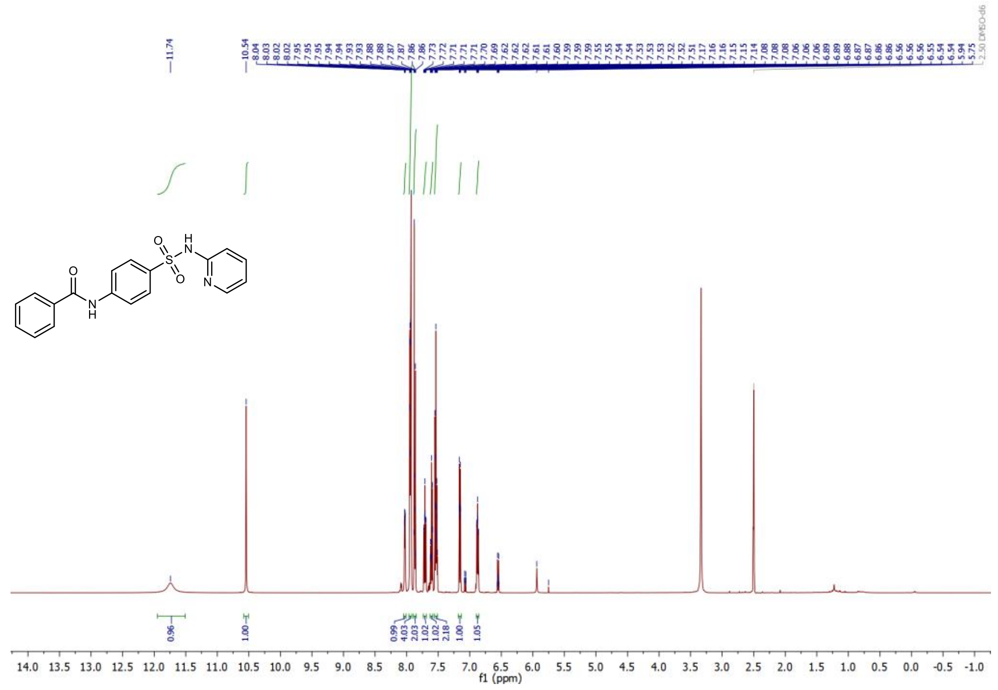

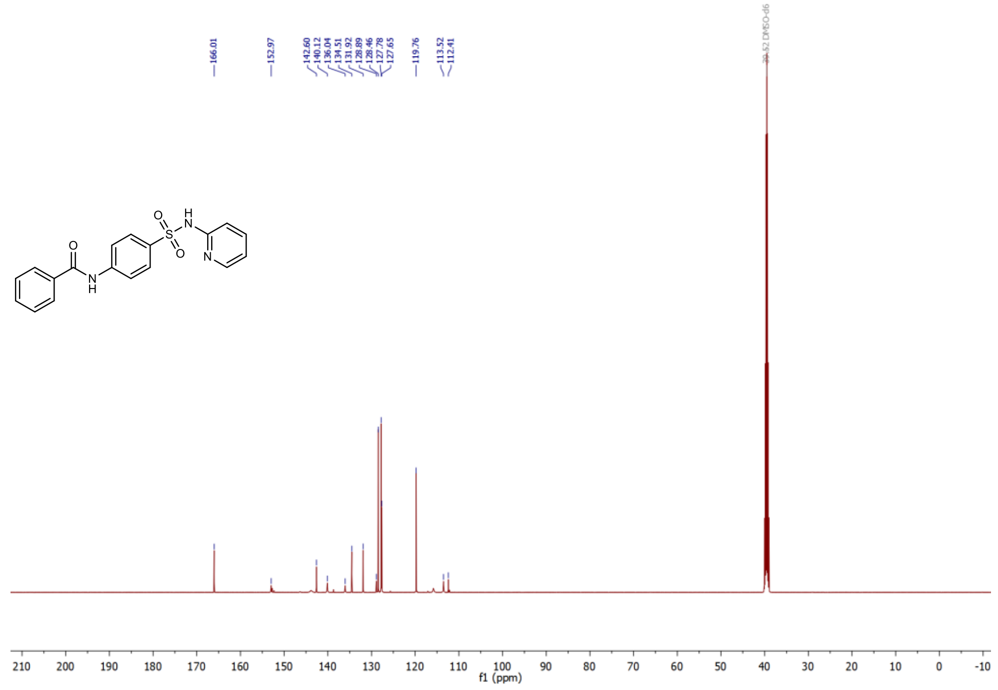

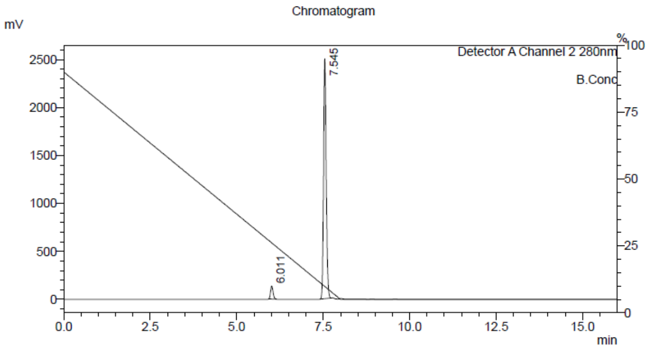

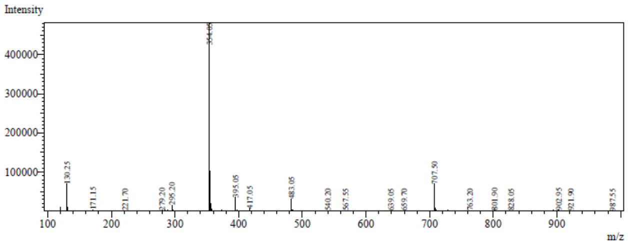

Detector A Channel 2 280nm

Peak#	Ret. Time	Area	Height	Conc.	Area%	Height%	Area/Height
1	6.011	685724	135596	4.905	4.905	5.140	5.057
2	7.545	13295694	2502464	95.095	95.095	94.860	5.313
Total		13981418	2638059		100.000	100.000	

^{1}H NMR and MS (ESI+) spectra of compound **14**

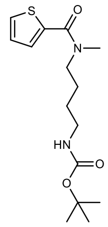

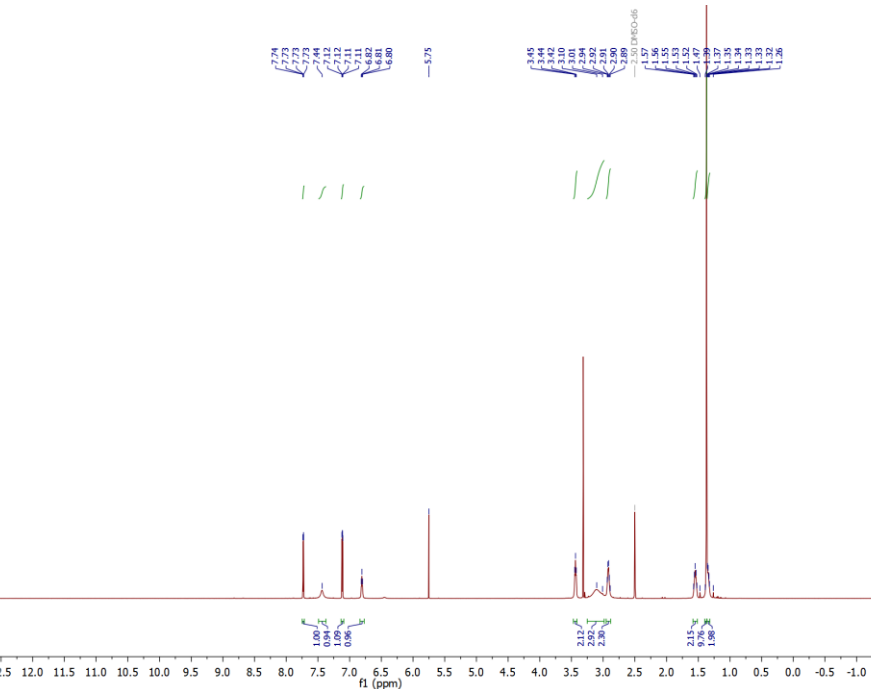

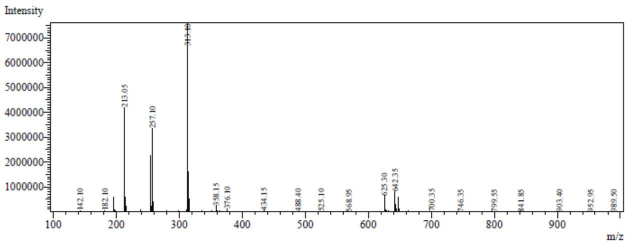

^{1}H NMR and MS (ESI+) spectra of compound **16**

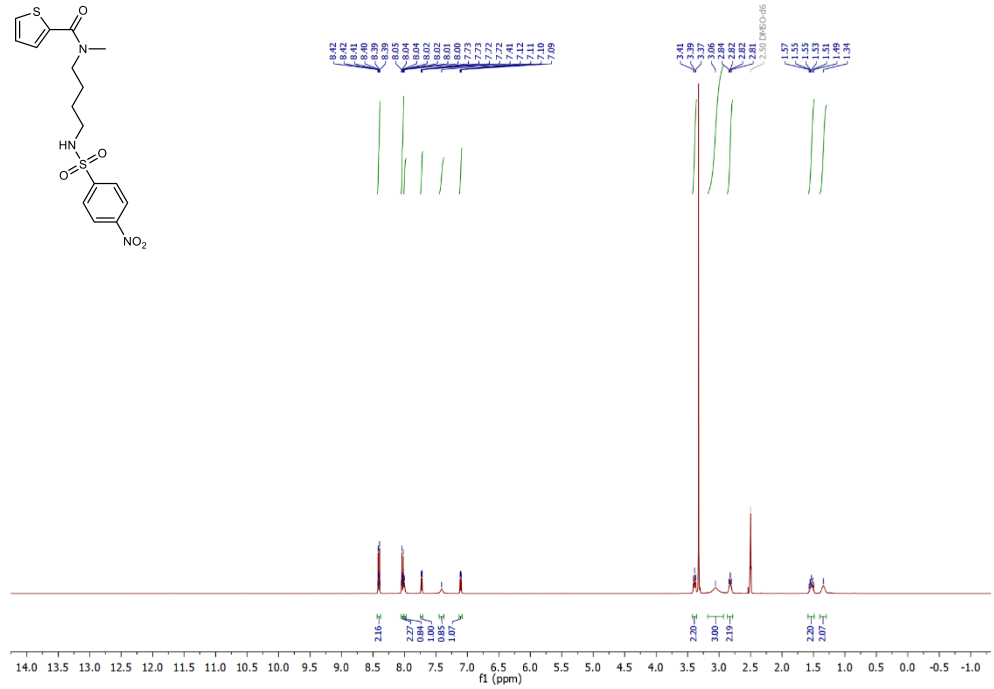

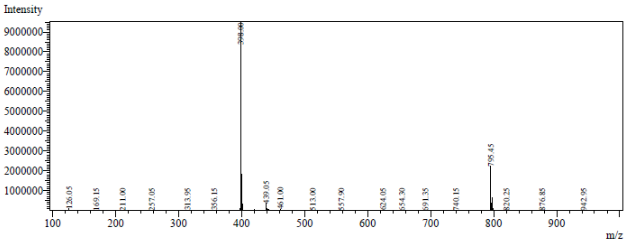

^{1}H, $^{13}C\{^{1}H\}$ NMR and MS (ESI+) data of compound **4**

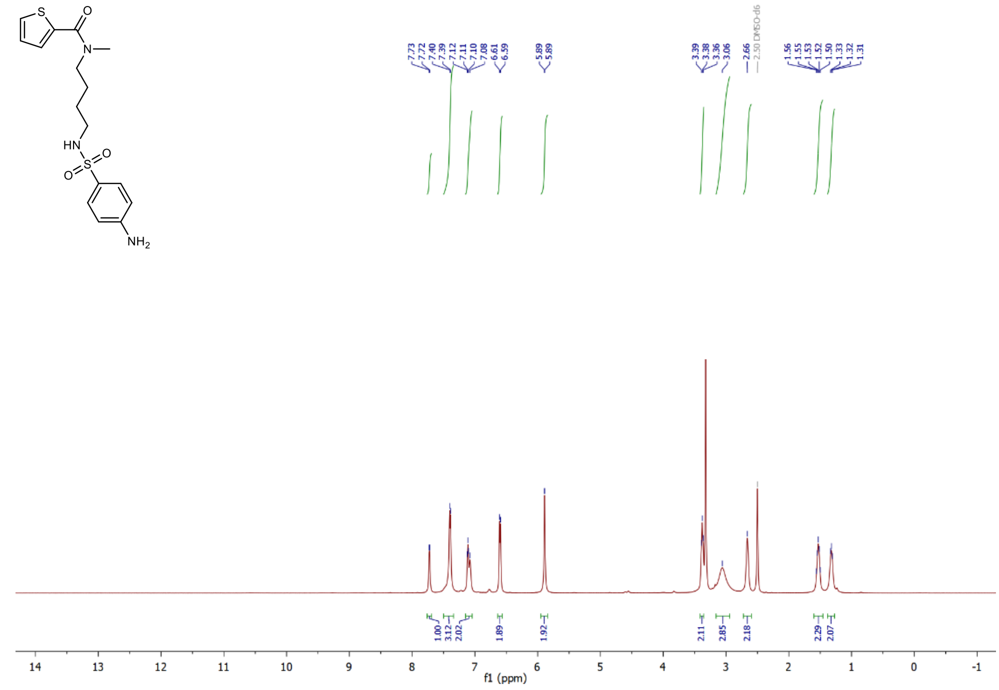

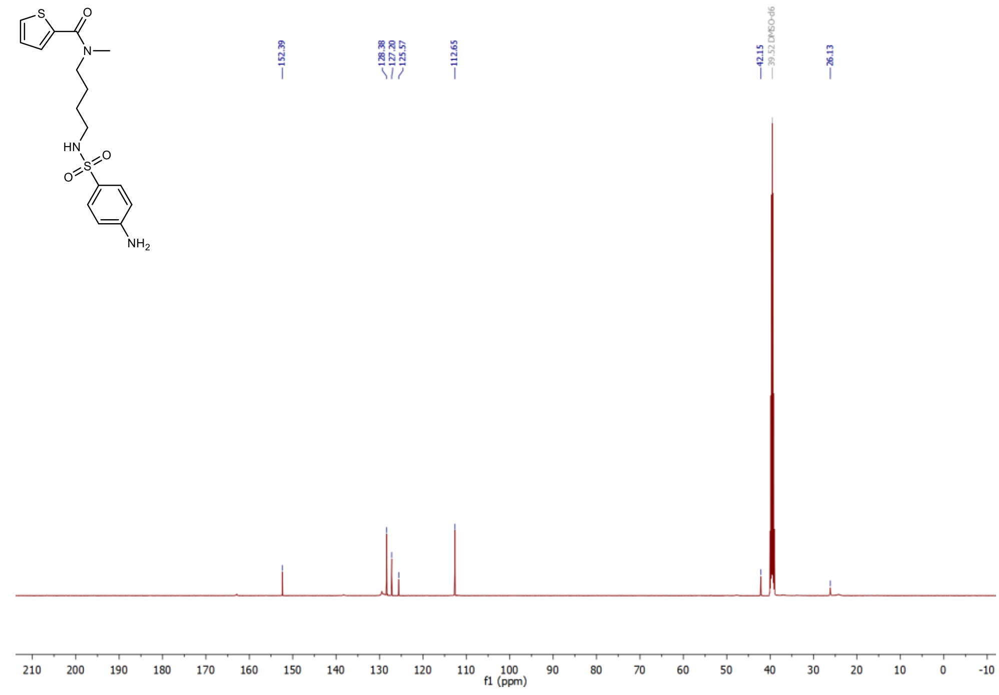

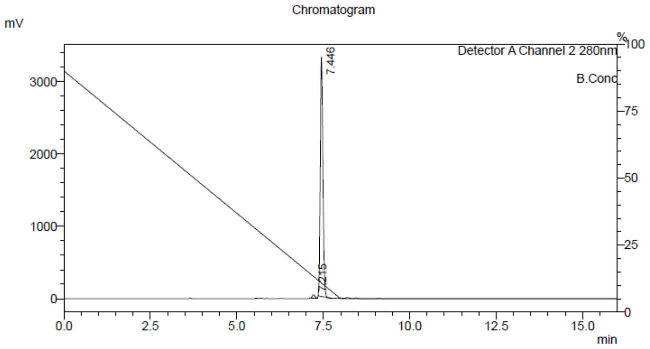

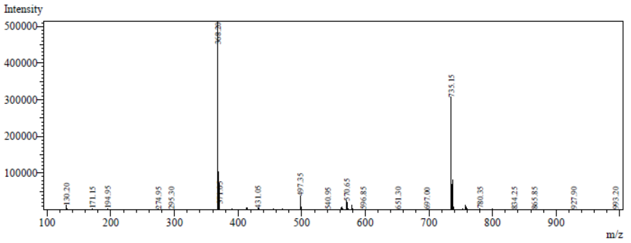

Detector A Channel 2 280nm

Peak#	Ret. Time	Area	Height	Conc.	Area%	Height%	Area/Height
1	7.215	190810	41211	1.077	1.077	1.233	4.630
2	7.446	17525396	3300465	98.923	98.923	98.767	5.310
Total		17716205	3341676		100.000	100.000	

9. Used plasmids and protein sequences.

Mouse TRIM21 construct: pET-3d vector (N-terminal His_6-tag-TRIM21 PRY-SPRY) UniProt-ID: Q62191

MHHHHHHMVHITLDRNTANSWLIISKDRRQVRMGDTHQNVSDNKERFSNYPMVLGAQRFSSGKMYWEVDVT
QKEAWDLGVCRDSVQRKGQFSLSPENGFWTIWLWQDSYEAGTSPQTTLHIQVPPCQIGIFVDYEAGVVSFYNITD
HGSLIYTFSECVFAGPLRPFFNVGFNYSGGNAAPLKLCPLKM

Human TRIM21 construct: pSUMO-Lic (N-terminal His_6-SUMO-tag-TRIM21 PRY-SPRY) UniProt-ID: P19474

MCSSHHHHHHGSGSGSDQEAKPSTEDLGDKKEGEYIKLKVIGQDSSEIHFKVKMTTHLKKLKESYCQRQGVPMNSL
RFLFEGQRIADNHTPKELGMEEEDVIEVYQEQTGGVHITLDPDTANPWLILSEDRRQVRLGDTQQSIPGNEERFDSY
PMVLGAQHFHSGKHYWEVDVTGKEAWDLGVCRDSVRRKGHFLLSSKSGFWTIWLWNKQKYEAGTYPQTPLHLQ
VPPCQVGIFLDYEAGMVSFYNITDHGSLIYSFSECAFTGPLRPFFSPGFNDGGKNTAPLTLCPL

TRIM21-NanoLuc construct: pf-32 vector

MASAARLTMMWEEVTCPICLDPFVEPVSIECGHSFCQECISQVGKGGGSVCPVCRQRFLLKNLRPNRQLANMVN
NLKEISQEAREGTQGERCAVHGERLHLFCEKDGKALCWVCAQSRKHRDHAMVPLEEAAQEYQEKLQVALGELRRK
QELAEKLEVEIAIKRADWKKTVETQKSRIHAEFVQQKNFLVEEEQRQLQELEKDEREQLRILGEKEAKLAQQSQALQE
LISELDRRCHSSALELLQEVIIVLERSESWNLKDLDITSPELRSVCHVPGLKKMLRTCAVHITLDPDTANPWLILSEDRR
QVRLGDTQQSIPGNEERFDSYPMVLGAQHFHSGKHYWEVDVTGKEAWDLGVCRDSVRRKGHFLLSSKSGFWTI
WLWNKQKYEAGTYPQTPLHLQVPPCQVGIFLDYEAGMVSFYNITDHGSLIYSFSECAFTGPLRPFFSPGFNDGGKN
TAPLTLCPLNIGSQGSTDYGSSGVFTLEDFVGDWRQTAGYNLDQVLEQGGVSSLFQNLGVSVTPIQRIVLSGENGLK
IDIHVIIPYEGLSGDQMGQIEKIFKVVYPVDDHHFKVILHYGTLVIDGVTPNMIDYFGRPYEGIAVFDGKKITVTGTLW
NGNKIIDERLINPDGSLLFRVTINGVTGWRLCERILA

10. Data Collection and Refinement Table

Table S2: Data Collection and Refinement Statistics

PDB	7HLA	7HLB	7HLC	7HLD	7HLE	7HLF
Fragment ID	Z111634612	Z45705015	Z1079512010	Z1401276297	Z1696844792	Z2092555279
Wavelength	0.9212	0.9212	0.9212	0.9212	0.9212	0.9212
Resolution range	31.28 - 1.23 (1.26 - 1.23)	31.23 - 1.21 (1.24 - 1.21)	33.91 - 1.35 (1.39 - 1.35)	33.80 - 1.22 (1.25 - 1.22)	33.92 - 1.40 (1.44 - 1.40)	31.28 - 1.39 (1.43 - 1.39)
Space group	I 4	I 4	I 4	I 4	I 4	I 4
Cell (a b c)	95.72 95.72 45.74	95.83 95.83 45.55	95.86 95.86 45.63	95.53 95.53 45.52	95.89 95.89 45.70	95.86 95.86 45.67
Cell (alpha beta gamma)	90.00 90.00 90.00	90.00 90.00 90.00	90.00 90.00 90.00	90.00 90.00 90.00	90.00 90.00 90.00	90.00 90.00 90.00
Total reflections	583818 (4322)	586709 (3221)	543224 (12868)	568389 (2797)	512150 (16858)	519108 (17033)
Unique reflections	55780 (1658)	55750 (1405)	45569 (2239)	57891 (3398)	41073 (2003)	41853 (2066)
Multiplicity	10.50 (2.60)	10.50 (2.30)	11.90 (5.70)	9.82 (3.2)	12.50 (8.40)	12.40 (8.20)
Completeness (%)	92.60 (55.90)	88.40 (43.60)	100.00 (99.60)	94.84 (54.91)	100.00 (99.50)	100.00 (99.20)
Mean I/sigma(I)	16.10 (1.06)	18.30 (1.03)	12.70 (1.01)	19.44 (1.00)	12.80 (1.03)	10.10 (0.99)
R-merge	0.064 (1.182)	0.056 (1.142)	0.086 (1.809)	0.050 (1.257)	0.100 (2.690)	0.112 (2.593)
R-rim	0.019 (0.840)	0.016 (0.843)	0.025 (0.808)	0.015 (1.524)	0.029 (0.986)	0.032 (0.953)
CC-half	0.998 (0.313)	1.000 (0.309)	0.998 (0.291)	0.999 (0.358)	0.998 (0.361)	0.999 (0.329)
R-factor	0.173 (0.353)	0.179 (0.348)	0.183 (0.383)	0.178 (0.374)	0.183 (0.368)	0.183 (0.381)
R-free	0.190 (0.336)	0.197 (0.344)	0.206 (0.393)	0.196 (0.395)	0.209 (0.369)	0.204 (0.371)
Number of total atoms	1762	1741	1734	1736	1729	1743
atoms for ligands	56	53	28	28	27	28
atoms for waters	140	141	140	142	136	149
Number of polymer residues	185	185	185	185	185	185
Average B-factor	17.5	18.3	21.9	17.7	21.7	21.5
B-factor for ligands	25.4	34.3	56.1	31.8	37.9	39.7
B-factor for solvent	28.6	29.7	33.5	28.8	32.4	36.1
RMS(bonds)	0.012	0.013	0.012	0.013	0.011	0.011
RMS(bond angles)	2.001	1.808	1.728	1.903	1.716	1.7
RMS(dihedral angles)	7.288	7.342	7.198	7.313	7.277	7.234

Values for the highest resolution shell are shown in parentheses

PDB	7HLG	7HLH	7HLI	7HLJ	7HLK	7HLL
Fragment ID	Z2365130785	Z453319206	Z2017861827	Z1980894300	Z285642082	Z1328968520
Wavelength	0.9212	0.9212	0.9212	0.9212	0.9212	0.9212
Resolution range	33.99 - 1.50 (1.54 - 1.50)	33.87 - 1.38 (1.42 - 1.38)	31.29 - 1.35 (1.39 - 1.35)	31.20 - 1.37 (1.41 - 1.37)	31.26 - 1.39 (1.43 - 1.39)	33.74 - 1.15 (1.18 - 1.15)
Space group	I 4	I 4	I 4	I 4	I 4	I 4
Cell (a b c)	96.08 96.08 45.57	95.72 95.72 45.65	95.78 95.78 45.76	95.50 95.50 45.64	95.71 95.71 45.72	95.38 95.38 45.82
Cell (alpha beta gamma)	90.00 90.00 90.00	90.00 90.00 90.00	90.00 90.00 90.00	90.00 90.00 90.00	90.00 90.00 90.00	90.00 90.00 90.00
Total reflections	444419 (17448)	525291 (16585)	541923 (13238)	528847 (15487)	517352 (18228)	585256 (446)
Unique reflections	33453 (1687)	42498 (2054)	45681 (2309)	43189 (2085)	40466 (2001)	62166 (433)
Multiplicity	13.30 (10.30)	12.40 (8.10)	11.90 (5.70)	12.20 (7.40)	12.80 (9.10)	9.40 (1.00)
Completeness (%)	100.00 (100.00)	99.80 (98.20)	100.00 (99.30)	99.60 (96.00)	96.80 (90.20)	85.20 (12.10)
Mean I/sigma(I)	10.60 (1.05)	14.80 (1.05)	11.60 (0.99)	12.80 (1.03)	11.60 (1.00)	31.70 (2.54)
R-merge	0.157 (3.705)	0.101 (2.309)	0.094 (2.711)	0.106 (2.349)	0.133 (4.682)	0.038 (0.122)
R-rim	0.044 (1.208)	0.029 (0.857)	0.027 (1.236)	0.031 (0.915)	0.038 (1.618)	0.011 (0.122)
CC-half	0.996 (0.296)	0.999 (0.407)	0.999 (0.342)	0.999 (0.302)	0.998 (0.284)	0.999 (0.927)
R-factor	0.185 (0.381)	0.176 (0.351)	0.186 (0.388)	0.181 (0.363)	0.189 (0.407)	0.170 (0.228)
R-free	0.208 (0.434)	0.195 (0.382)	0.212 (0.390)	0.198 (0.350)	0.212 (0.401)	0.188 (0.267)
Number of total atoms	1621	1760	1628	1756	1624	1609
atoms for ligands	27	47	39	43	43	24
atoms for waters	28	147	31	147	33	19
Number of polymer residues	185	185	185	185	185	185
Average B-factor	23.2	20.3	23.2	19.9	22.3	12.7
B-factor for ligands	42.1	31.6	39.2	33.2	41.4	24.9
B-factor for solvent	30.9	33.5	29.3	32.4	28.4	22.7
RMS(bonds)	0.01	0.012	0.011	0.011	0.011	0.014
RMS(bond angles)	1.645	1.77	1.748	1.701	1.676	1.987
RMS(dihedral angles)	7.293	7.422	7.355	7.351	7.185	7.378

Values for the highest resolution shell are shown in parentheses

PDB	7HLM	7HLN	7HLO	7HLP	7HLQ	7HLR
Fragment ID	Z29077827	Z26823525	Z90122368	Z29634868	Z32327641	Z1430613393
Wavelength	0.9212	0.9212	0.9212	0.9212	0.9212	0.9212
Resolution range	33.79 - 1.15 (1.18 - 1.15)	31.23 - 1.20 (1.23 - 1.20)	33.75 - 1.15 (1.18 - 1.15)	33.62 - 1.19 (1.22 - 1.19)	67.52 - 1.33 (1.36 - 1.33)	67.58 - 1.34 (1.38 - 1.34)
Space group	I 4	I 4	I 4	I 4	I 4	I 4
Cell (a b c)	95.51 95.51 45.88	95.31 95.31 45.83	95.40 95.40 45.79	95.02 95.02 45.46	95.49 95.49 45.91	95.57 95.57 45.81
Cell (alpha beta gamma)	90.00 90.00 90.00	90.00 90.00 90.00	90.00 90.00 90.00	90.00 90.00 90.00	90.00 90.00 90.00	90.00 90.00 90.00
Total reflections	588195 (441)	585684 (2587)	587100 (449)	573574 (1889)	552079 (11691)	546051 (11679)
Unique reflections	61914 (434)	56056 (1332)	62299 (436)	59389 (1288)	47560 (2392)	46080 (1922)
Multiplicity	9.50 (1.00)	10.40 (1.90)	9.40 (1.00)	9.70 (1.50)	11.60 (4.90)	11.90 (6.10)
Completeness (%)	84.30 (12.10)	87.40 (42.70)	85.40 (12.30)	91.40 (40.10)	99.90 (98.20)	99.00 (84.90)
Mean I/sigma(I)	43.00 (1.74)	14.90 (1.03)	26.00 (1.04)	14.70 (0.98)	18.00 (1.29)	12.10 (1.15)
R-merge	0.028 (0.147)	0.080 (1.020)	0.040 (0.532)	0.063 (0.844)	0.102 (1.653)	0.114 (2.010)
R-rim	0.008 (0.147)	0.024 (0.833)	0.012 (0.532)	0.018 (0.698)	0.030 (0.827)	0.033 (0.867)
CC-half	0.999 (0.947)	0.999 (0.283)	1.000 (0.622)	0.999 (0.441)	0.999 (0.379)	0.998 (0.320)
R-factor	0.177 (0.237)	0.175 (0.337)	0.176 (0.345)	0.175 (0.376)	0.177 (0.293)	0.180 (0.337)
R-free	0.192 (0.224)	0.194 (0.318)	0.190 (0.339)	0.198 (0.402)	0.200 (0.306)	0.200 (0.395)
Number of total atoms	1741	1622	1629	1645	1740	1737
atoms for ligands	26	24	25	49	31	31
atoms for waters	149	40	38	30	143	140
Number of polymer residues	185	185	185	185	185	185
Average B-factor	13.3	15	14.7	17	16.1	17.6
B-factor for ligands	27.9	31.8	31.9	26.1	32.7	32.1
B-factor for solvent	23.3	22.4	22.3	23.4	26.6	28.1
RMS(bonds)	0.014	0.012	0.013	0.012	0.013	0.012
RMS(bond angles)	1.948	1.856	1.876	1.871	1.842	1.772
RMS(dihedral angles)	7.16	7.124	7.314	7.277	7.104	7.307
Values for the highest resolution shell are shown in parentheses						

PDB	7HLV	7HLW	7HLX	7HLY	7HLZ	7HM0
Fragment ID	Z79432418	Z438096750	Z32367954	Z235449082	Z256709358	Z374427992
Wavelength	0.9212	0.9212	0.9212	0.9212	0.9212	0.9212
Resolution range	67.55 - 1.19 (1.22 - 1.19)	67.71 - 1.40 (1.44 - 1.40)	41.38 - 1.28 (1.31 - 1.28)	41.42 - 1.32 (1.35 - 1.32)	41.35 - 1.29 (1.32 - 1.29)	67.53 - 1.43 (1.47 - 1.43)
Space group	I 4	I 4	I 4	I 4	I 4	I 4
Cell (a b c)	95.53 95.53 45.70	95.75 95.75 45.86	95.40 95.40 45.88	95.47 95.47 45.93	95.43 95.43 45.83	95.50 95.50 45.81
Cell (alpha beta gamma)	90.00 90.00 90.00	90.00 90.00 90.00	90.00 90.00 90.00	90.00 90.00 90.00	90.00 90.00 90.00	90.00 90.00 90.00
Total reflections	587692 (1968)	514000 (16737)	566444 (12108)	557263 (10592)	564213 (8659)	486037 (17805)
Unique reflections	60965 (1351)	41002 (1975)	52633 (3477)	47869 (1981)	51533 (2363)	38348 (1963)
Multiplicity	9.60 (1.50)	12.50 (8.50)	10.80 (3.50)	11.60 (5.30)	10.90 (3.70)	12.70 (9.10)
Completeness (%)	92.40 (42.30)	100.00 (99.50)	98.90 (88.70)	98.40 (83.40)	99.20 (89.60)	100.00 (99.60)
Mean I/sigma(I)	20.20 (1.04)	9.90 (1.05)	19.00 (1.18)	13.60 (1.04)	16.70 (1.18)	9.70 (0.99)
R-merge	0.054 (0.761)	0.144 (2.348)	0.054 (1.242)	0.090 (1.727)	0.077 (1.107)	0.139 (5.227)
R-rim	0.016 (0.693)	0.042 (0.855)	0.016 (0.761)	0.026 (0.811)	0.023 (0.697)	0.040 (1.808)
CC-half	1.000 (0.375)	0.997 (0.405)	1.000 (0.339)	0.998 (0.333)	0.999 (0.239)	0.999 (0.353)
R-factor	0.178 (0.337)	0.184 (0.350)	0.176 (0.343)	0.181 (0.357)	0.176 (0.332)	0.188 (0.373)
R-free	0.197 (0.365)	0.216 (0.375)	0.195 (0.337)	0.199 (0.340)	0.195 (0.309)	0.214 (0.388)
Number of total atoms	1746	1725	1740	1736	1742	1738
atoms for ligands	43	24	28	30	29	23
atoms for waters	137	143	146	140	147	149
Number of polymer residues	185	185	185	185	185	185
Average B-factor	15	18.1	18.2	17.9	17	22.3
B-factor for ligands	25.1	35.9	33.7	37.2	37	46.8
B-factor for solvent	26.2	29.2	28.8	28.9	27.6	33.6
RMS(bonds)	0.012	0.01	0.012	0.011	0.013	0.011
RMS(bond angles)	1.81	1.688	1.833	1.76	1.851	1.597
RMS(dihedral angles)	7.19	7.081	7.255	7.157	7.116	7.106

Values for the highest resolution shell are shown in parentheses

PDB	7HM1	7HM2	7HM3	7HM4	7HM5	7HM6
Fragment ID	Z793778804	Z32400357	Z1359419878	Z1407673036	Z192955056	Z1172243962
Wavelength	0.9212	0.9212	0.9212	0.9212	0.9212	0.9212
Resolution range	67.49 - 1.34 (1.38 - 1.34)	67.38 - 1.15 (1.18 - 1.15)	41.37 - 1.23 (1.26 - 1.23)	41.31 - 1.42 (1.46 - 1.42)	33.88 - 1.38 (1.42 - 1.38)	67.61 - 1.33 (1.36 - 1.33)
Space group	I 4	I 4	I 4	I 4	I 4	I 4
Cell (a b c)	95.44 95.44 45.91	95.29 95.29 45.86	95.51 95.51 45.85	95.73 95.73 45.74	95.75 95.75 45.88	95.62 95.62 45.84
Cell (alpha beta gamma)	90.00 90.00 90.00	90.00 90.00 90.00	90.00 90.00 90.00	90.00 90.00 90.00	90.00 90.00 90.00	90.00 90.00 90.00
Total reflections	546293 (11899)	587265 (465)	584046 (4467)	496399 (17359)	527980 (16675)	553879 (11574)
Unique reflections	46517 (2269)	62218 (455)	55882 (1717)	39250 (1952)	42800 (2106)	47652 (2380)
Multiplicity	11.70 (5.20)	9.40 (1.00)	10.50 (2.60)	12.60 (8.90)	12.30 (7.90)	11.60 (4.90)
Completeness (%)	100.00 (99.70)	85.30 (12.80)	93.20 (58.30)	100.00 (100.00)	100.00 (100.00)	100.00 (99.40)
Mean I/sigma(I)	15.20 (1.05)	23.90 (1.13)	16.70 (1.06)	9.70 (1.01)	12.20 (1.03)	13.00 (1.08)
R-merge	0.086 (1.652)	0.048 (0.561)	0.072 (1.113)	0.139 (3.386)	0.107 (2.204)	0.111 (1.725)
R-rim	0.025 (0.794)	0.014 (0.561)	0.021 (0.799)	0.040 (1.201)	0.031 (0.832)	0.033 (0.861)
CC-half	0.999 (0.298)	0.998 (0.508)	0.999 (0.331)	0.998 (0.314)	0.999 (0.392)	0.999 (0.311)
R-factor	0.186 (0.353)	0.180 (0.332)	0.178 (0.344)	0.188 (0.363)	0.180 (0.363)	0.177 (0.339)
R-free	0.212 (0.358)	0.200 (0.325)	0.198 (0.306)	0.213 (0.365)	0.202 (0.373)	0.197 (0.336)
Number of total atoms	1620	1725	1746	1719	1628	1631
atoms for ligands	23	27	30	22	30	27
atoms for waters	31	140	150	139	32	38
Number of polymer residues	185	185	185	185	185	185
Average B-factor	18.3	15	16.1	21	20.1	16.8
B-factor for ligands	36.1	30.1	32.7	37.3	39.4	37.8
B-factor for solvent	26.4	25.8	26.7	32.5	26.9	26
RMS(bonds)	0.011	0.013	0.012	0.01	0.01	0.011
RMS(bond angles)	1.719	1.93	1.803	1.598	1.695	1.793
RMS(dihedral angles)	6.944	8.027	7.146	7.256	7.135	7.125

Values for the highest resolution shell are shown in parentheses

PDB	7HM8	7HM9	7HMA	7HMB	7HMC	7HMD
Fragment ID	Z285782452	Z1222331430	Z291279160	Z1374778753	Z1954800564	Z425387594
Wavelength	0.9212	0.9212	0.9212	0.9212	0.9212	0.9212
Resolution range	41.36 - 1.33 (1.36 - 1.33)	67.53 - 1.25 (1.28 - 1.25)	47.78 - 1.15 (1.18 - 1.15)	67.46 - 1.36 (1.40 - 1.36)	67.65 - 1.32 (1.35 - 1.32)	33.88 - 1.29 (1.32 - 1.29)
Space group	I 4	I 4	I 4	I 4	I 4	I 4
Cell (a b c)	95.64 95.64 45.82	95.50 95.50 45.69	95.47 95.47 45.71	95.41 95.41 45.85	95.67 95.67 45.89	95.77 95.77 45.51
Cell (alpha beta gamma)	90.00 90.00 90.00	90.00 90.00 90.00	90.00 90.00 90.00	90.00 90.00 90.00	90.00 90.00 90.00	90.00 90.00 90.00
Total reflections	552323 (11213)	578560 (5802)	588478 (470)	534013 (13723)	559151 (10619)	567584 (8327)
Unique reflections	47530 (2204)	50642 (1297)	61858 (448)	44412 (2164)	46840 (1660)	51602 (2282)
Multiplicity	11.60 (5.10)	11.40 (4.50)	9.50 (1.00)	12.00 (6.30)	11.90 (6.40)	11.00 (3.60)
Completeness (%)	99.50 (93.60)	89.00 (45.50)	84.60 (12.50)	100.00 (99.80)	96.00 (70.50)	99.20 (89.00)
Mean I/sigma(I)	14.80 (1.05)	16.50 (1.06)	26.40 (1.11)	11.80 (1.03)	14.80 (1.05)	18.90 (1.24)
R-merge	0.082 (1.737)	0.075 (1.460)	0.043 (0.460)	0.104 (2.478)	0.093 (1.810)	0.068 (0.982)
R-rim	0.024 (0.816)	0.022 (0.758)	0.013 (0.460)	0.030 (1.076)	0.027 (0.752)	0.020 (0.610)
CC-half	0.999 (0.375)	0.998 (0.327)	0.999 (0.471)	0.999 (0.322)	0.997 (0.317)	0.999 (0.404)
R-factor	0.180 (0.348)	0.179 (0.338)	0.176 (0.312)	0.182 (0.353)	0.181 (0.342)	0.172 (0.324)
R-free	0.199 (0.355)	0.200 (0.372)	0.189 (0.275)	0.207 (0.334)	0.205 (0.346)	0.191 (0.314)
Number of total atoms	1749	1627	1629	1600	1743	1749
atoms for ligands	40	24	28	23	37	32
atoms for waters	143	37	35	19	140	151
Number of polymer residues	185	185	185	185	185	185
Average B-factor	19.6	17.3	16.4	18.8	17.5	15.8
B-factor for ligands	57.7	34.9	38.4	37	31	25.1
B-factor for solvent	29.4	23.9	23.3	23.8	28.5	26.9
RMS(bonds)	0.012	0.012	0.013	0.011	0.011	0.013
RMS(bond angles)	1.776	1.831	1.894	1.69	1.752	1.869
RMS(dihedral angles)	7.072	7.072	7.27	7.032	7.292	7.258

Values for the highest resolution shell are shown in parentheses

PDB	7HME	7HMF	7HMG	7HMH	7HMI	7HMJ
Fragment ID	Z319545618	Z1367324110	Z274575916	Z1636723439	Z1742054999	Z1578665941
Wavelength	0.9212	0.9212	0.9213	0.9213	0.9213	0.9213
Resolution range	67.69 - 1.51 (1.55 - 1.51)	67.29 - 1.20 (1.23 - 1.20)	67.55 - 1.32 (1.35 - 1.32)	41.36 - 1.50 (1.54 - 1.50)	67.62 - 1.42 (1.46 - 1.42)	47.96 - 1.50 (1.54 - 1.50)
Space group	I 4	I 4	I 4	I 4	I 4	I 4
Cell (a b c)	95.72 95.72 45.78	95.16 95.16 45.73	95.53 95.53 45.76	95.50 95.50 45.84	95.63 95.63 45.89	95.83 95.83 45.88
Cell (alpha beta gamma)	90.00 90.00 90.00	90.00 90.00 90.00	90.00 90.00 90.00	90.00 90.00 90.00	90.00 90.00 90.00	90.00 90.00 90.00
Total reflections	434412 (17460)	581454 (2766)	556728 (10548)	438729 (26065)	499362 (17139)	445491 (17006)
Unique reflections	32650 (1662)	59941 (1727)	48506 (2346)	33253 (2472)	39275 (1929)	33483 (1631)
Multiplicity	13.30 (10.50)	9.70 (1.60)	11.50 (4.50)	13.20 (10.50)	12.70 (8.90)	13.30 (10.40)
Completeness (%)	100.00 (100.00)	93.80 (52.20)	99.90 (97.90)	100.00 (100.00)	100.00 (99.80)	100.00 (99.40)
Mean I/sigma(I)	8.80 (1.00)	18.90 (1.03)	17.80 (1.21)	10.90 (1.13)	9.60 (1.06)	10.30 (1.02)
R-merge	0.177 (4.703)	0.054 (0.851)	0.126 (2.968)	0.236 (3.049)	0.209 (4.556)	0.165 (3.587)
R-rim	0.050 (1.516)	0.016 (0.762)	0.038 (1.523)	0.067 (0.982)	0.060 (1.614)	0.047 (1.168)
CC-half	0.999 (0.287)	1.000 (0.359)	0.999 (0.305)	0.997 (0.342)	0.999 (0.370)	0.999 (0.298)
R-factor	0.184 (0.372)	0.180 (0.357)	0.173 (0.291)	0.189 (0.462)	0.185 (0.345)	0.186 (0.378)
R-free	0.204 (0.368)	0.195 (0.346)	0.184 (0.299)	0.213 (0.469)	0.215 (0.364)	0.214 (0.408)
Number of total atoms	1724	1749	1746	1734	1733	1731
atoms for ligands	28	43	31	23	23	26
atoms for waters	138	140	149	145	144	139
Number of polymer residues	185	185	185	185	185	185
Average B-factor	23.7	16.7	15.6	17.6	18.6	21.7
B-factor for ligands	43.7	31.1	29	33.2	36.5	43.3
B-factor for solvent	34.3	27.1	25.9	28	29.2	33
RMS(bonds)	0.01	0.012	0.013	0.011	0.01	0.01
RMS(bond angles)	1.586	1.797	1.87	1.668	1.648	1.601
RMS(dihedral angles)	7.101	7.097	7.407	6.967	7.317	7.278

Values for the highest resolution shell are shown in parentheses

PDB	7HMK	7HML	7HMM	7HMN	7HMO	7HMP
Fragment ID	Z271004858	Z1354416068	Z165170770	Z57478994	Z198194394	Z1530301542
Wavelength	0.9212	0.9212	0.9212	0.9212	0.9212	0.9212
Resolution range	41.30 - 1.35 (1.39 - 1.35)	33.79 - 1.26 (1.29 - 1.26)	67.56 - 1.28 (1.31 - 1.28)	33.75 - 1.33 (1.36 - 1.33)	67.63 - 1.30 (1.33 - 1.30)	41.34 - 1.15 (1.18 - 1.15)
Space group	I 4	I 4	I 4	I 4	I 4	I 4
Cell (a b c)	95.60 95.60 45.74	95.52 95.52 45.69	95.55 95.55 45.79	95.39 95.39 45.70	95.65 95.65 45.81	95.43 95.43 45.82
Cell (alpha beta gamma)	90.00 90.00 90.00	90.00 90.00 90.00	90.00 90.00 90.00	90.00 90.00 90.00	90.00 90.00 90.00	90.00 90.00 90.00
Total reflections	542695 (14219)	575184 (6503)	571923 (7725)	547600 (11176)	566849 (9337)	589226 (323)
Unique reflections	44379 (1990)	54726 (2330)	52550 (2255)	47236 (2274)	45987 (1202)	62342 (321)
Multiplicity	12.20 (7.10)	10.50 (2.80)	10.90 (3.40)	11.60 (4.90)	12.30 (7.80)	9.50 (1.00)
Completeness (%)	97.70 (84.50)	98.10 (81.00)	98.70 (86.60)	99.90 (97.60)	90.20 (48.40)	84.30 (8.80)
Mean I/sigma(I)	13.00 (1.07)	21.10 (1.03)	16.90 (1.06)	13.30 (0.84)	13.70 (1.09)	27.90 (1.09)
R-merge	0.100 (1.956)	0.052 (1.039)	0.071 (1.283)	0.085 (1.524)	0.106 (2.097)	0.039 (0.602)
R-rim	0.029 (0.755)	0.016 (0.744)	0.021 (0.811)	0.025 (0.748)	0.031 (0.789)	0.012 (0.602)
CC-half	0.998 (0.279)	1.000 (0.356)	0.999 (0.289)	0.999 (0.337)	0.998 (0.396)	0.999 (0.389)
R-factor	0.183 (0.356)	0.182 (0.353)	0.181 (0.348)	0.182 (0.367)	0.180 (0.339)	0.176 (0.316)
R-free	0.209 (0.344)	0.201 (0.382)	0.199 (0.360)	0.202 (0.404)	0.201 (0.325)	0.192 (0.302)
Number of total atoms	1739	1628	1733	1744	1734	1760
atoms for ligands	30	26	23	37	29	52
atoms for waters	143	36	144	141	139	142
Number of polymer residues	185	185	185	185	185	185
Average B-factor	18.8	18.5	17.5	19.5	17.4	14.7
B-factor for ligands	33.9	37.4	40	41.2	31.3	25.6
B-factor for solvent	30.1	26.3	28.4	29.9	28.1	24.4
RMS(bonds)	0.011	0.013	0.013	0.012	0.011	0.013
RMS(bond angles)	1.721	1.897	1.837	1.745	1.748	1.901
RMS(dihedral angles)	7.125	7.086	7.676	6.947	7.027	7.291

Values for the highest resolution shell are shown in parentheses

PDB	7HMQ	7HMR	7HMS	7HMT	7HMU	7HMV
Fragment ID	Z1267773633	Z1267882044	Z126932614	Z2004563941	Z1741966151	Z56978034
Wavelength	0.9212	0.9212	0.9212	0.9212	0.9212	0.9212
Resolution range	31.28 - 1.43 (1.47 - 1.43)	41.32 - 1.15 (1.18 - 1.15)	67.49 - 1.15 (1.18 - 1.15)	67.66 - 1.28 (1.31 - 1.28)	67.44 - 1.15 (1.18 - 1.15)	33.74 - 1.15 (1.18 - 1.15)
Space group	I 4	I 4	I 4	I 4	I 4	I 4
Cell (a b c)	95.48 95.48 45.90	95.42 95.42 45.79	95.44 95.44 45.86	95.69 95.69 45.75	95.38 95.38 45.81	95.37 95.37 45.82
Cell (alpha beta gamma)	90.00 90.00 90.00	90.00 90.00 90.00	90.00 90.00 90.00	90.00 90.00 90.00	90.00 90.00 90.00	90.00 90.00 90.00
Total reflections	490982 (25929)	589970 (320)	590480 (335)	573429 (7718)	589284 (443)	587897 (303)
Unique reflections	38341 (2808)	61624 (311)	62571 (328)	48980 (1371)	60819 (419)	57034 (281)
Multiplicity	12.80 (9.20)	9.60 (1.00)	9.40 (1.00)	11.70 (5.60)	9.70 (1.10)	10.30 (1.10)
Completeness (%)	100.00 (100.00)	83.10 (8.50)	84.70 (9.20)	91.90 (52.40)	83.40 (11.80)	77.20 (7.80)
Mean I/sigma(I)	12.10 (1.15)	27.90 (1.24)	26.50 (1.04)	12.00 (1.04)	25.90 (1.13)	16.40 (1.01)
R-merge	0.116 (2.764)	0.042 (0.317)	0.041 (0.348)	0.110 (1.889)	0.044 (0.576)	0.053 (1.078)
R-rim	0.033 (0.954)	0.012 (0.317)	0.012 (0.348)	0.032 (0.850)	0.013 (0.576)	0.016 (1.078)
CC-half	0.999 (0.330)	0.999 (0.345)	0.999 (0.320)	0.997 (0.298)	1.000 (0.439)	0.999 (0.372)
R-factor	0.179 (0.350)	0.176 (0.300)	0.178 (0.317)	0.185 (0.343)	0.175 (0.305)	0.177 (0.345)
R-free	0.203 (0.358)	0.192 (0.266)	0.194 (0.391)	0.206 (0.390)	0.189 (0.275)	0.194 (0.344)
Number of total atoms	1731	1749	1727	1729	1733	1731
atoms for ligands	19	37	23	22	21	21
atoms for waters	146	146	146	141	146	144
Number of polymer residues	185	185	185	185	185	185
Average B-factor	19.2	14.5	14.5	15.2	13.8	14.9
B-factor for ligands	38	42.8	29.9	25.8	32.4	28.7
B-factor for solvent	30.4	24.1	25.2	25.3	23.9	25.2
RMS(bonds)	0.011	0.014	0.013	0.011	0.013	0.013
RMS(bond angles)	1.729	1.898	1.906	2.01	1.869	1.839
RMS(dihedral angles)	7.033	7.15	7.328	8.465	7.184	7.175

Values for the highest resolution shell are shown in parentheses

PDB	7HMW	7HMX	7HMY	7HMZ	7HN0	7HN1
Fragment ID	Z1492796719	Z763030030	Z111782404	Z85893853	Z1217960891	Z2065616520
Wavelength	0.9212	0.9212	0.9212	0.9212	0.9212	0.9212
Resolution range	67.57 - 1.34 (1.38 - 1.34)	33.82 - 1.57 (1.61 - 1.57)	67.49 - 1.15 (1.18 - 1.15)	67.57 - 1.28 (1.31 - 1.28)	41.41 - 1.33 (1.36 - 1.33)	33.77 - 1.47 (1.51 - 1.47)
Space group	I 4	I 4	I 4	I 4	I 4	I 4
Cell (a b c)	95.56 95.56 45.93	95.58 95.58 45.94	95.45 95.45 45.85	95.56 95.56 45.88	95.42 95.42 45.92	95.45 95.45 45.78
Cell (alpha beta gamma)	90.00 90.00 90.00	90.00 90.00 90.00	90.00 90.00 90.00	90.00 90.00 90.00	90.00 90.00 90.00	90.00 90.00 90.00
Total reflections	548202 (11889)	395942 (16778)	590123 (475)	573384 (7537)	552340 (11436)	441665 (23176)
Unique reflections	46596 (2264)	29079 (1425)	56879 (419)	52832 (2277)	46750 (1971)	35195 (2576)
Multiplicity	11.80 (5.30)	13.60 (11.80)	10.40 (1.10)	10.90 (3.30)	11.80 (5.80)	12.50 (9.00)
Completeness (%)	100.00 (99.30)	100.00 (99.30)	77.70 (11.70)	98.90 (87.10)	98.40 (84.20)	100.00 (100.00)
Mean I/sigma(I)	13.40 (1.06)	3.50 (0.95)	29.90 (1.46)	14.80 (1.03)	15.40 (1.05)	8.30 (1.18)
R-merge	0.111 (1.989)	0.356 (3.228)	0.042 (0.382)	0.081 (1.203)	0.084 (1.620)	0.226 (3.199)
R-rim	0.032 (0.940)	0.100 (0.977)	0.012 (0.380)	0.024 (0.759)	0.024 (0.710)	0.064 (1.107)
CC-half	0.999 (0.318)	0.983 (0.265)	0.999 (0.592)	0.998 (0.291)	0.999 (0.356)	0.997 (0.378)
R-factor	0.186 (0.363)	0.202 (0.350)	0.173 (0.294)	0.179 (0.358)	0.181 (0.361)	0.195 (0.359)
R-free	0.207 (0.324)	0.239 (0.369)	0.188 (0.289)	0.199 (0.361)	0.204 (0.375)	0.229 (0.354)
Number of total atoms	1735	1725	1741	1735	1624	1734
atoms for ligands	23	25	31	32	24	28
atoms for waters	146	134	144	144	34	140
Number of polymer residues	185	185	185	184	185	185
Average B-factor	16.8	22.1	15	16.4	17.8	21.7
B-factor for ligands	29.9	39.9	32	32.9	36	53
B-factor for solvent	28.4	32.2	25.5	27.2	25.4	31.2
RMS(bonds)	0.012	0.007	0.013	0.012	0.011	0.01
RMS(bond angles)	1.714	1.434	1.901	1.813	1.741	1.564
RMS(dihedral angles)	6.896	6.984	7.251	7.046	6.993	7.035

Values for the highest resolution shell are shown in parentheses

PDB	7HN2	7HN3	7HN4	7HN5	7HN6	7HN7
Fragment ID	Z1002247062	Z2301438417	Z56827661	Z45617795	Z1998104358	Z198194396
Wavelength	0.9212	0.9212	0.9212	0.9213	0.9213	0.9213
Resolution range	41.29 - 1.23 (1.26 - 1.23)	31.20 - 1.28 (1.31 - 1.28)	41.29 - 1.19 (1.22 - 1.19)	67.40 - 1.32 (1.35 - 1.32)	67.71 - 1.40 (1.44 - 1.40)	67.50 - 1.31 (1.34 - 1.31)
Space group	I 4	I 4	I 4	I 4	I 4	I 4
Cell (a b c)	95.52 95.52 45.75	95.32 95.32 45.72	95.39 95.39 45.75	95.32 95.32 45.59	95.75 95.75 45.60	95.46 95.46 45.89
Cell (alpha beta gamma)	90.00 90.00 90.00	90.00 90.00 90.00	90.00 90.00 90.00	90.00 90.00 90.00	90.00 90.00 90.00	90.00 90.00 90.00
Total reflections	583990 (4437)	568224 (12269)	586822 (2022)	549456 (10488)	511561 (16972)	561227 (10296)
Unique reflections	57340 (1901)	51319 (3051)	60873 (1389)	47626 (2064)	40788 (1996)	46620 (1500)
Multiplicity	10.20 (2.30)	11.10 (4.00)	9.60 (1.50)	11.50 (5.10)	12.50 (8.50)	12.00 (6.90)
Completeness (%)	95.60 (63.60)	97.00 (77.80)	92.50 (43.60)	98.80 (87.00)	100.00 (100.00)	93.90 (60.60)
Mean I/sigma(I)	17.80 (1.02)	19.10 (1.23)	19.10 (1.08)	15.70 (1.08)	11.10 (1.03)	15.60 (1.10)
R-merge	0.063 (1.018)	0.056 (1.189)	0.056 (0.696)	0.078 (1.561)	0.122 (2.358)	0.104 (2.290)
R-rim	0.019 (0.802)	0.016 (0.652)	0.017 (0.635)	0.023 (0.756)	0.035 (0.855)	0.030 (0.925)
CC-half	0.999 (0.358)	1.000 (0.357)	1.000 (0.448)	0.998 (0.298)	0.997 (0.386)	0.999 (0.314)
R-factor	0.177 (0.345)	0.176 (0.362)	0.177 (0.344)	0.176 (0.331)	0.180 (0.363)	0.180 (0.313)
R-free	0.198 (0.344)	0.198 (0.339)	0.195 (0.304)	0.194 (0.358)	0.212 (0.351)	0.203 (0.301)
Number of total atoms	1753	1751	1737	1724	1739	1737
atoms for ligands	42	40	27	22	39	29
atoms for waters	145	145	144	144	142	142
Number of polymer residues	185	185	185	185	185	185
Average B-factor	16.6	17.6	15.5	17.8	19.2	16.9
B-factor for ligands	37.1	35.7	41.1	33.5	37.2	34.6
B-factor for solvent	27	27.7	25.6	29.1	29.9	27.7
RMS(bonds)	0.013	0.012	0.013	0.013	0.011	0.011
RMS(bond angles)	1.872	1.835	1.874	1.885	1.69	1.783
RMS(dihedral angles)	7.008	7.192	7.196	7.174	6.983	7.033

Values for the highest resolution shell are shown in parentheses

PDB	7HN8	7HN9	7HNA	7HNB	7HNC	7HND
Fragment ID	Z1318110042	Z1787761777	Z1929757385	Z31478538	Z220996120	Z52314092
Wavelength	0.9213	0.9213	0.9213	0.9213	0.9213	0.9213
Resolution range	41.28 - 1.23 (1.26 - 1.23)	67.40 - 1.22 (1.25 - 1.22)	41.37 - 1.29 (1.32 - 1.29)	47.80 - 1.52 (1.56 - 1.52)	41.43 - 1.15 (1.18 - 1.15)	33.75 - 1.34 (1.37 - 1.34)
Space group	I 4	I 4	I 4	I 4	I 4	I 4
Cell (a b c)	95.40 95.40 45.75	95.32 95.32 45.81	95.42 95.42 45.87	95.51 95.51 45.90	95.40 95.40 45.95	95.39 95.39 45.88
Cell (alpha beta gamma)	90.00 90.00 90.00	90.00 90.00 90.00	90.00 90.00 90.00	90.00 90.00 90.00	90.00 90.00 90.00	90.00 90.00 90.00
Total reflections	580633 (4384)	583026 (3668)	567038 (8135)	431204 (52050)	590390 (448)	546720 (11805)
Unique reflections	57173 (1875)	56423 (1594)	51603 (2234)	32043 (4617)	62235 (426)	46407 (2225)
Multiplicity	10.20 (2.30)	10.30 (2.30)	11.00 (3.60)	13.50 (11.30)	9.50 (1.10)	11.80 (5.30)
Completeness (%)	95.60 (63.10)	92.30 (53.20)	99.10 (87.80)	100.00 (100.00)	85.10 (12.00)	99.90 (98.00)
Mean I/sigma(I)	21.70 (1.08)	19.20 (1.05)	17.30 (1.10)	8.90 (1.52)	27.60 (1.04)	12.70 (1.04)
R-merge	0.053 (0.993)	0.063 (1.027)	0.080 (1.417)	0.175 (3.289)	0.038 (0.416)	0.116 (2.020)
R-rim	0.016 (0.773)	0.019 (0.780)	0.024 (0.834)	0.049 (1.023)	0.011 (0.416)	0.034 (0.960)
CC-half	1.000 (0.340)	0.998 (0.350)	0.999 (0.326)	0.998 (0.407)	1.000 (0.557)	0.998 (0.382)
R-factor	0.178 (0.344)	0.177 (0.339)	0.179 (0.346)	0.181 (0.329)	0.181 (0.339)	0.183 (0.346)
R-free	0.197 (0.370)	0.195 (0.333)	0.196 (0.317)	0.208 (0.328)	0.203 (0.266)	0.204 (0.355)
Number of total atoms	1745	1736	1738	1720	1702	1729
atoms for ligands	37	24	28	22	23	22
atoms for waters	142	146	144	140	141	141
Number of polymer residues	185	185	185	185	185	185
Average B-factor	15.7	15.3	15.7	20.3	15	16.7
B-factor for ligands	25.9	30.5	30.6	36.1	28.8	31.2
B-factor for solvent	26.5	25.6	25.9	31.7	25.6	27.4
RMS(bonds)	0.013	0.012	0.013	0.01	0.013	0.011
RMS(bond angles)	1.897	1.791	1.91	1.611	1.941	1.702
RMS(dihedral angles)	7.196	7.011	7.12	7.068	7.352	7.09

Values for the highest resolution shell are shown in parentheses

PDB	7HNE	7HNF	7HNG	7HNH	7HNI	7HNJ
Fragment ID	Z1343518214	Z1689442171	Z1568344634	Z2643472210	Z373768900	Z1162778919
Wavelength	0.9213	0.9213	0.9213	0.9213	0.9213	0.9213
Resolution range	67.45 - 1.33 (1.36 - 1.33)	67.50 - 1.32 (1.35 - 1.32)	41.40 - 1.34 (1.38 - 1.34)	67.57 - 1.34 (1.38 - 1.34)	41.36 - 1.27 (1.30 - 1.27)	67.44 - 1.43 (1.47 - 1.43)
Space group	I 4	I 4	I 4	I 4	I 4	I 4
Cell (a b c)	95.39 95.39 45.86	95.46 95.46 45.73	95.44 95.44 45.90	95.56 95.56 45.80	95.32 95.32 45.86	95.37 95.37 45.82
Cell (alpha beta gamma)	90.00 90.00 90.00	90.00 90.00 90.00	90.00 90.00 90.00	90.00 90.00 90.00	90.00 90.00 90.00	90.00 90.00 90.00
Total reflections	551574 (11539)	554419 (10646)	547453 (12521)	547085 (11812)	572959 (6897)	488344 (17226)
Unique reflections	46243 (1844)	48226 (2280)	45816 (2054)	46437 (2217)	51186 (1663)	38158 (1891)
Multiplicity	11.90 (6.30)	11.50 (4.70)	11.90 (6.10)	11.80 (5.30)	11.20 (4.10)	12.80 (9.10)
Completeness (%)	97.50 (78.90)	99.60 (95.30)	98.70 (88.00)	99.90 (98.80)	94.40 (63.60)	100.00 (100.00)
Mean I/sigma(I)	18.20 (1.36)	17.60 (1.30)	9.80 (1.08)	14.70 (1.08)	17.30 (1.09	13.80 (1.14)
R-merge	0.096 (1.308)	0.094 (1.926)	0.122 (1.635)	0.094 (1.759)	0.083 (1.566)	0.278 (9.999)
R-rim	0.028 (0.575)	0.028 (0.980)	0.035 (0.726)	0.027 (0.839)	0.025 (0.849)	0.081 (3.727)
CC-half	0.996 (0.332)	0.999 (0.264)	0.998 (0.277)	0.999 (0.319)	0.997 (0.313)	0.997 (0.356)
R-factor	0.177 (0.283)	0.176 (0.299)	0.176 (0.325)	0.176 (0.345)	0.173 (0.327)	0.169 (0.302)
R-free	0.198 (0.306)	0.202 (0.309)	0.202 (0.363)	0.194 (0.383)	0.190 (0.300)	0.196 (0.324)
Number of total atoms	1732	1749	1734	1753	1750	1736
atoms for ligands	23	39	23	43	42	39
atoms for waters	143	144	145	144	142	139
Number of polymer residues	185	185	185	185	185	185
Average B-factor	15.2	17.1	17.2	17.8	16.4	20.2
B-factor for ligands	31.8	37.7	30.5	36	34.2	37
B-factor for solvent	25.4	27.6	28.8	28.5	27	31.6
RMS(bonds)	0.013	0.012	0.011	0.011	0.013	0.011
RMS(bond angles)	1.876	1.865	1.689	1.817	1.899	1.754
RMS(dihedral angles)	7.041	7.25	7.027	7.124	7.079	7.151

Values for the highest resolution shell are shown in parentheses

PDB	7HNK	7HNL	7HNM	7HNN	7HNO	7HNP
Fragment ID	Z1275599911	Z1251207602	Z768399682	Z31432226	Z57450788	Z106579662
Wavelength	0.9213	0.9213	0.9213	0.9213	0.9213	0.9213
Resolution range	41.39 - 1.39 (1.43 - 1.39)	33.73 - 1.44 (1.48 - 1.44)	47.78 - 1.49 (1.53 - 1.49)	33.78 - 1.31 (1.34 - 1.31)	67.39 - 1.33 (1.36 - 1.33)	67.30 - 1.30 (1.33 - 1.30)
Space group	I 4	I 4	I 4	I 4	I 4	I 4
Cell (a b c)	95.57 95.57 45.88	95.33 95.33 45.76	95.47 95.47 45.76	95.48 95.48 45.90	95.31 95.31 45.66	95.18 95.18 45.85
Cell (alpha beta gamma)	90.00 90.00 90.00	90.00 90.00 90.00	90.00 90.00 90.00	90.00 90.00 90.00	90.00 90.00 90.00	90.00 90.00 90.00
Total reflections	517738 (16911)	477372 (16894)	447119 (52646)	561139 (9839)	547516 (11298)	560966 (9093)
Unique reflections	41764 (2067)	37327 (1828)	33608 (4888)	49694 (2369)	47087 (2312)	49465 (1990)
Multiplicity	12.40 (8.20)	12.80 (9.20)	13.30 (10.80)	11.30 (4.20)	11.60 (4.90)	11.30 (4.60)
Completeness (%)	100.00 (100.00)	100.00 (99.80)	100.00 (100.00)	99.90 (97.70)	99.90 (98.00)	97.90 (81.20)
Mean I/sigma(I)	10.70 (1.07)	8.10 (0.99)	11.30 (1.49)	13.40 (1.04)	13.90 (1.05)	17.80 (1.04)
R-merge	0.130 (2.108)	0.148 (3.394)	0.156 (3.574)	0.084 (1.540)	0.096 (1.707)	0.066 (1.589)
R-rim	0.037 (0.782)	0.042 (1.176)	0.044 (1.119)	0.025 (0.861)	0.028 (0.838)	0.019 (0.809)
CC-half	0.995 (0.436)	0.997 (0.379)	0.999 (0.337)	0.999 (0.266)	0.999 (0.313)	1.000 (0.300)
R-factor	0.181 (0.327)	0.190 (0.358)	0.181 (0.340)	0.178 (0.350)	0.172 (0.338)	0.176 (0.348)
R-free	0.205 (0.320)	0.216 (0.355)	0.209 (0.360)	0.202 (0.335)	0.189 (0.349)	0.199 (0.354)
Number of total atoms	1746	1747	1730	1708	1740	1734
atoms for ligands	39	40	24	27	34	29
atoms for waters	141	141	140	143	140	139
Number of polymer residues	185	185	185	185	185	185
Average B-factor	19.7	22.1	20.4	18.1	18.3	19
B-factor for ligands	31.1	49.5	39	31.4	28.9	31
B-factor for solvent	31.4	31.4	30.8	29.8	29.9	30.6
RMS(bonds)	0.011	0.009	0.01	0.011	0.013	0.012
RMS(bond angles)	1.745	1.583	1.633	1.829	1.796	1.841
RMS(dihedral angles)	6.993	7.228	7.031	7.285	7.209	7.197

Values for the highest resolution shell are shown in parentheses

PDB	**7HNQ**	**7HNR**	**7HNS**	**7HNT**	**7HNU**	**7HNV**
Fragment ID	Z30620520	Z1545312521	Z2072621991	Z383202616	Z1614545742	Z404993336
Wavelength	0.9213	0.9213	0.9213	0.9213	0.9213	0.9213
Resolution range	41.28 - 1.37 (1.41 - 1.37)	67.43 - 1.30 (1.33 - 1.30)	67.48 - 1.29 (1.32 - 1.29)	67.55 - 1.17 (1.20 - 1.17)	67.58 - 1.15 (1.18 - 1.15)	41.28 - 1.34 (1.38 - 1.34)
Space group	I 4	I 4	I 4	I 4	I 4	I 4
Cell (a b c)	95.39 95.39 45.74	95.36 95.36 45.77	95.43 95.43 45.84	95.54 95.54 45.87	95.57 95.57 45.79	95.42 95.42 45.73
Cell (alpha beta gamma)	90.00 90.00 90.00	90.00 90.00 90.00	90.00 90.00 90.00	90.00 90.00 90.00	90.00 90.00 90.00	90.00 90.00 90.00
Total reflections	528335 (15331)	563024 (9280)	567249 (8300)	589758 (1054)	586228 (468)	545867 (11964)
Unique reflections	43189 (2076)	50408 (2363)	50969 (2073)	59229 (884)	61775 (455)	46288 (2252)
Multiplicity	12.20 (7.40)	11.20 (3.90)	11.10 (4.00)	10.00 (1.20)	9.50 (1.00)	11.80 (5.30)
Completeness (%)	99.70 (97.30)	99.50 (93.40)	98.20 (82.90)	85.00 (25.90)	84.20 (12.20)	99.90 (98.80)
Mean I/sigma(I)	13.70 (1.05)	15.50 (1.04)	17.80 (1.07)	15.20 (1.05)	23.50 (0.97)	20.10 (1.58)
R-merge	0.110 (2.401)	0.080 (1.466)	0.072 (1.285)	0.057 (0.977)	0.043 (0.320)	0.093 (1.472)
R-rim	0.032 (0.936)	0.024 (0.834)	0.021 (0.740)	0.017 (0.950)	0.013 (0.320)	0.027 (0.716)
CC-half	0.999 (0.338)	0.999 (0.284)	0.999 (0.338)	0.999 (0.400)	1.000 (0.845)	0.999 (0.307)
R-factor	0.179 (0.347)	0.179 (0.359)	0.178 (0.333)	0.180 (0.341)	0.184 (0.366)	0.171 (0.281)
R-free	0.196 (0.323)	0.202 (0.397)	0.201 (0.324)	0.197 (0.348)	0.202 (0.372)	0.191 (0.275)
Number of total atoms	1752	1723	1750	1735	1736	1738
atoms for ligands	46	25	43	26	26	28
atoms for waters	140	140	141	143	144	144
Number of polymer residues	185	185	185	185	185	185
Average B-factor	19.1	17.4	17.1	15.4	16	15.9
B-factor for ligands	43.4	31.1	36.2	31.8	31	39
B-factor for solvent	29.5	28.6	27.7	25.8	26.2	26.1
RMS(bonds)	0.011	0.012	0.012	0.012	0.013	0.013
RMS(bond angles)	1.75	1.814	1.814	1.822	1.884	1.877
RMS(dihedral angles)	7.153	7.129	7.134	7.326	7.146	7.137

Values for the highest resolution shell are shown in parentheses

PDB	7HNW	7HNX	7HNY	7HNZ	7HO0	7HO1
Fragment ID	Z1446981563	Z133729708	Z44592329	Z1245793018	Z363071686	Z369263636
Wavelength	0.9213	0.9213	0.9213	0.9213	0.9213	0.9213
Resolution range	67.44 - 1.21 (1.24 - 1.21)	41.20 - 1.27 (1.30 - 1.27)	41.26 - 1.28 (1.31 - 1.28)	67.41 - 1.15 (1.18 - 1.15)	67.46 - 1.15 (1.18 - 1.15)	67.51 - 1.17 (1.20 - 1.17)
Space group	I 4	I 4	I 4	I 4	I 4	I 4
Cell (a b c)	95.38 95.38 45.66	95.41 95.41 45.63	95.34 95.34 45.72	95.34 95.34 45.76	95.40 95.40 45.72	95.47 95.47 45.62
Cell (alpha beta gamma)	90.00 90.00 90.00	90.00 90.00 90.00	90.00 90.00 90.00	90.00 90.00 90.00	90.00 90.00 90.00	90.00 90.00 90.00
Total reflections	582943 (3071)	570366 (7018)	568711 (7602)	586812 (325)	584343 (336)	586427 (1005)
Unique reflections	57582 (1540)	53625 (2374)	52395 (2266)	57933 (303)	62017 (332)	61164 (906)
Multiplicity	10.10 (2.00)	10.60 (3.00)	10.90 (3.40)	10.10 (1.10)	9.40 (1.00)	9.60 (1.10)
Completeness (%)	92.10 (50.10)	99.00 (86.90)	98.80 (86.00)	78.80 (8.50)	84.50 (9.20)	88.20 (26.60)
Mean I/sigma(I)	19.30 (1.04)	17.20 (1.02)	16.50 (1.03)	25.90 (1.13)	30.60 (1.06)	25.00 (1.06)
R-merge	0.057 (1.020)	0.068 (1.073)	0.077 (1.256)	0.044 (0.563)	0.034 (0.277)	0.044 (0.489)
R-rim	0.017 (0.805)	0.020 (0.720)	0.023 (0.793)	0.013 (0.563)	0.010 (0.277)	0.013 (0.467)
CC-half	0.998 (0.399)	0.999 (0.309)	0.999 (0.340)	0.999 (0.175)	1.000 (-)	1.000 (0.553)
R-factor	0.176 (0.350)	0.178 (0.348)	0.178 (0.355)	0.174 (0.326)	0.177 (0.304)	0.175 (0.334)
R-free	0.191 (0.347)	0.196 (0.342)	0.193 (0.363)	0.189 (0.282)	0.196 (0.339)	0.193 (0.350)
Number of total atoms	1735	1725	1738	1734	1736	1739
atoms for ligands	24	23	29	22	24	27
atoms for waters	145	144	143	146	146	146
Number of polymer residues	185	185	185	185	185	185
Average B-factor	16.5	16.6	16.6	14.1	14.1	15.2
B-factor for ligands	32.2	35	37.7	29.3	25.2	37
B-factor for solvent	27.9	28.1	27.2	24	24.4	25.6
RMS(bonds)	0.013	0.013	0.012	0.013	0.013	0.013
RMS(bond angles)	1.869	1.853	1.852	1.853	1.877	1.845
RMS(dihedral angles)	7.136	7.217	7.221	7.189	7.302	7.192

Values for the highest resolution shell are shown in parentheses

PDB	7HO2	7HO3	7HO4	7HO5	7HO6	7HO7
Fragment ID	Z730649594	Z405825414	Z1003146540	Z1266933824	Z3220108246	Z30820160
Wavelength	0.9213	0.9213	0.9213	0.9213	0.9213	0.9213
Resolution range	67.43 - 1.15 (1.18 - 1.15)	67.48 - 1.45 (1.49 - 1.45)	67.39 - 1.21 (1.24 - 1.21)	67.45 - 1.18 (1.21 - 1.18)	67.40 - 1.24 (1.27 - 1.24)	67.46 - 1.30 (1.33 - 1.30)
Space group	I 4	I 4	I 4	I 4	I 4	I 4
Cell (a b c)	95.36 95.36 45.77	95.43 95.43 45.68	95.31 95.31 45.70	95.39 95.39 45.72	95.31 95.31 45.73	95.40 95.40 45.74
Cell (alpha beta gamma)	90.00 90.00 90.00	90.00 90.00 90.00	90.00 90.00 90.00	90.00 90.00 90.00	90.00 90.00 90.00	90.00 90.00 90.00
Total reflections	587535 (445)	473460 (17079)	583600 (3052)	585934 (1471)	578808 (4966)	560400 (9205)
Unique reflections	60406 (428)	36545 (1793)	58889 (1700)	61408 (1189)	55008 (1788)	49717 (2065)
Multiplicity	9.70 (1.00)	13.00 (9.50)	9.90 (1.80)	9.50 (1.20)	10.50 (2.80)	11.30 (4.50)
Completeness (%)	83.00 (12.10)	100.00 (100.00)	94.30 (55.80)	90.90 (35.80)	94.50 (62.70)	98.20 (83.30)
Mean I/sigma(I)	20.70 (1.04)	19.00 (2.00)	21.20 (1.10)	22.70 (1.05)	16.70 (1.05)	19.20 (1.05)
R-merge	0.054 (0.717)	0.314 (8.848)	0.061 (0.890)	0.048 (0.608)	0.075 (1.446)	0.063 (1.240)
R-rim	0.016 (0.717)	0.091 (3.055)	0.018 (0.761)	0.014 (0.570)	0.023 (0.972)	0.019 (0.663)
CC-half	0.999 (0.059)	0.999 (0.152)	1.000 (0.328)	1.000 (0.511)	0.999 (0.228)	0.999 (0.328)
R-factor	0.178 (0.358)	0.167 (0.241)	0.175 (0.329)	0.179 (0.344)	0.175 (0.333)	0.184 (0.362)
R-free	0.192 (0.328)	0.185 (0.251)	0.194 (0.300)	0.196 (0.351)	0.192 (0.325)	0.204 (0.361)
Number of total atoms	1757	1726	1739	1733	1617	1730
atoms for ligands	49	24	27	25	24	24
atoms for waters	142	144	146	142	35	140
Number of polymer residues	185	185	185	185	185	185
Average B-factor	14.6	16.5	15.1	15	16	18.1
B-factor for ligands	31	30.8	44.7	31.9	34.3	36.2
B-factor for solvent	24.8	27.4	25.3	25.5	22.8	29.2
RMS(bonds)	0.012	0.013	0.013	0.013	0.013	0.012
RMS(bond angles)	1.849	1.956	1.876	1.832	1.879	1.79
RMS(dihedral angles)	7.225	7.186	7.168	7.192	7.317	7.179

Values for the highest resolution shell are shown in parentheses

PDB	7HO8	7HO9	7HOA	9QBA
Fragment ID / Ligand	Z993967070	Z1250132788	Z275165822	AL236 (1)
Wavelength	0.9213	0.9213	0.9213	0.976
Resolution range	47.86 - 1.39 (1.42 - 1.39)	67.50 - 1.26 (1.29 - 1.26)	67.46 - 1.35 (1.39 - 1.35)	58.67 - 1.45 (1.47 - 1.45)
Space group	I 4	I 4	I 4	P 6_2
Cell (a b c)	95.63 95.63 45.75	95.46 95.46 45.72	95.40 95.40 45.74	67.75 67.75 71.1
Cell (alpha beta gamma)	90.00 90.00 90.00	90.00 90.00 90.00	90.00 90.00 90.00	90.00 90.00 120.00
Total reflections	387308 (39166)	576125 (6431)	539021 (12511)	681895 (31898)
Unique reflections	40439 (5308)	54627 (2310)	45247 (2196)	32898 (1643)
Multiplicity	9.60 (7.40)	10.50 (2.80)	20.70 (19.0)	20.70 (19.40)
Completeness (%)	96.70 (88.10)	98.10 (80.70)	100.00 (99.20)	100.00 (100.00)
Mean I/sigma(I)	15.10 (2.02)	16.50 (1.04)	10.70 (1.03)	19.50 (1.82)
R-merge	0.133 (2.283)	0.073 (1.107)	0.122 (2.112)	0.077 (1.479)
R-rim	0.044 (0.849)	0.022 (0.785)	0.035 (0.946)	0.018 (0.349)
CC-half	0.998 (0.423)	1.000 (0.322)	0.998 (0.303)	0.998 (0.819)
R-factor	0.176 (0.296)	0.179 (0.350)	0.181 (0.354)	0.150 (0.201)
R-free	0.197 (0.257)	0.199 (0.363)	0.201 (0.334)	0.162 (0.222)
Number of total atoms	1732	1620	1739	1584
atoms for ligands	21	20	29	29
atoms for waters	145	34	144	123
Number of polymer residues	185	185	185	179
Average B-factor	13.6	16.4	17.8	18.6
B-factor for ligands	26.1	35.4	44	18
B-factor for solvent	24	23.9	28.4	30
RMS(bonds)	0.012	0.012	0.011	0.004
RMS(bond angles)	1.825	1.84	1.735	1.355
RMS(dihedral angles)	7.090	7.073	7.059	7.190

Values for the highest resolution shell are shown in parentheses

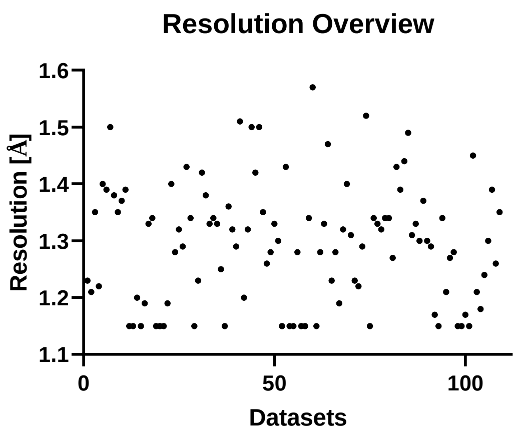

Supplement: Supplementary file 1 — Supplement Information [file 42004_2025_1574_MOESM1_ESM.pdf]
